# A scoping review of health-related stigma outcomes for high-burden diseases in low- and middle-income countries

**DOI:** 10.1186/s12916-019-1250-8

**Published:** 2019-02-15

**Authors:** Jeremy C. Kane, Melissa A. Elafros, Sarah M. Murray, Ellen M. H. Mitchell, Jura L. Augustinavicius, Sara Causevic, Stefan D. Baral

**Affiliations:** 1 Department of Mental Health, Johns Hopkins Bloomberg School of Public Health, 624 North Broadway, Baltimore, MD 21205 USA; 2 Department of Neurology, Johns Hopkins School of Medicine, Sheikh Zayed Tower, Room 6005, 1800 Orleans Street, Baltimore, MD 21205 USA; 3 International Institute for Social Studies, Erasmus University, Kortenaerkade 12, 2518 AX The Hague, Netherlands; 4 Department of Public Health Sciences, Karolinska Institutet, Widerströmska huset, Tomtebodavägen 18A, 171 77 Stockholm, Sweden; 5 Department of Epidemiology, Johns Hopkins Bloomberg School of Public Health, 615 N. Wolfe Street, Baltimore, MD 21205 USA

**Keywords:** Stigma, Low- and middle-income countries, HIV, Tuberculosis, Epilepsy, Depression, Substance use, Scoping review

## Abstract

**Background:**

Stigma is associated with health conditions that drive disease burden in low- and middle-income countries (LMICs), including HIV, tuberculosis, mental health problems, epilepsy, and substance use disorders. However, the literature discussing the relationship between stigma and health outcomes is largely fragmented within disease-specific siloes, thus limiting the identification of common moderators or mechanisms through which stigma potentiates adverse health outcomes as well as the development of broadly relevant stigma mitigation interventions.

**Methods:**

We conducted a scoping review to provide a critical overview of the breadth of research on stigma for each of the five aforementioned conditions in LMICs, including their methodological strengths and limitations.

**Results:**

Across the range of diseases and disorders studied, stigma is associated with poor health outcomes, including help- and treatment-seeking behaviors. Common methodological limitations include a lack of prospective studies, non-representative samples resulting in limited generalizability, and a dearth of data on mediators and moderators of the relationship between stigma and health outcomes.

**Conclusions:**

Implementing effective stigma mitigation interventions at scale necessitates transdisciplinary longitudinal studies that examine how stigma potentiates the risk for adverse outcomes for high-burden health conditions in community-based samples in LMICs.

**Electronic supplementary material:**

The online version of this article (10.1186/s12916-019-1250-8) contains supplementary material, which is available to authorized users.

## Background

Stigma is a major social determinant of health that drives morbidity, mortality, and health disparities [1], and has been described by the World Health Organization as a ‘hidden’ burden of disease [2]. Stigma is characterized by cognitive, emotional, and behavioral components and can be reflected both in the attitudes, often conceptualized as perceived, anticipated, or internalized stigmas, and experiences, including enacted or experienced stigmas affecting a particular trait, among individuals [3–5]. Perceived stigma refers to a person’s understanding of how others may act towards, and think or feel about, an individual with a certain trait or identity [6]. Anticipated stigma refers to expectations of stigma experiences happening in the future [7]. Internalized stigma refers to the individual level process of awareness, acceptance, and application of stigma (to oneself) [8–10]. Finally, experienced or enacted stigma refers to discriminatory acts or behaviors [11].

Stigma adversely impacts individual health outcomes as well as related ‘life chances’, including educational opportunities, employment, housing, and social relationships [1]. It has also been shown to negatively affect help- and treatment-seeking behaviors, hindering the ability of public health agencies to treat and prevent stigmatized health conditions [12]. HIV-related stigma, in particular, has been cited as one of the most enduring barriers to ending the HIV pandemic [13, 14]. Yet, while HIV-related stigma has received greater attention, tuberculosis (TB), mental, neurological, and substance use disorders are also highly stigmatized drivers of the global burden of disease, with significant unmet treatment needs in low- and middle-income countries (LMICs) [15–20].

Hatzenbuehler et al. [1] argued that research on stigma and health outcomes is inappropriately siloed within specific disease/disorder domains. Across health disciplines, this separation has limited the ability to understand the overall impact of stigma on individual wellbeing and on global disease burden [1]. Research siloes have also restricted our ability to develop interventions addressing stigma, particularly in LMICs and among at-risk populations (e.g., lesbian, gay, bisexual, transgender, queer populations (LGBTQ); racial/ethnic minorities; refugees) for whom effective interventions are needed. Despite considerable progress in stigma research over the past decade, a critical review of the literature on the consequences of stigma across health conditions has not been undertaken.

This paper presents a scoping review of the literature on the health consequences of stigma at both the individual and healthcare system levels in LMICs. The review focuses on the main drivers of disease burden in LMICs, namely HIV, TB, mental health, epilepsy, and substance use. The purpose is to summarize recent research on the association between stigma and these conditions, including the direct impact of stigma on affected individuals and its indirect impact on health systems according to help-seeking behavior or service utilization. In so doing, this review highlights commonalities across conditions as well as the key mediators and moderators of the relationship between stigma and health, and identifies at-risk and vulnerable groups. Finally, the strengths and limitations of the current state-of-the-science are highlighted, and recommendations are made for future studies measuring the health-related outcomes of stigma, their pathways, and approaches for evidence-based interventions in LMICs.

## Methods

### Search strategy, data charting, and data summary

We conducted a scoping literature review [21] to summarize current research on stigma and health in relation to five high-burden conditions in LMICs, highlighting the gaps and informing future directions [22]. Five searches of peer-reviewed manuscripts published between 2008 and 2017 were conducted between November 2017 and February 2018 using the PubMed (MEDLINE), PsychINFO, and EMBASE databases. Searches included terms related to (1) ‘stigma’ or other associated terms such as ‘discrimination’; (2) ‘LMICs’, including all countries with this classification according to the World Bank; and (3) specific diseases or disorders. Epilepsy was selected to represent neurologic disorders due to the lack of stigma data related to other neurologic conditions. Additional file 1 includes the full list of search terms for each database searched.

Each review and synthesis was conducted by a single study author with condition-specific expertise. An initial title and abstract review was performed, followed by full-text review of any article included during the first phase. For charting, data were extracted according to study authors and year of publication, study design, sample size and sampling characteristics, type of stigma measured (i.e., perceived, anticipated, internalized, experienced/enacted), strength and significance, if applicable, of the stigma and health outcome association, and mediators or moderators.

In reviewing stigma related to the five diseases/disorders assessed, our team identified three populations most adversely affected by stigma, namely LGBTQ individuals, racial and ethnic minorities, and refugees. Boxes 1, 2 and 3 present further details on the relationship between stigma and health for these populations, focusing on commonalities across disorders.

## Results

### Characteristics of included studies

**Fig. 1:**
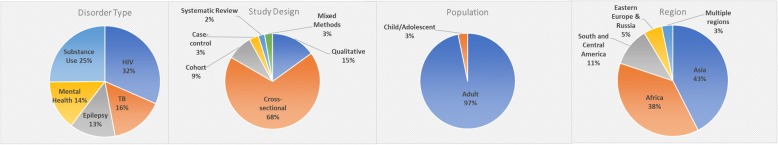
Characteristics of included studies

**Fig. 2:**
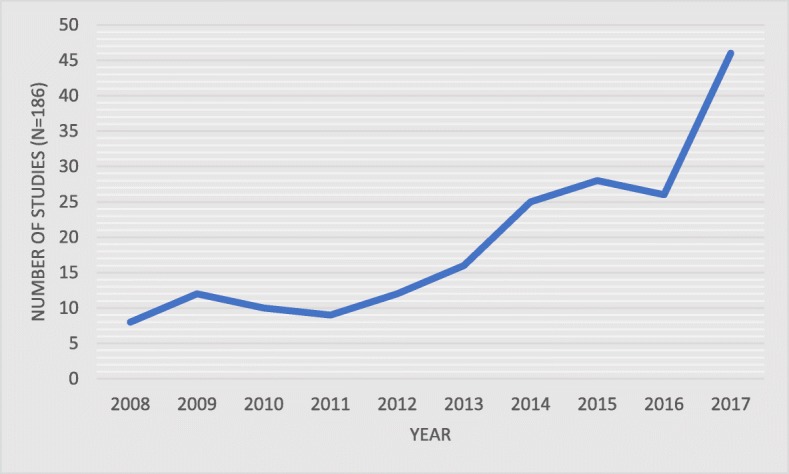
Number of studies included by date of publication

The database search identified a total of 186 articles discussing one or more of the defined diseases and their relationship with stigma, including 59 articles on HIV (32%), 29 on TB (16%), 27 on mental health (14%), 25 on epilepsy (13%), and 46 on substance use (25%) (Fig. 1). Across studies, 52 LMICs were represented, with 79 studies (43%) focusing on Asia, 70 (38%) on Africa, 21 (11%) on South and Central America, 10 (5%) on Eastern Europe and Russia, and 6 (3%) that included more than one region. The most frequently included countries were China (*n* = 30), India (*n* = 21), and South Africa (*n* = 19). Over half of all included studies were published in 2015 or later, with more publications in 2017 than in any other year, suggesting that research attention to stigma is growing (Fig. 2).

Internalized stigma was the most common stigma type measured (44% of studies), whereas fewer studies focused on experienced (enacted), anticipated, or perceived stigma. Children and adolescents were underrepresented in the included studies, with less than 5% of the included studies involving youth populations. Cross-sectional (68%) and qualitative (15%) study designs were most common, and only 9% of studies used longitudinal data.

Herein, a summary of the reviews for each disease/disorder is provided (Tables 1, 2, 3, 4 and 5), followed by a discussion on the overlap and intersection of these stigmas.

**Table 1:** Research on HIV and stigma in LMICs, 2008–2017

Study (First author, year [ref.])	Location	Sampling characteristics	Sample size	Study design	Type of stigma assessed	Description of stigma association (strength, significance)	Significant mediators/moderators
Abboud, 2010 [59]	Lebanon	Convenience sample of PLWHA obtaining care at two hospitals	41	Cross-sectional	Experienced Anticipated Internalized	Strong inverse correlation between stigma scale score and QoL-HIV	None
Bitew, 2016 [56]	Ethiopia	PLWHA seeking care from a hospital	393	Cross-sectional	Perceived stigma	Perceived stigma was associated with suicide attempts	None
Breet, 2013 [36]	South Africa	Convenience sample of PLWHA	210	Cross-sectional	Experienced Anticipated Internalized	HIV stigma and PTSD (*p* < 0.001)	*Med:* Social support
Calabrese, 2016 [26]	Russia	Respondent-driven sampling among individuals who were HIV-positive and reported using injection drugs in past 4 weeks	383	Cross-sectional	Internalized Anticipated	HIV stigma not associated with subjective health rating, but associated with subjective symptom count	*Med:* Injection drug stigma
Carlucci, 2008 [53]	Zambia	PLWHA initiating ART	409	Cross-sectional survey with perceived stigma vs. none assessed at baseline Adherence data obtained over time on ART	Not specified	Perceived stigma present vs. absent (35% vs. 65%; *p* = 0.9)	None
Cluver, 2009 [157]	South Africa	AIDS-orphaned youth vs. non-AIDS orphaned and non-orphaned recruited from schools	1025	Cross-sectional	Not specified	Stigma associated with increased depression, anxiety, PTSD	*Mod:* Food insecurity
Colombini, 2014 [47]	Kenya	Randomly selected from a larger study of HIV+ women obtaining care	48	Qualitative	Not specified	Participants reported that anticipated stigma limited disclosure	None
Denison, 2015 [158]	Tanzania, Uganda, Zambia	PLWHA seeking care at 18 ART facilities	4495	Cross-sectional	Anticipated Internalized	High internalized stigma associated with incomplete adherence	*Mod:* Social support, depression, alcohol abuse
Deribew, 2009 [159]	Ethiopia	HIV and TB patients obtaining clinical care	591	Cross-sectional	Perceived	Negative correlations between stigma and with spiritual, psychological, and social QoL	*Mod:* TB co-infection
Deribew, 2010 [29]	Ethiopia	TB/HIV co-infected patients and HIV non-co-infected patients in three hospitals	620	Cross-sectional	Experienced Anticipated Internalized	Stigma score associated with common mental disorders	None
Dlamini, 2009 [54]	Lesotho, Malawi, South Africa, Swaziland, Tanzania	HIV support groups, clinics, flyers	1457	Cohort	Enacted Internalized	Greater stigma among participants missing medications	*Mod:* Fewer medication worries decreases stigma score
Dow, 2016	Tanzania	Youth (12–24 years) living with HIV attending HIV focused youth clinic	182	Cross-sectional	Experienced Anticipated Internalized	Stigma associated with worse mental health	None
Duff, 2010 [160]	Uganda	Women with HIV attending a PMTCT program	45	Qualitative	Not specified	Stigma cited as common barrier to taking medication	None
Earnshaw, 2014 [5]	South Africa	PLWHA obtaining care at 16 primary care clinics	924	Cohort	Internalized	Self-stigma associated with increased depression, negative condom use attitude, and increased unprotected sex with HIV-positive partners	*Med:* Depression and condom use attitudes mediate association between self-stigma and unprotected sex
Endeshaw, 2014 [30]	Ethiopia	Convenience sampling of PLWHA obtaining care at a clinic	55	Cross-sectional	Internalized Perceived	Stigma associated with depression	None
Erku, 2016 [55]	Ethiopia	Patients on ART and obtaining care from one ART clinic	548	Cohort	Not specified	Perceived stigma associated with decreased adherence Individuals who adhere to ART report decreased stigma over time	None
Garrido-Hernansaiz, 2016 [161]	India	PLWHA recruited through flyers in healthcare settings and NGOs	961	Cross-sectional	Internalized Experienced	Internalized and enacted stigma negatively associated with HQoL	None
Greeff, 2010 [162]	Lesotho, Malawi, South Africa, Swaziland, Tanzania	Purposive sample of PLWHA	1454	Cohort	Experienced Anticipated Internalized	Life satisfaction negatively associated with reported stigma	None
Holzemer, 2009 [60]	Kenya, USA	Convenience sample of HIV infected adults	726	Cross-sectional	Experienced Anticipated Internalized	Stigma accounted for 5.3% of variance in HQoL	None
Kalomo, 2017 [31]	Namibia	PLWHA obtaining care at a clinic	124	Cross-sectional	Experienced Anticipated Internalized	Stigma was significantly associated with depression	None
Kingori, 2012 [24]	Kenya	PLWHA recruited while obtaining care	370	Cross-sectional	Internalized	Felt stigma associated with self-reported poor health, reduced disclosure, and decreased adherence	None
Li, 2009 [33]	Thailand	Hospital-recruited PLWHA	408	Cross-sectional	Internalized Perceived	Depression associated with internalized shame and perceived shame	*Med:* Emotional support
Li, 2014 [163]	Thailand	Convenience sample of PLWHA obtaining care	128	Cross-sectional	Experienced Anticipated Internalized	Stigma negatively associated with adherence	*Mod:* Social support was measured but not significant
Li, 2015 [164]	China	PLWHA recruited from clinics	114	Cross-sectional	Internalized Enacted	Stigma not associated with HQoL	*Med:* Relationship fully mediated by depression
Li, 2016 [165]	China	MSM who were HIV-infected were recruited by local NGO	321	Cross-sectional	Enacted	Enacted stigma associated with increased depression	None
Li, 2017 [35]	China	MSM who were HIV-infected were recruited by local NGO	321	Cross-sectional	Internalized	Self-stigma was associated with depression	*Med:* Positive affect, negative affect, and social support
Liu, 2014 [41]	China	PLWHA who had registered with the CDC	290	Cross-sectional	Experienced Anticipated Internalized	Stigma associated with anxiety	None
Lyimo, 2014 [50]	Tanzania	PLWHA obtaining care at two clinics and on ART for 6 months	158	Cross-sectional	Experienced Anticipated Internalized	Denial of HIV status associated with perceived stigma Self-stigmatization negatively associated with adherence	None
Makin, 2008 [48]	South Africa	Pregnant women living with HIV attending antenatal clinics	293	Cohort (interviews at enrolment and 3 months after giving birth)	Perceived Internalized	Stigma associated with lower likelihood of disclosure	None
Mekuria, 2015 [166]	Ethiopia	PLWHA obtaining care at selected health facilities; selected from national ART-registrar, then randomly selected	664	Cross-sectional	Internalized	HIV-stigma directly associated with all domains of HQoL except physical domain	*Med:* Depression mediates association between stigma and physical HRQoL
Mohite, 2015 [34]	India	Purposive sample of women with HIV attending a care center	50	Cross-sectional	Perceived	Correlation between perceived stigma and depression	None
Nyamathi, 2017 [167]	India	Women with HIV at primary care clinics	400	Cross-sectional	Internalized	Internalized stigma associated with HQoL (*p* < 0.0001)	None
Ojikutu, 2016 [49]	Thailand, Brazil, Zambia	Women with HIV receiving care	299	Cohort	Anticipated	Decreased disclosure associated with anticipated stigma	*Mod:* Cohabitation and marital status
Olley, 2016 [46]	Nigeria	PLWHA obtaining follow-up care at one hospital	139	Cross-sectional	Experienced Anticipated Internalized	Perceived stigma associated with decreased self-disclosure	*Med:* Anticipated discrimination
Olley, 2017 [43]	Nigeria	PLWHA obtaining care at two hospitals	502	Cross-sectional	Experienced Anticipated Internalized	Stigma associated with severe depression	None
Peitzmeier, 2015 [25]	Gambia	PLWHA attending support groups	317	Cross-sectional	Experienced Internalized	Enacted stigma in healthcare setting associated with avoiding or delaying care and not using ART Enacted stigma in household and internalized stigma associated with poorer self-reported health status	None
Peltzer, 2011 [168]	South Africa	Treatment-naïve patients from three public hospitals	735	Cohort	Internalized	HQoL not predictive of stigma	None
Rael, 2017 [37]	Dominican Republic	Purposive sample of women with HIV, female sex workers and control group of women without HIV and non-female sex workers	876	Cross-sectional	Internalized	Internalized stigma associated with increased depression	None
Robinson, 2015 [44]	Turks and Caicos	Data analyzed from 2011 Knowledge, Attitudes, Practices and Behaviors Survey	837	Cross-sectional	Enacted	Self-reported HIV discrimination related to willingness to disclose HIV status	None
Rodriguez, 2017 [57]	South Africa	Pregnant women with HIV obtaining care at a clinic	673	Cross-sectional	Internalized	Stigma associated with suicidality	*Med:* Physical intimate partner violence
Sanjobo, 2008 [169]	Zambia	PLWHA obtaining care at ART centers	60	Cross-sectional	Not specified	HIV stigma was a barrier to adherence	None
Shrestha, 2017 [38]	Malaysia	Prisoners with HIV and opioid dependence who are prisoners	301	Cross-sectional	Experienced Anticipated Internalized	HIV-related stigma was associated with depression (*p* < 0.001); no direct association between stigma and HQoL	*Med:* Depression mediated stigma and HQoL *Mod:* Social support moderated stigma and HQoL
Steward, 2008 [62]	India	PLWHA on ART obtaining care at a large, urban, private hospital	229	Cross-sectional	Enacted	Enacted stigma associated with disclosure avoidance and depression	*Med:* Stigma and depression mediated by use of coping strategies to avoid disclosure of HIV status
Subramanian, 2009 [170]	India	PLWHA obtaining care at one government clinic	646	Cross-sectional	Experienced Anticipated Internalized	All stigma domains (perceived stigma, internalized stigma, and actual stigma) associated with all domains of HQoL instrument (physical, psychological, social and environmental)	None
Takada, 2014 [171]	Uganda	Selected sample of PLWHA from ongoing cohort study	422	Cohort	Internalized	Lagged internalized stigma associated with depression	None
Tao, 2017 [39]	China	MSM newly diagnosed with HIV	367	Cross-sectional	Experienced Anticipated Internalized	Stigma associated with depression; strongest associated was between internalized stigma and depression	None
Tesfaw, 2016 [42]	Ethiopia	PLWHA obtaining care from one hospital	417	Cross-sectional	Perceived	Stigma associated with depression	None
Tesfay, 2015 [61]	Ethiopia	Randomly selected PLWHA on ART with regular follow up at an HIV clinic	594	Cross-sectional	Perceived	Stigma associated with psychological HQoL	*Med:* Gender
Tsai, 2013 [45]	Uganda	Treatment-naïve patients obtaining care at a clinic	259	Cohort	Internalized	Stigma associated with decreased disclosure to household members	*Med:* Social distance
Turan, 2015 [172]	Kenya	Pregnant women with HIV obtaining care at an antenatal clinic	135	Cohort	Experienced Anticipated Internalized	Decreased linkage to care predictive of increased stigma Increased stigma associated with increased depression	None
Valencia-Garcia, 2017 [52]	Peru	Pregnant women with HIV	15	Qualitative	Enacted	Healthcare stigma reduced participants’ willingness to return for care	None
Valenzuela, 2015 [51]	Peru	Patients initiating care at a national referral center Cases: out of care for > 12 months, Controls: those in care	176	Case–control	Experienced Anticipated Internalized	Enacted stigma associated with and being out of care	None
Wu, 2008 [40]	Peru	Women with HIV initiating ART	78	Cross-sectional	Experienced Anticipated Internalized	Stigma associated with depression	*Mod:* Food scarcity
Wu, 2015 [173]	China	PLWHA obtaining care at two hospitals	190	Cross-sectional	Experienced Anticipated Internalized	Higher QoL associated with lower levels of stigma	None
Wu, 2015 [174]	China	MSM with HIV listed in the CDC register	184	Cross-sectional	Experienced Anticipated Internalized	Stigma associated with suicidal ideation	None
Yi, 2015 [27]	Cambodia	PLWHA recruited through cluster sampling method of provinces and HIV clinics	1003	Cross-sectional	Perceived	HIV-related stigma and discrimination associated with higher levels of mental disorders	None
Zhang, 2015 [23]	China	Persons living with HIV were randomly selected for participation from a parent study	2987	Cross-sectional	Experienced Anticipated Internalized	Internalized stigma negatively associated with self-rated health status	*Med:* Resilience
Zhang, 2016 [32]	China	Persons living with HIV were randomly selected for participation from a parent study	2987	Cross-sectional	Experienced Anticipated Internalized	Enacted perceived and internalized stigma were associated with anxiety, depression, decreased resilience, and decreased self-esteem Perceived stigma associated was associated with increased drug use	*Mod:* Income
Zhou, 2017 [58]	China	Persons living with HIV were randomly selected for participation from a parent study	2987	Cross-sectional	Experienced Anticipated Internalized	Stigma negatively associated with QoL	*Med:* HIV symptom management self-efficacy

*ART* antiretroviral therapy, *CDC* Centers for Disease Control, *HQoL* health-related quality of life, *Med* mediators, *Mod* moderators, *MSM* men who have sex with men, *NGO* non-governmental organization, *PLWHA* persons living with HIV and aids, *PMTCT* prevention of mother-to-child transmission, *PTSD* post-traumatic stress disorder, *QoL* quality of life, *TB* tuberculosis

**Table 2:** Research on TB and stigma in LMICs, 2008–2017

Study (First author, year [ref.])	Location	Sampling characteristics	Sample size	Study design	Type of stigma assessed	Description of stigma association (strength, significance)	Significant mediators/moderators
Atre, 2011 [83]	India	Participants without TB in the general population of Western Maharashtra, India, were interviewed from six randomly selected villages	160	Cross-sectional	EMIC interviews with same-sex and cross-sex vignettes depicting a person with typical features of TB	Non-disclosure of disease was associated with fear of losing social status, marital problems, and hurtful behavior by the community	*Mod:* Among females, heredity was perceived as a cause for stigmatization; males reported marital problems in response to the vignette; men perceived greater spousal support than women, who viewed support as more conditional
Chang, 2014 [175]	Global	Descriptive studies	83 studies	Systematic review	Influence of TB stigma on knowledge, attitudes, and responses to TB	Negative attitude and misperceptions of causes of TB were associated with stigma as was TB’s association with HIV Illness disclosure and help-seeking were influenced by stigma	*Mod:* Cultural variations were found for TB-related stigma across countries
Chikovore, 2014 [176]	Malawi	8 focus group discussions with general community members; 2 with health workers Individual interviews with TB patients and chronic coughers	34	Qualitative	Perceived stigma	A compound stigma emerged related to beliefs that cough was a ‘serious’ illness and that a concern among men was failure to perform role expectations, which resulted in mental distress	None
Coreil, 2010 [66]	Haiti	Community residents recruited from community locations, TB patients, and healthcare providers recruited from healthcare centers	101	Qualitative	EMIC, internal stigma, external actions	Stigma was associated with poverty, poor nutrition, and HIV infection	None
Courtwright, 2010	Global	Studies that measured or characterized TB stigma, measured impact of TB stigma on outcomes, or described interventions were included	69 studies	Systematic review	Perceived, internalized, experienced stigma	Fear of infection was most common cause of stigma; TB stigma associated with adverse socioeconomic outcomes; TB stigma is perceived to be associated with adverse treatment-seeking outcomes (diagnostic delay and non-compliance)	*Mod*: Socioeconomic consequences of TB stigma are more acute among women
Cramm, 2011 [177]	South Africa	Area-stratified sampling of households in suburban South Africa One adult of each household randomly chosen to complete survey	1020	Cross-sectional	Modified AIDS-related stigma scale for TB including domains of social identity, blame, shame, avoidance, social sanction	Participants who had stigmatizing views of TB had preferences for special TB queues, treatment provision at clinics (vs. TB hospitals or at home) and held negative views of information provision on TB at work or school and disability grants for TB patients	None
Cremers, 2015, 2016 [178, 179]	Zambia	TB patients were interviewed in a local clinic and surrounding areas	300	Mixed methods	Anticipated, internalized, experienced	Stigma was precipitated by perceptions on co-infection with HIV, perceived immoral behavior, perceived incurability, and traditional beliefs about causes of TB Outcomes of stigma included low self-esteem, discrimination, social exclusion, decreased quality of life, and poor treatment adherence/compliance	*Mod*: Women reported more problems associated with stigma compared to men
Daftary, 2014 [79]	South Africa	Focus groups were conducted with patients receiving treatment for MDR-TB or XDR-TB	23	Qualitative	Not specified	Stigma was associated with poor adherence to MDR-TB and XDR-TB treatment adherence	None
Dhuria, 2009 [84]	India	TB patients were recruited from two DOTS centers in an urban area; controls were recruited from the community and matched by age, gender, and SES	180	Case–control	Not specified	Social domain of the quality of life scale differed significantly between cases (TB patients) and controls (non-TB patients)	None
Dodor, 2009 [70]	Ghana	Interviews and focus groups were held with community members and TB patients	100 interviews; 22 focus groups	Qualitative	Not specified	Five health professional practices were associated with stigmatization of patients, including exclusionary practices, health professional behaviors, discourse around TB, food safety/hygiene, prohibition of burial rites. Stigma may be associated with poor treatment-seeking and diagnostic delay, and poor adherence	None
Finnie, 2011 [150]	Sub-Saharan Africa	Studies were included that collected data on patient and health care system delay in diagnosing and treating TB among patients 15 and older in sub-Saharan Africa	20 studies	Systematic review	Not specified	Stigma of being perceived to have HIV was associated with poor TB treatment seeking	None
Hassard, 2017 [76]	Uganda	Patients in continuation phase of treatment for Pulmonary TB were included using systematic sampling in TB clinics	201	Cross-sectional	Not specified	39% of TB patients did not want anyone to know their status Perceptions of being rejected by the community were associated with non-adherence to TB treatment	None
Hayes-Larson, 2017 [87]	Lesotho	Baseline data from a mixed methods cluster randomized trial of HIV-TB co-infected patients	371	Cross-sectional	Not specified	Greater TB stigma associated with depression Greater external HIV and TB stigma associated with hazardous/harmful alcohol use	None
Isaakidis, 2013 [81]	India	Patients receiving treatment for MDR-TB and HIV purposively selected to represent range of gender, SES, and treatment phase	12	Qualitative	Not specified	Patients considered both TB and HIV to be stigmatizing but HIV more so Stigma associated with not disclosing disease status, lack of mobilization of support systems, and reduced treatment seeking and adherence	None
Juniarti, 2011 [180]	Global	Included qualitative and mixed methods studies focusing on stigma and TB	30 studies	Systematic review	Not specified	Three themes were identified across studies – ‘shame’ of having TB (perceived as a ‘dirty’ disease), ‘isolation’ (due to social exclusion and withdrawal from social contact), and ‘fear’	None
Kipp, 2011 [72]	Thailand	TB patients who started treatment within the past month were recruited from hospital-based TB clinics; a convenience sample of community members without TB was also recruited	780	Cross-sectional	Perceived TB stigma, experienced TB stigma, perceived HIV stigma	Co-infection with HIV, HIV stigma, and lower level of education were associated with greater TB stigma among patients	None
Kipp, 2011 [77]	Thailand	TB patients who started treatment within the past month were recruited from hospital-based TB clinics	459	Cohort	Experienced and perceived TB and HIV stigma	Stigma had a minimal association with adherence to TB treatment overall	*Mod:* Among women and patients with HIV co-infection, experienced stigma was associated with worse adherence
Kumwenda, 2016 [181]	Malawi	Community members, TB patients, and health workers participated in focus group discussions and in-depth interviews	114	Qualitative	Not specified	Stigma was associated with fear over confidentiality of diagnosis, delays in health seeking	*Mod:* Gender
Kurspahić-Mujčić, 2013 [63]	Bosnia and Herzegovina	TB patients were recruited from a university TB clinic in Sarajevo	300	Cohort	Perceived TB stigma	26% of patients reported that TB was a stigmatizing disease The average time interval from first TB symptoms to first healthcare visit was 6.41 weeks among those who perceived TB to be stigmatizing compared to 4.99 weeks among those who did not perceive TB to be stigmatizing	*Mod*: Females were more likely to report TB was stigmatizing than males
Mavhu, 2010 [182]	Zimbabwe	Participants from a parent study who had a chronic cough and had not previously reported their symptoms to the study team or received other healthcare were recruited for in-depth interviews and focus groups	40	Qualitative	Not specified	Participants reported an expectation of being mistreated and stigmatization by clinic staff Perceived association between TB and HIV was associated with delayed treatment seeking	None
Méda, 2014 [73]	Burkina Faso	TB and HIV patients were recruited from health centers and NGOs	1030	Cross-sectional	Not specified	Stigma was associated with treatment adherence	None
Miller, 2017 [183]	Tanzania	Focus group discussions were held with TB patients and their household members	48	Qualitative	Not specified	Domains of stigma described by participants included fear, social isolation, loss of social status, and discrimination perpetrated by healthcare providers Stigma was described as a barrier to care resulting in treatment-seeking delay	*Mod:* Women reported stigma associated with perceptions of promiscuity and rejection by their partners; men reported ‘survival challenges’
O’Donnell, 2014 [82]	South Africa	MDR-TB patients were enrolled consecutively on initiation of treatment at a public TB hospital	104	Cohort	Not specified	Knowledge, attitudes, and beliefs, including HIV stigma, were not associated with TB treatment adherence 6 months later	None
Sima, 2017 [85]	Ethiopia	Systematic sampling of households in randomly selected villages in a pastoralist and a neighboring sedentary community	584	Mixed methods	Perceived TB stigma	Participants reported that TB is less stigmatized than HIV Pastoralists were more likely to have stigma towards TB patients, more likely to feel ashamed if they had TB, and more likely to reject someone with TB in their community than those from sedentary community	None
Skinner, 2016, 2016 [184, 185]	South Africa	TB patients were recruited from a parent study, including those who had remained treatment adherent and those who were initially lost to follow-up	41	Qualitative	Not specified	Stigma and the connection between TB and HIV were associated with not starting treatment and loss to follow-up Greater stigma was associated with MDR-TB; the creation of a discrete TB service for patients reduced stigma; having someone close to them who was on TB treatment also reduced stigma; some participants expressed anger and also resistance to the stigma	None
Somma, 2008 [65]	Bangladesh, India, Malawi, Colombia	Interviews were conducted with TB patients at clinics within each site	427	Cross-sectional	Interviews were conducted with the EMIC	Stigma index varied across countries and was highest in India; stigma was associated with marital prospects among women in India and Malawi	None
Sommerland, 2017 [186]	South Africa	Representative sample of healthcare workers was recruited from 6 hospitals	804	Cross-sectional	Perceived stigma	Significant inverse relationship between perceived stigma/negative attitudes of colleagues and the use of occupational healthcare units for TB screening	None
Xu, 2017 [69]	China	Multi-stage randomized sample of TB patients receiving treatment at home	342	Cross-sectional	Experienced stigma	Experienced stigma was significantly associated with psychological distress	None
Yan, 2017 [75]	China	Multi-stage randomized sample of TB patients from TB dispensaries in three counties	1342	Cross-sectional	Experienced stigma	TB-related stigma and depression were common and both were associated with poor treatment adherence	None

*DOTS* directly observed treatment, short-course, *EMIC* Explanatory Model Interview Catalogue, *MDR-TB* multi-drug resistant tuberculosis, *NGO* non-governmental organization, *SES* socioeconomic status, *XDR-TB* extensively drug resistant tuberculosis

**Table 3:** Research on mental health and stigma in LMICs, 2008–2017

Study (First author, year [ref.])	Location	Sampling characteristics	Sample size	Study design	Type of stigma assessed	Description of stigma association (strength, significance)	Significant mediators/moderators
Adewuya, 2009 [94]	Nigeria	Facility-based sample; any disorder	342	Cross-sectional	Internalized (ISMI)	Poor medication adherence for high relative to low stigma	None
Assefa, 2012 [93]	Ethiopia	Facility-based sample; schizophrenia	212	Cross-sectional	Internalized (ISMI)	Discontinuation of psychotropic medication for high relative to low stigma Psychotic symptoms for high relative to low stigma Suicide attempt for those with high relative to low stigma	None
Bifftu, 2014, 2014 [95, 187]	Ethiopia	Facility-based sample; schizophrenia	411	Cross-sectional	Perceived (PDD), resistance (ISMI-SR)	Poor antipsychotic medication adherence for high perceived relative to low perceived stigma and for high relative to low stigma resistance Duration of illness less than 1 year for high relative to low perceived stigma (NS for stigma resistance) Poor follow-up care NS for perceived stigma or stigma resistance	None
Cai, 2017 [188]	China	Facility-based sample; schizophrenia	172	Cross-sectional	Internalized (ISMI)	Stigma not associated with quality of life	None
Dardas, 2017 [106]	Jordan	School-based; depression	2349	Cross-sectional	Personal and perceived (DSS)	Stigma associated with care seeking	*Mod:* Significant interaction between stigma and depression for willingness to seek help
Devi Thakoor, 2016 [189]	China, Mauritius	Facility-based sample; SMI	300	Cross-sectional	Internalized (ISMI)	Duration of psychosis of greater than 3 months relative to less than 3 months was associated only with the following ISMI items: increased perceived break up due to illness and increased perceived disinheritance due to illness by family (China); decreased patient awareness of illness and decreased family awareness of illness (Mauritius)	None
Elkington, 2010 [92]	Brazil	Facility-based sample; SMI	98	Cross-sectional	Internalized, experienced discrimination, perceived (SPISEW)	Significantly higher mean personal experiences of stigma score for individuals in the mild to moderate vs. moderate to marked illness severity group Perceived attractiveness and relationship discrimination stigma scales were NS HIV risk and protective behaviors associated with relationship discrimination for sexual activity, unprotected sex, and fewer partners; perceived attractiveness – all NS; personal experiences – all NS	None
Fawzi, 2016 [91]	Egypt	Facility-based sample; depression	196	Cohort	Internalized (ISMI)	Treatment acceptance: patients refusing treatment had a higher stigma score than those who accepted treatment Diabetes: increase in ISMI score was associated with change in fasting plasma glucose and standardized 8-week percentage change in HbA1c levels in multiple regression analyses	None
Fresan, 2017 [190]	Mexico	Facility-based sample; schizophrenia	217	Cross-sectional	Perceived and experienced discrimination (KSS)	Length of hospitalization increase of 1 week associated with KSS score Duration of untreated psychosis was NS	None
Grover, 2017 [99]	India	Facility-based sample; SMI	1403	Cross-sectional	Internalized (ISMI)	Shorter duration of illness was significantly correlated with higher overall internalized stigma among patients with schizophrenia, but the SE and DE subscales were NS; overall stigma and all subscales were NS among patients with recurrent depressive disorder Shorter duration of treatment was significantly correlated with higher overall internalized stigma among patients with schizophrenia, but the SE and SR subscales were NS Among patients with recurring depression, higher overall internalized stigma was significantly correlated, but the SE, DE, and SR subscales were NS Lesser symptom severity among patients with schizophrenia, as measured by the PANSS-P, was significantly correlated with overall stigma and only the SR subscale was NS; however, the PANSS-N and PANSS general psychopathology scales were NS with overall stigma For patients with depression as measured by the HDRS, overall stigma was significantly correlated, but not the SE, SW, or SR subscales Greater participation restriction was significantly correlated with overall stigma score and all subscales among patients with schizophrenia; for patients with depression, overall stigma score was significant, but the A and SE subscales were NS	None
Koschorke, 2014 [101]	India	Schizophrenia	282	Cross-sectional	Anticipated and experienced discrimination (DISC)	Symptom severity, as measured by total PANSS score, was NS in association with discrimination; however, belonging in a higher PANSS-N quartile was associated with reduced odds of experiencing negative discrimination, while belonging in a higher PANSS-P quartile was associated with increased odds of experiencing negative discrimination	None
Kulesza, 2014 [102]	India	Facility-based sample; majority exhibited depression	60	Cross-sectional	Anticipated and perceived (EMIC-SS)	Symptom severity for depression was positively correlated with stigma	None
Lahariya, 2010 [97]	India	Facility-based sample; SMI	295	Cross-sectional	One question on fear of stigma related to care seeking	Delay in care seeking: 73% of patients had delayed seeking care at least in part due to a fear of stigma	None
Li, 2017 [88]	China	Facility-based sample; schizophrenia	384	Cross-sectional	Internalized (ISMI)	Psychiatric symptoms: Stigma significantly increased with an increase in general symptoms measured via the BPRS in multiple regression analyses; PANSS-N NS Functioning: Stigma score significantly decreased with an increase in GAF in multiple regression analyses Quality of life: Stigma score significantly increased b = 0.01 (0.01–0.02) with an increase in SQLS score in multiple regression analyses	None
Loch, 2012 [191]	Brazil	Facility-based sample; mostly SMI	169	Cohort	Question on dangerousness stereotyping	Re-hospitalization: Individuals who were readmitted over the year were significantly more likely to be stereotyped as dangerous by family members that those who were not readmitted	None
Lu, 2012 [192]	China	Facility-based sample; schizophrenia	92	Cross-sectional	Internalized (ISMI); experienced discrimination (MCESQ)	Insight: MCESQ and ISMI total score was NS in multiple regression with insight as the outcome	None
Lv, 2013 [100]	China	Facility-based sample; schizophrenia	95	Cross-sectional	Internalized (ISMI)	Symptom severity: Positive and negative symptoms of psychosis both NS Greater duration of illness was associated with a change in stigma score; greater number of hospitalizations was NS; greater quality of life was associated with a change in stigma score	None
Mosanya, 2014 [98]	Nigeria	Facility-based sample; schizophrenia	256	Cross-sectional	Internalized (ISMI)	Medication side effects, comorbid medical problem, duration of illness, and number of episodes all NS Increase in BPRS score increased the odds of having high vs. low stigma Individual in the high stigma group had significantly lower mean quality of life as measured by all WHOQOL-Brief subscales (physical, psychological, social, and environment) as well as the overall quality of life and general health	None
Rayan, 2017 [103]	Jordan	Facility-based sample; depression	160	Cross-sectional	Perceived (PDD)	Pain was NS An increase in number of relapses was associated with a significant change in stigma score Symptom severity of depression was associated with a significant change in stigma score	None
Rayan, 2017 [104]	Jordan	Facility-based sample; schizophrenia	161	Cross-sectional	Perceived (PDD)	In a multivariate regression, increase in stigma was associated with a significant reduction in quality of life Symptom severity for depression was significantly correlated with stigma	None
Roberts, 2017 [96]	Ukraine	Community-based time-location sampling; depression, anxiety or PTSD	2203	Cross-sectional	One question on stigma related to care seeking	Out of the 703 people with a mental health problem, only 180 (25.6%) had sought care from any medical source (including pharmacists, or NGO counselling center); of the 520 who did not seek care, 41 attributed this to stigma or embarrassment (8%)	None
Sharaf, 2012 [107]	Egypt	Facility-based sample; schizophrenia	200	Cross-sectional	Internalized (ISMI)	In multivariate regression, increase in stigma was associated with increase in suicide risk Insight was correlated positively with stigma	*Mod:* Insight was measured but not a significant moderator of stigma–suicide relationship
Shi-Jie, 2017 [90]	China	Facility-based sample; depression	158	Cross-sectional	Anticipated and perceived (EMIC)	The depression subscale of the SCL-90 was associated with a significant increase in stigma in multivariate regression MADRS, somatization, and the SCL-90 total and anxiety subscale score were all NS; fatigue was associated with a significant increase in stigma in multivariate regression; disability NS in multivariate regression; duration of illness NS in multivariate regression	None
Singh, 2016 [89]	India	Facility-based sample; schizophrenia	100	Cross-sectional	Internalized (ISMI); anticipated and perceived (EMIC)	Functioning was significantly associated with decrease in all ISMI subscales in regression analyses except ISMI-A and ISMI-SR Increase in GAF score was associated with reduced odds of having high vs. low overall ISMI score Functioning was negatively correlated with EMIC score Duration of illness was NS in regression analyses, except an increase in duration was associated with increased odds of having high vs. low ISMI-SR score; treatment duration was NS Symptom severity was NS in regression analyses, except an increase in the general PANSS subscale was associated with increased odds of having high vs. low ISMI-A score	None
Vidojevic, 2015 [193]	Serbia	Facility-based sample; depression	52	Cross-sectional	Anticipated and experienced discrimination (DISC)	Hospitalization history was associated with higher discrimination and lower ability to overcome stigma	None
Wang, 2017 [194]	China	Facility-based sample; schizophrenia	146	Cross-sectional	Perceived and internalized (LSS)	Quality of life positively correlated with perceived stigma and a coping orientation of withdrawal, but NS with secrecy, educating challenging and distancing coping strategies; positively correlated with both stigma-related feelings subscales (misunderstood and different/ashamed) Medication adherence negatively correlated with perceived discrimination and a coping orientation of secrecy, but NS with withdrawal, educating, challenging, and distancing; negatively correlated with feeling different/ashamed but feeling misunderstood NS	None
Xu, 2013 [105]	China	Facility-based sample; schizophrenia	133	Cross-sectional	Self-blame (CSQ-SB)	Symptom severity for depression was predicted by self-blame	None

*BPRS* Brief Psychiatric Rating Scale, *CSQ-SB* Self-Blame subscale of the Coping Style Questionnaire, *DISC* Discrimination and Stigma Scale, *DSS* Depression Stigma Scale, *EMIC-SS* Explanatory Model Interview Catalogue Stigma Scale, *GAF* General Assessment of Functioning, *HDRS* Hamilton Depression Rating Scale, *ISMI* Internalized Stigma of Mental Illness Scale (*-SR* Stigma Resistance subscale, *-A* Alienation subscale), *KSS* King’s Stigma Scale, *LSS* Link’s Stigma Scale, *SE* ‘stereotype endorsement’, *SR* stigma resistance, *DE* discrimination experience, *SW* social withdrawal, *MADRS* Montgomery and Asberg Depression Rating Scale, *MCESQ* Modified Consumer Experiences of Stigma Questionnaire, *Mod* moderator, *NS* not significant, *PANSS* Positive and Negative Syndrome Scale (*-N* negative, *-P* positive), *PDD* Perceived Devaluation and Discrimination Scale, *SCL-90* Symptom Checklist-90, *SMI* serious mental illness, *SPISEW* Stigma of Psychiatric Illness and Sexuality among Women, *SQLS* Schizophrenia Quality of Life Scale, *WHOQOL* World Health Organization Quality-of-Life Scale

**Table 4:** Research on epilepsy and stigma in LMIC, 2008–2017

Study (First author, year [ref.])	Location	Sampling characteristics	Sample size	Study design	Type of stigma assessed	Description of stigma association (strength, significance)	Significant mediators/moderators
Alkhamees, 2013 [195]	Saudi Arabia	Not specified	110	Cross-sectional	Not specified	Stigma associated with overall QoL	None
Aydemir, 2011 [117]	Turkey	People with epilepsy for the past 4 years, compared to people with migraines and people with no symptoms (controls)	172	Case–control	Internalized	Stigma associated with decreased disclosure	None
Bhalla, 2012 [196]	Cambodia	People with epilepsy with controls matched on age, sex, and village	288	Case–control	Internalized	Stigma associated with worse QoL, limitations in work due to epilepsy, and social limitations due to epilepsy	None
Doganavsargil-Baysal, 2017 [112]	Turkey	Adults with epilepsy obtaining care at one outpatient clinic	89	Cross-sectional	Internalized	Stigma associated with lower scores on HQoL and greater psychiatric symptomatology	None
Elafros, 2013 [119]	Zambia	Caregivers of children aged < 8 years with epilepsy obtaining care at local clinics	100	Cross-sectional	Internalized	Maternal stigma associated with psychiatric morbidity and need for psychiatric support; actively limiting child activities	None
Espinola-Nadurielle, 2014 [114]	Mexico	Patients with epilepsy treated at one outpatient clinic and their caregivers	10	Qualitative	Not specified	Stigma associated with social withdrawal	None
Fawale, 2014 [115]	Nigeria	Adult patients with epilepsy treated at an outpatient clinic with age- and sex-matched controls	93	Case–control	Internalized	Stigma associated with worse QoL and worse social function	None
Getnet, 2016 [120]	Ethiopia	Adults with epilepsy on AEDs for at least 3 months obtaining care at outpatient clinics	450	Cross-sectional	Internalized	Perceived stigma associated with worse AED adherence	None
Hamid, 2013 [197]	Jordan	Adult patients with epilepsy obtaining care at an outpatient clinic	45	Cross-sectional	Not specified	Severity of stigma associated with worse mental health QoL	None
Hirfanoglu, 2009 [109]	Turkey	Children with epilepsy (aged 8–17 years) and their parents	533	Cross-sectional	Not specified	Child stigmatization associated with greater negativity about epilepsy, greater perceived lack of support, low self-esteem	None
Iqbal, 2013 [118]	Pakistan	Married women obtaining care at a tertiary center	381	Cross-sectional	Not specified	Stigma associated with concealment of epilepsy from future husbands	None
Komolafe, 2011 [198]	Nigeria	Women with epilepsy obtaining care from local clinics	6 groups of 8–15 women with epilepsy	Qualitative	Not specified		None
Kumari, 2009 [199]	India	People with epilepsy obtaining care at an outpatient clinic, selected randomly	45	Cross-sectional	Internalized, anticipated, enacted	Stigma associated with decreased HQoL	None
Lopez, 2009 [200]	Mexico	Children aged 6–18 years with epilepsy	~200	Cross-sectional	Not specified	Perceived stigma influences QoL	None
Luna, 2017 [116]	Ecuador	Adults with epilepsy or parents of children (aged < 15 years) with epilepsy	143	Cross-sectional	Internalized	Stigma associated with decreased disclosure of epilepsy	None
Nagarathnam, 2017 [201]	India	Adults with epilepsy on an AED for a year	170	Cross-sectional	Not specified	Stigma associated with worse QoL	None
Nehra, 2014 [202]	India	Adults with active epilepsy obtaining care from a clinic	208	Cross-sectional	Experienced, anticipated, internalized	Stigma correlated with worse overall function	None
Saadi, 2016 [203]	Bhutan	Patients with epilepsy obtaining care at a tertiary referral center	172	Cross-sectional	Not specified	Increased stigma associated with lower QoL	None
Tegegne, 2015 [204]	Ethiopia	Adults with epilepsy obtaining care from a hospital-based outpatient clinic	415	Cross-sectional	Internalized	Perceived stigma is associated with increased depression	None
Tsegabrhan, 2014 [205]	Ethiopia	Adults with epilepsy obtaining treatment from one hospital	300	Cross-sectional	Internalized	Stigma associated with increased depression	None
Turki, 2016 [110]	Tunisia	Patients with epilepsy followed by one clinic	20	Cross-sectional	Not specified	Absence of stigma associated with better self-esteem	None
Viteva, 2012 [206]	Bulgaria	‘Representative selection’ of patients with epilepsy at a neurology clinic	164	Cross-sectional	Internalized	Stigmatization frequency and severity correlated with depression	None
Viteva, 2013 [207]	Bulgaria	Consecutive patients with refractory and pharmaco-sensitive epilepsy	246	Cross-sectional	Internalized	Stigma associated with all subscales of QoL except change in health and sexual relations	None
Viteva, 2016 [121]	Bulgaria	Adults with epilepsy obtaining care from one hospital-based clinic	153	Cross-sectional	Internalized	Greater stigma associated with increased reporting of medication side effects	None
Yeni, 2016 [111]	Turkey	Outpatients with epilepsy obtaining care at one university	70	Cross-sectional	Internalized	Stigma associated with increased anxiety, depression, increased effects of disease on life, decreased role functioning, and worse disease-associated attitudes	None

*AED* anti-epileptic drug, *HQoL* health-related quality of life, *QoL* quality of life

**Table 5:** Research on substance use and stigma in LMIC, 2008–2017

Study (First author, year [ref.])	Location	Sampling characteristics	Sample size	Study design	Type of stigma assessed	Description of stigma association (strength, significance)	Significant mediators/moderators
Brittain, 2017 [208]	South Africa	HIV-infected women receiving antenatal care in Cape Town primary care clinic were enrolled when entering PMTCT services	580	Cross-sectional	HIV stigma (non-specified)	Higher HIV-related stigma was associated with reduced odds of alcohol use (*p* < 0.01)	None
Budhwani, 2017 [209]	Dominican Republic	Transgender women who did and did not report recent drug use were recruited and interviewed using a snowball sampling approach	287	Cross-sectional	Experienced stigma	Higher stigma scale score associated with greater odds of recent cocaine use (*p* < 0.01) but not other drug use	None
Capezza, 2012 [144]	Chile	Adults in 10 primary care centers were recruited using a time-limited sampling from a clinical population	2839	Cross-sectional	Perceived stigma/discrimination	Past 6-month discrimination (based on race, sex, age, appearance, disability, sexual orientation, economic status, political affiliation, and/or religion) was associated with significantly higher odds of past 6-month hazardous drinking (*p* = 0.001) and any illegal drug use (*p* < 0.001)	None
Coelho, 2015 [145]	Brazil	Undergraduate students were selected using a two-stage sampling procedure at a university	1264	Cross-sectional	Experienced stigma/discrimination	There was no association between lifetime discrimination and recent alcohol use in the overall sample; however, moderator analyses indicated that last-year students with discrimination had higher odds of alcohol-related problems than first-year students who did not experience discrimination (*p* < 0.05) and those who experienced two or more types of discrimination had higher odds of alcohol-related problems compared to those who experienced no discrimination or discrimination of one type only	*Mod:* Year of study in university (last year students who experienced discrimination had higher odds of alcohol-related problems compared to first year students who did not experience discrimination)
Culbert, 2015 [210]	Indonesia	Stratified random sample of prisoners who were HIV-infected in two prisons in Jakarta	102	Mixed methods	HIV stigma scale (stereotypes, disclosure concerns, self-acceptance, social relationships)	Significantly higher stigma scale scores were reported among participants who were incarcerated for a drug offense, had sought treatment for substance use problems, and those who reported opioid withdrawal symptoms during incarceration	None
Deryabina, 2017 [132]	Kyrgyzstan	Persons with injection drug use were recruited from needle exchange and syringe programs (NSP) and from local NGOs; NSP staff were also interviewed	123	Qualitative	Not specified	‘Fear to be a known drug user’ was commonly cited as barrier to accessing NSP services; concerns about disclosure of using injection drugs were cited including fears of losing employment, social stigma, rejection from family/friends, fear of police, and being treated poorly by healthcare professionals	None
Du, 2012 [127]	China	Persons with injection drug use were recruited from a computerized database and were asked to complete a survey; clients in a methadone maintenance program were invited to participate in focus groups; clinic staff also participated in focus groups	610	Mixed methods	Not specified	Stigma/discrimination was a barrier for persons with injection drug use getting tested for HIV; participants identified stigma both towards their drug use and HIV status; some participants also expressed fear of police and being placed in compulsory drug treatment	None
Fan, 2016 [211]	China	MSM were recruited from local community-based organizations and through snowball sampling	391	Cross-sectional	HIV-related stigma scale (domains: shame, blame, social isolation, discrimination, equity)	MSM who reported any alcohol use also reported significantly higher levels of stigma than non-drinkers; stigma scale scores were highest among those with heavy alcohol use	None
Go, 2016 [212]	Vietnam	PWID who were newly diagnosed with HIV were enrolled from a parent RCT; data were collected at baseline and 1 month later (pre-intervention)	336	Cohort	HIV and drug stigma (non-specified)	Neither HIV nor drug stigma were associated with HIV status disclosure in adjusted models	None
Goldstone, 2017 [213]	South Africa	Mental healthcare workers who worked with persons with substance use disorders and suicidal ideation were interviewed	18	Qualitative	Not specified	Stigma related to substance use, mental illness, and suicide was identified as a barrier to suicide prevention among persons who have substance use disorders	None
Greene, 2013 [214]	China	Clinic-based sample of current or former PWID who were HIV-infected were recruited; caregivers (outside of clinical care) of patients also interviewed	96	Cross-sectional	Patient-level perceived HIV-related stigma; caregiver-level stigma towards HIV	Patient-perceived stigma was associated with poor mental health and a lack of social support among caregivers; caregivers lack of social support was attributable to their own HIV stigma; higher caregiver stigma was also associated with less caregiver self-efficacy	None
Ha, 2015 [147]	Vietnam	Respondent-driven sampling to recruit MSM	451	Cross-sectional	Experienced, perceived, and internalized homosexuality-related stigma	Experienced and perceived stigma were both associated with depression, which in turn predicted drug and alcohol use, and, ultimately, sexual risk behaviors	*Med*: Relationship of stigma and sexual risk behaviors was mediated by depression and alcohol/substance use
Hayes-Larson, 2017 [141]	Lesotho	Baseline data from a mixed methods cluster randomized trial of HIV-TB co-infected patients	371	Cross-sectional	Not specified	25% of the sample reported hazardous/harmful alcohol use; greater external HIV and TB stigma associated with hazardous/harmful alcohol use	None
Heath, 2016 [215]	Thailand	Peer-based recruitment used to recruit participants who had injection drug use in the past 6 months	437	Cross-sectional	Experienced stigma	Experienced stigma, including verbal abuse about their drug use, being discouraged from participating in family activities, and refused medical care by healthcare workers, were associated with avoiding accessing health services	None
Howard, 2017 [124]	South Africa	Street-outreach methods were used to recruit women who use substances for FGDs; primary healthcare and rehab staff were also recruited for FGDs	60	Qualitative	Not specified	Stigma was identified as a barrier to accessing primary care and substance use treatment services for women who use substances	None
Ibragimov, 2017 [138]	Tajikistan	Purposive sampling used in pharmacies to recruit pharmacists and pharmacy students for in-depth interviews	28	Qualitative	Not specified	Themes related to stigma among pharmacists and pharmacy students towards PWID included having negative emotions, connotations, and stereotypes of PWID; examples included support for isolation of PWID and forced treatment, and refusal to provide syringe access and other resources	None
James, 2012 [139]	Nigeria	Medical students who had completed a clerkship in Psychiatry and recent medical graduates were interviewed	254	Cross-sectional	Attitudes Towards Mental Illness Questionnaire	Medical students and recent medical graduates displayed significantly stigmatizing attitudes towards persons who use alcohol and cannabis	None
Jamshidimanesh, 2016 [125]	Iran	Women with substance abuse were recruited from local drop-in center clinics	32	Qualitative	Not specified	Stigma towards addiction was identified as a barrier to healthcare treatment	None
Johannson, 2017 [216]	Estonia	Respondent-driven sampling used to recruit PWID who were HIV infected	312	Cross-sectional	Internalized HIV and drug stigma	Internalized HIV and drug stigma were high; internal drug use stigma was negatively associated with disclosure of drug use to family members (non-parents) and healthcare workers; internalized HIV stigma was positively associated with disclosure to healthcare workers; neither HIV nor drug stigma were associated with disclosure of use to sexual partners, close friends, or parents	*Mod*: Authors investigated interaction of HIV and drug stigma; interaction effects on disclosure were non-significant
Kekwaletswe, 2014 [131]	South Africa	Purposive sample of HIV patients in ART clinics	304	Cross-sectional	Experienced and anticipated HIV stigma	Among those who reported using alcohol, higher levels of HIV stigma were associated with skipping ART doses	None
Kerrigan, 2017 [143]	Brazil	Proportional random sampling of persons with HIV in six public health facilities	900	Cross-sectional	Internalized and experienced HIV stigma (Berger scale)	History of drug use was associated with higher levels of stigma/discrimination	None
Khuat, 2015 [217]	Vietnam	Respondent-driven sampling of women with injection drug use	403	Cross-sectional	Gender-based stigma	Women with injection drug use reported substantial gender-related stigma	None
Krawczyk, 2015 [218]	Brazil	Purposive sample recruited by community leaders of adults who used crack	38	Qualitative	Not specified	Almost all participants reported significant stigmatization due to their crack use, including being labelled as ‘thieves’ or ‘sick’; many also reported discrimination in health services	None
Lan, 2017 [126]	Vietnam	Baseline data from an RCT; participants were persons with injection drug use from 60 randomly selected commune health centers	900	Cross-sectional	Perceived and internalized drug-related stigma	Drug-related stigma was associated with reduced overall access to general healthcare but was not associated with MMT or needle exchange program access	None
Lembke, 2015 [219]	China	Persons who used heroin and were seeking treatment were recruited from a local hospital for in-depth interviews	9	Qualitative	Not specified	All participants reported intense stigma towards persons who use drugs, including social exclusion; participants also reported confidential, anonymous treatment as a facilitator for accessing services	None
Liao, 2014 [220]	China	Mixed recruitment methods (community outreach, snowball sampling) was used to recruit MSM	1230	Cross-sectional	HIV-related stigma scale (domains: shame, blame, social isolation, discrimination, equity)	HIV-related stigma was common among this MSM sample and was associated with increased alcohol use	None
Lim, 2013 [134]	Vietnam	Baseline data from RCT; PWID recruited from active recruiters and peer referral; community members recruited through systematic sampling	3023	Cross-sectional	HIV-related stigma scale (domains: shame, blame, social isolation, discrimination, equity) Drug-related stigma (internalized, perceived, experienced) among PWID; perceptions of PWID among community members	Higher education inequality was associated with more HIV-related stigma among PWID and among community members; lower individual education associated with greater HIV and drug stigma among both PWID and community members; individual level education negated the effect of community-level education inequality; part-time employed PWID reported more perceived and experienced stigma than full-time employed PWID	*Mod*: Cross-level interactions of community and individual predictors that community SES did not vary by individual level SES
Lozano-Verduzco, 2016 [221]	Mexico	Women were recruited from an addiction treatment clinic and through snowball sampling for in-depth interviews	13	Qualitative	Not specified	Women reported experiences of gender-based stigma and stigma related to their substance use; they reported that women who use substances experience significantly more stigma than men Psychiatric comorbidities lead to additional stigmatization; these combined stigmas reduce treatment seeking	None
Luo, 2014 [222]	China	Random sample of households in two communities was conducted	848	Cross-sectional	Community members were asked about labelling, stereotyping, and social distancing in response to vignettes about drug users and non-drug users	Vast majority of participants labelled persons with drug dependence as ‘addicts’ as opposed to other options of ‘normal’ or ‘patient’; persons with drug dependence were stereotyped negatively compared to persons without drug dependence Participants also expressed desire to have significant social distance from persons with drug dependence and a low willingness to interact with them	None
Mattoo, 2015 [223]	India	Purposive sample of persons with alcohol and opioid dependence and one of their family members, recruited from a drug treatment center	200 (100 patient/family member dyads)	Cross-sectional	Perceived drug-related stigma	Perceived stigma about persons who use substances was highly concordant between persons with alcohol and opioid dependence and their family members	None
Mimiaga, 2010 [130]	Ukraine	Participants who were receiving HIV treatment at a local clinic and had been infected through injection drug use were recruited for FGDs	16	Qualitative	Not specified	HIV-related stigma was mentioned by all participants as a barrier to treatment adherence; participants feared that disclosing HIV status would identify them as a person who injects drugs; others reported fear of rejection from family if they disclosed their HIV status; discrimination by healthcare providers was also mentioned as a source of HIV-related stigma	None
Moomal, 2009 [146]	South Africa	Representative sample of South African adults from the South African Stress and Health Survey	4351	Cross-sectional	Acute and chronic discrimination both related and unrelated to race	Acute racial and non-racial discrimination and chronic non-racial discrimination were associated with increased risk for substance use disorders	None
Mora-Rios, 2017 [133]	Mexico	Persons who use drugs and their family members were recruited through psychiatric care facilities; healthcare personnel were also recruited	35	Qualitative	Not specified	Persons who used alcohol and drugs, their family members, and healthcare workers frequently reported family, healthcare personnel, and persons in the street/neighbors as sources of stigma; all persons who used substances reported being an object of social stigma, which was also viewed as a barrier to recovery	None
Myers, 2013 [224]	South Africa	Participants were South Africans who self-identified as Black African or colored who had alcohol or other drug use problems and had sought treatment (cases) or had not sought treatment (controls); cases were recruited from treatment facilities; controls were recruited from the community	434	Case–control	Stigma consciousness scale (perceived drug-related stigma)	There was no association between stigma and alcohol or other drug service use among Black African participants; among colored participants, perceived stigma was associated with increased odds of service use	None
Otiashvili, 2013 [225]	Georgia	Women who used injection drugs were recruited through peer-to-peer and peer-to-professional word-of-mouth for in-depth interviews; purposive sampling was used to recruit healthcare staff	89	Qualitative	Not specified	Participants described intense stigmatization that was a major barrier to treatment seeking and access; stigma was also thought to be a more significant barrier to treatment access among women than among men who use substances	None
Papas, 2017 [142]	Kenya	Baseline data from RCT participants who were HIV-infected outpatients and used alcohol	614	Cross-sectional	HIV-related stigma (public attitudes towards HIV, ostracization, discrimination, personal life disruption)	Women reported higher levels of HIV-related stigma than men; stigma was associated with an increased odds of experiencing sexual or physical violence among both men and women	None
Peacock, 2015 [226]	El Salvador	Respondent-driven sample of MSM and transgender women	670	Cross-sectional	Internalized homonegativity scale	Binge drinking prevalence was high in the overall sample; higher levels of internalized homonegativity were associated with increased binge drinking	None
Rathod, 2015 [227]	India	Community sample recruited through cluster sampling design in a rural district	3220	Cross-sectional	Internalized stigma of mental illness	Stigmatizing belief of shame was commonly reported among those with alcohol use disorders, which may have resulted in a low rate of treatment seeking	None
Ronzani, 2009 [140]	Brazil	Primary healthcare professionals were recruited to participate	609	Cross-sectional	Attitudes towards use of alcohol and other drugs	Alcohol, tobacco, marijuana, and cocaine use were negatively judged behaviors by healthcare professionals relative to other conditions (e.g., mental health problems, HIV); persons with alcohol, marijuana, and cocaine problems suffered the highest rate of service refusal	None
Sarkar, 2017 [135]	India	Persons with alcohol or opioid use disorders were recruited from a treatment facility	201	Cross-sectional	Internalized stigma of mental illness	There were high levels of internalized stigma across study participants; persons with alcohol and opioid use disorder with severe stigma had significantly lower physical, social, psychological, and environmental quality of life scores than those with mild-to-moderate stigma	None
Schensul, 2017 [129]	India	Men living with HIV were recruited from ART treatment centers	361	Mixed methods	Experienced stigma	Men who drank alcohol at higher levels had a greater risk of non-ART adherence; men also reported skipping ART doses when drinking with friends due to fear of HIV status disclosure	None
Sharma, 2017 [228]	India	Purposive sampling to recruit women with non-injection drug use; women who had injection drug use were also recruited from a parent prospective cohort study	48	Qualitative	Not specified	Stigma from healthcare providers was reported as a significant barrier to accessing services	None
Spooner, 2015 [229]	Indonesia	Outreach workers recruited women who had injection drug use	19	Qualitative	Not specified	Women who used injection drugs felt significant stigma and shame; they reported social exclusion, isolation from society and from treatment options; they also reported sharing of needles with small groups of trusted friends	None
Ti, 2013 [128]	Thailand	Peer-based outreach and word-of-mouth recruiting used to recruit persons who injected drugs; sample restricted to those HIV-negative or unknown HIV serostatus	350	Cross-sectional	Experienced stigma	Having been refused healthcare services was associated with avoiding getting an HIV test	None
Van Nguyen, 2017 [137]	Vietnam	Patients taking MMT at one of two MMT sites were recruited	241	Cross-sectional	HIV and drug-related stigma (blame/judgment, shame, discrimination, disclosure, others’ fear of HIV transmission)	Almost all participants reported experiencing blame/judgment, discrimination, and shame Unemployment was associated with discrimination; blame, judgment, and shame were associated with anxiety and depression	None
Yang, 2015 [136]	China	Males with drug dependence who were formerly abstinent were purposively recruited from a compulsory drug treatment center	18	Qualitative	Not specified	Participants reported that, even during periods of abstinence, they perceived stigma from the community, including family and healthcare service providers; participants also reported feelings of shame; many reported social exclusion and difficulty finding employment Participants reported that stigma resulted in low treatment seeking and may have contributed to relapse	None
Zhang, 2016 [32]	China	Persons living with HIV were randomly selected for participation from a parent study	2987	Cross-sectional	Perceived, experienced, and internalized HIV stigma (Berger scale)	In overall sample, perceived stigma was associated with drug use; among those with higher incomes, internalized stigma was associated with drug use and experienced stigma was associated with alcohol use Perceived stigma was associated with drug use in rural areas	*Mod:* Relationship between stigma and drug use modified by income; odds of alcohol and drug use were highest among those with both higher levels of stigma and higher income; also modified by place of residence Those with higher levels of perceived stigma living in rural areas had increased odds of drug use compared to urban areas

*ART* antiretroviral therapy, *FGD* focus group discussion, *MMT* methadone maintenance therapy, *MSM* men who have sex with men, *NSP* needle and syringe programs, *PMTCT* prevention of mother-to-child transmission, *PWID* persons with injection drug use, *RCT* randomized controlled trial, *SES* socioeconomic status

### HIV

Among people with HIV, both internalized and experienced stigma have been associated with increased prevalence of HIV-related symptoms and poorer self-reported health [23–26] (Table 1). Internalized and experienced HIV-related stigma have been associated with increased prevalence of mental health disorders [27–29], particularly depression [30–40] and anxiety [41, 42]. For example, among Nigerians with HIV [43], stigma was associated with a diagnosis of severe depression, although it was not associated with mild or moderately severe depression. Among Tanzanian youth [28] and South African adults [28, 36], post-traumatic stress disorder was also more common among those with high levels of internalized stigma. All forms of stigma have been associated with decreased resilience and self-esteem among Chinese adults [32].

HIV-related stigma has been linked to poor health behaviors. Anticipated, experienced, and internalized stigma have been repeatedly associated with decreased voluntary HIV testing and disclosure of infection [24, 44–49]. For example, among Tanzanian adults obtaining HIV services [50], internalized stigma has been linked to increased denial of HIV infection. HIV-positive individuals who report experienced (enacted) stigma are more likely to delay initiation or continuation of HIV care [25, 51, 52]. Those who experience stigma in a healthcare setting are also less likely to initiate antiretroviral therapy [25]. Available cohort data suggests that perceived stigma is associated with poor medication adherence according to participant reports and chart reviews [53–55]. A longitudinal cohort study of adults living with HIV in South Africa revealed that internalized stigma was associated with a greater incidence of condomless sex with both HIV-negative/unknown and HIV-positive partners [5]. Finally, stigma has been associated with increases in smoking, alcohol, and drug use [32], as well as with suicidal ideation and attempted suicide [56, 57].

Significant mediators of the relationships between HIV-related stigma and health outcomes included individual resilience [23], depression, negative condom use attitudes [5], and self-efficacy [58]. While most data demonstrated an inverse relationship between quality of life and HIV-related stigma [59–61], this relationship may be mediated by depression [33, 38] and self-efficacy [58]. The association between HIV stigma and depression has been shown to be moderated by individual affect, social support, socioeconomic status, employment status, rural versus urban residence, and disclosure avoidance [32, 35, 62].

### Tuberculosis

TB-related stigma negatively impacts health outcomes by impeding healthcare seeking behavior, care delivery, and recovery (Table 2). Qualitative and quantitative studies have generally shown that stigma delays healthcare seeking, although a recent quantitative study did not find a strong deterrent effect of TB-related stigma when major drivers of healthcare seeking were included in a model [63]. Additionally, TB-related stigma can temporarily diminish social capital during treatment [64], and damage to family reputation can impact employment, education, and the marriage prospects of its members [65, 66].

Secondary stigma may manifest as a reluctance to expedite emergency care for acutely ill family members due to fear of disease disclosure to the broader community [66]. In communities where social capital functions as the safety net, loss of social status can imperil family survival [66, 67]. TB-related stigma was shown to damage the support networks and quality of services given to those who have a stigmatized condition [66]. Mistreatment of TB patients can contribute to mental health sequelae, poor coping behaviors, and other comorbidities [68, 69]. TB-related stigma may also erode patients’ resilience to disease and household-level wellbeing [70]. Finally, healthcare workers who perceive TB stigma defer TB screening and prophylaxis [71].

Studies have suggested that the impact of stigma on TB treatment adherence varies [72–74], with some suggesting a decrease [75] and others an increase [76, 77] in adherence. The predominance of cross-sectional data limits the ability to tease apart this relationship. Much of this variance can also likely be attributable to the diversity across studies with regards to measurement metrics and statistical power [78].

It is likely that drug-resistant TB (DRTB) has a different impact on the association of TB-related stigma with outcomes compared to drug-susceptible TB [79, 80]. DRTB disease may be more susceptible to blame, shame, and self-stigma because healthcare workers often assume it is caused by non-adherence. Further, DRTB treatment side effects can expose DRTB patients to mental health, disability, and poverty stigmas [81]. Stigma fed by perceived dangerousness and isolation policies that erode social capital and resilience may disproportionately affect people with DRTB. DRTB-related stigma may also be considered more of a barrier to adherence than HIV stigma among co-morbid persons [79, 82].

TB-related stigma can be exacerbated or attenuated by other forms of prejudice, including misogyny [83, 84]. Studies of TB-related stigma have also drawn attention to the moderating role of gender. Two studies found women were more adherent to TB treatment when they perceived high levels of stigma, while men were less so, particularly if they found TB treatment humiliating [65, 77]. There is also evident variation in the health impacts of TB-related stigma among sub-populations (e.g., people who inject drugs, alcohol dependent, pastoralists) [85–87].

### Mental health

Studies have indicated that mental health-related stigma is negatively associated with quality of life, functioning, and other positive health outcomes (Table 3). Quality of life was associated with either internalized or perceived stigma [89] and general functioning was inversely associated with internalized and perceived stigma [88, 89]. Greater stigma was also associated with fatigue [90] and poorer diabetes-related outcomes [91] among those with depression, and with HIV-risk behaviors among those with a severe mental illness [92].

Treatment outcomes were a major area of focus within the mental health articles identified. For example, studies on treatment adherence found internalized stigma to be associated with poorer medication adherence both among those with schizophrenia [93] and among those with any diagnosis of a mental disorder [94]. Perceived discrimination was also associated with higher odds of discontinuing medication among individuals diagnosed with schizophrenia [95]. Data on treatment-seeking behaviors for mental health problems were mixed. For example, one study found that individuals identified as having depression yet rejected treatment were more likely to have higher internalized stigma relative to those who accepted treatment [91]. In a community-based study from Ukraine [96], only 8% of individuals who were identified as having a mental health problem but not having sought help from any medical source cited stigma as a reason. However, nearly 75% of individuals living with severe mental illness in India reported delaying seeking care in part due to fear of stigma [97].

Symptom severity was the most common health-related outcome tested for associations with stigma; however, findings on the impacts of mental health-related stigma on mental disorder symptom severity are mixed. For example, two studies found that, among those diagnosed with schizophrenia, those with higher levels of internalized stigma had greater general psychiatric symptoms [88, 98]. In contrast, two studies found no relationship between general symptoms of psychopathology and most forms of internalized stigma assessed [89, 99], and one study found fewer experiences of stigma among those with more severe general psychiatric symptoms who were living with a severe mental illness [92]. Findings related to symptoms specific to schizophrenia were similarly mixed. Negative schizophrenia symptoms were not significantly associated with most forms of internalized stigma [35, 89, 99, 100]. Of three studies examining positive symptoms of schizophrenia [99–101], two found significant associations with stigma operating in opposite directions [99, 101]. For individuals with depression, greater symptom severity was associated with greater perceived stigma [90, 102–105], though one study found less stigma among those with higher levels of symptoms [99].

Moderators were assessed in only two studies on mental health-related stigma and health outcomes and no studies assessed mediators. In Jordan [106], depression was a moderator of the relationship between stigma and treatment seeking. Adolescents with mild depression who reported high levels of stigma were more likely to seek care from a variety of sources (counselor, general practitioner, religious leader, or family member) and express willingness to take medication or receive therapy than adolescents with mild depression who reported low levels of stigma. While moderate to severe depression was associated with lower likelihood of seeking care overall, there was no association between stigma and care-seeking for adolescents with moderate to severe depression. In Egypt [107], insight did not significantly modify the relationship between stigma and suicide risk among persons with schizophrenia.

### Epilepsy

Epilepsy-related stigma has been repeatedly linked to poor quality of life and associated with increased epilepsy-related concerns [108, 109], poor self-esteem [110], and increased self-reported burden of disease [111], including increased psychiatric burden such as that attributed to anxiety and depression [111–113] (Table 4). Qualitative and quantitative data suggest that epilepsy-related stigma leads to poor overall function, particularly regarding social engagement and employment [111, 114, 115]. Further, increased stigma has been associated with decreased disclosure and discussion about epilepsy [116, 117]. For example, 34% of married Pakistani women with epilepsy actively concealed their epilepsy diagnosis during marriage negotiations in response to misconceptions regarding their diagnosis, pressure from family members, and to avoid rejection and further stigmatization [118].

Epilepsy-associated stigma has also been shown to affect family members of people with epilepsy. Among mothers of children younger than 8 years with epilepsy, stigma has been associated with increased maternal psychopathology [119]. Mothers were also more likely to actively limit their child’s activities based on their own and perceived interpretation of their child’s internalized stigma [119].

Epilepsy-related stigma has been associated with social withdrawal and adverse health behaviors such as poor medication adherence [120]. This relationship may be mediated by increased medication side-effects reported among adults with epilepsy [121] as these side-effects have previously been associated with increased stigma [122, 123], though this relationship has yet to be formally examined as none of the epilepsy studies included in the review evaluated mediators or moderators.

### Substance use

Stigma is often prevalent among persons who use alcohol or other substances in both the community and in healthcare settings, with possible adverse consequences (Table 5). Among persons using alcohol or other substances, substance-related stigma was identified as a barrier to accessing drug treatment services [124, 125], general healthcare services [124, 126], HIV testing [127, 128], reduced antiretroviral therapy or treatment adherence [129–131], needle exchange programs [132], and to recovery generally [133]. Stigma among persons who use substances was also associated with less education and not being employed full-time [134], as well as lower quality of life across several domains, including the social, physical, psychological, and environmental domains [135], higher risk of relapse [136], social isolation, anxiety, and depression [137]. Healthcare professionals and trainees, including pharmacists and pharmacy students [138], medical students and recent medical graduates [139], and primary healthcare workers [140], expressed stigmatizing beliefs and attitudes towards persons who use drugs.

In addition to stigma occurring as a result of substance use, stigma related to HIV and other health conditions can also be associated with an increased risk for alcohol and other substance misuse. HIV stigma was associated with hazardous/harmful alcohol use among persons co-infected with HIV and TB [141]. Further, among persons with HIV and alcohol use, high levels of HIV-related stigma were associated with increased odds of experiencing physical and sexual violence [142]. Higher levels of HIV stigma were also associated with other (non-alcohol) substance use [143]. Stigma not attached to a health condition can also increase the risk of alcohol and other substance use; indeed, recent discrimination (e.g., based on race, age) was associated with increased odds of both alcohol and drug use [144–146].

Two studies investigated moderators. Years of study at university [145], income, and place of residence were found to be significant moderators of stigma–substance use relationships. One study investigated mediators and found that, among men who have sex with men, alcohol and substance use mediated the relationship between stigma and risky sexual behaviors [147].

### Intersectional stigmas

Stigmatized medical co-morbidities were common across the five conditions. In many LMICs, the prevalence of HIV and TB can be high and the burden of chronic non-infectious disorders like epilepsy, mental illness, and substance use is growing. HIV-related stigma has been associated with harmful alcohol use among individuals with comorbid HIV and TB infection [87] as well as increased (non-alcohol) substance use among individuals with HIV (alone) compared to those without HIV [143]. Stigma due to other marginalized characteristics (sex, race, gender, country of origin, etc.) also increases the risk of substance use and physical and sexual violence [142, 144–146]. This interaction has culminated in a syndemic, with an increased burden of stigma [148].

HIV-related stigma has been shown to attenuate the impact of TB-related stigma in some settings [77, 149], while potentiating it in others [150]. Comorbid stigmas do not always result in worse health outcomes. For example, while Zambian adults with HIV and epilepsy endorsed greater stigma, this did not translate into an increased prevalence of depression [151]. However, stigma due to one medical condition, such as substance use, has also been shown to hinder preventative care, including HIV testing [127, 128] and, among individuals with comorbid HIV infection, medication adherence [129–131].

## Discussion

Across disease types included in this scoping review, stigma was associated with poor individual health outcomes and health utilization patterns. Stigmas related to HIV, TB, epilepsy, and substance use were associated with increased psychiatric morbidity, particularly depression and anxiety. Stigma has repeatedly been associated with decreased quality of life and poorer functioning across conditions. Highly stigmatized individuals are more likely to conceal their condition and, as a result, are less likely to seek care or more likely to delay care. This is consistent with the literature from high-income countries on stigma related to mental health conditions. In a systematic review of 144 studies (the vast majority of which were from high income countries) [152], the median effect size of stigma on help-seeking for mental health disorder was -0.27, though there was some evidence that this relationship was stronger among ethnic minority groups within these countries; qualitative studies suggested that this is both a direct relationship and may be mediated through decreasing disclosure.

Among those obtaining treatment for all five conditions examined in this review, stigma was associated with decreased medication adherence and, among patients with substance use, relapse. In high-income countries, the relationships between treatment adherence and stigma related to mental health problems is varied. Perceived stigma has been found to predict poorer treatment outcomes for individuals with depression [153, 154]; though this evidence is mixed, internalized stigma has also been found to be related to poorer treatment adherence for individuals living with multiple mental health conditions [155]. Conversely, anticipated and experienced discrimination has been found to not be significantly associated with antipsychotic medication adherence for people living with schizophrenia [156].

Studies examining individuals with multiple stigmatized conditions suggest that the effects of health-related stigma can be felt across all domains. Just as stigma among individuals with mental health problems or injection drug use decreases their use of mental health and substance disorder services, it also decreases HIV testing and medication adherence. Unfortunately, the effects of stigma across conditions are complex and, similar to studies describing stigma in high-income countries [1], our review found that studies of stigma and health outcomes in LMICs are largely focused on one stigmatizing condition (often only internalized stigma) and one health outcome. Few studies described the complex interactions between different types of stigma and the co-occurring health conditions likely to be present.

As highlighted in Boxes 1, 2 and 3, the review results showed that marginalized members of society are increasingly vulnerable to health-related stigma. LGBTQ individuals, racial and ethnic minorities, and refugees suffer from increased stigma due to lack of social and economic stability, fear of encountering stigma, and increased self-stigmatization. Unequal access to treatment and, among refugee communities, decreased access to information, result in reduced healthcare-seeking behaviors. These associations can be amplified by perceived stigma from the healthcare community, which further delays care and reduces healthcare-seeking behavior. The effects of trauma, particularly among LGBTQ individuals and refugees, are often under-recognized, which also affects care. Unfortunately, as most studies recruit participants from healthcare settings, these individuals may have been overlooked within the available stigma data, and particularly in that related to HIV, mental health, and epilepsy. Similarly, difficulty in recruiting these populations presents a research challenge and affects data availability. Therefore, the effect of stigma on the health and health outcomes of vulnerable populations may be underestimated. While the substance use literature featured a wider range of populations, including representation of sexual and gender minorities, as well as geographies, the generalizability of this data is limited by its focus on alcohol and injection drug use; other substance types (e.g., inhalants, cocaine, prescription drugs) that may have associations with stigma have been largely neglected. Further, the effect of stigma on child and adolescent populations is poorly understood as only one study examining epilepsy-associated stigma focused on this vulnerable population [107]. Given that risk factors, symptom presentation, and trajectories of mental health and substance use problems may vary across the life course, increased research on stigma among children and adolescents is essential.

Comprehensive, multidisciplinary stigma-focused prevention and treatment approaches are warranted in LMICs. However, the design and implementation of these interventions is limited by the data available. This review highlights the paucity of longitudinal stigma studies on health-related stigma in LMICs, particularly among community-based samples, which limits our understanding of the mechanisms by which stigma impacts health outcomes. Appropriately designed quantitative cohort studies are vital to addressing these issues. Further, most of the studies included in this review were limited by small sample size and, as a result, data regarding mediators of the association between stigma and health outcomes is scant. Future research should include larger sample sizes that would enable more complex path modelling, including effect modification analysis. Available data suggests that gender is a moderator of both TB-related and substance use stigma. Understanding the effects of moderators and mediators on the relationship of stigma with individual health outcomes will improve the effectiveness of stigma reduction interventions.

### Limitations of the review

The purpose of the review was to inform both potential future research studies and possible research questions that could be addressed by systematic reviews. Formal study inclusion and exclusion criteria were not used as the review was not systematic; however, similar search terms and databases were used across the five disease reviews. Although the types of study designs described in the literature were often noted, individual study quality was not assessed, as is typical in scoping reviews. Finally, we focused on five disease/disorders that significantly drive the disease burden in LMICs. Future reviews should focus on other stigmatized conditions affecting individuals in this setting, including abortion, cancer, leprosy, albinism, gender identity, sex work, sexual violence, and sexually transmitted infections.

## Conclusion

A rapidly growing body of literature, mostly qualitative and cross-sectional in design, suggests that stigma is associated with poor health outcomes, including less help-seeking, among persons with HIV, TB, mental health, neurologic disorders, and substance use. This review highlights consistencies in the relationship of stigma with health outcomes, but also common methodological limitations. Future studies can address these limitations by (1) recognizing that comorbidity is the rule and not the exception and that the complex interconnected relationships between stigma and multiple health outcomes must be accounted for in the study design phase; (2) measuring multiple types of stigma at multiple health outcome levels; and (3) featuring longitudinal designs, investigation into mediators and moderators, and community-based study samples to improve generalizability. Removing the siloes from health-related stigma research in LMICs and addressing these limitations will improve the epidemiological literature on evidence-based stigma interventions, ultimately improving outcomes associated with high-burden diseases.

Box 1. Population of concern: LGBTQ A study done among transgender female sex workers in China reveals limited access to services due to amplified stigma because of their gender identity and their profession [230]. Thus, many decide to engage in self-medication, especially for the transitioning phase, including self-administering hormone use. A case study exploring the economic costs of stigma in India indicates different reasons; if discussing LGBTQ, it is the fear of family deprecation, professional discrimination, and overall societal rejection, yet healthcare providers confidentiality can also lead to discrimination, ultimately leading to breach of human rights [231]. All of them could potentially lead to adverse sexual health outcomes, suicide, and depression. A study performed in Vietnam [232], as a part of a case study series on researching LGBTQ in Asia, found that due to the stigma around the transgender society, transgender people end up doing their own research on gender-confirmation surgery or self-inject cheap and impure chemicals such as silicone and other petroleum products, which in some cases lead to serious harm and even fatalities. The same case study series, with findings from Nepal [233], reported that LGBTQ encounter stigma on daily basis from an early age, shaping how they perceive and interact with all aspects of society, including healthcare. The vast number of institutions, including those in Nepal, stigmatize gender and sexual minorities, with important implications for the ability of healthcare providers and institutions to address their health needs [233].^.^

Box 2. Population of concern: Racial and ethnic minorities Qualitative studies with refugee, asylum seeking, and immigrant new mothers [234] with depressive symptoms seeking mental health services, including a study with Korean American immigrant women [235], showed a challenging path to recovery due to social isolation and perceived stigma. A study that explored depression and care among Asian Indians in the USA collected data from interviews of 23 multidisciplinary mental health professionals and retrospective review of 20 medical records of patients [236]. Findings revealed that that social stigma contributed to the prolonged denial of a condition, difficulty in communicating the problem, and delayed professional intervention in those suffering from depression. People living with HIV are stigmatized and looked at negatively, with the fear of discrimination preventing patients from accessing care and the stigma remains a barrier to effectively addressing the disease [237]. Immigrant HIV-positive Latina women in the Midwest USA experienced feelings of stigma, leading to depression, rejection, or suicidal attempts; few had received any type of mental health care intervention [238]. Few cases of self-imposed stigma or ‘self-stigma’ as a result of minority status has led to reduced health-seeking behavior from health professionals due to fear that disclosing the minority status might be an obstacle from receiving care [239]. Similar findings were presented as part of a qualitative study in mental health among Asian communities in Australia and the unwillingness to access help from healthcare services due to stigma and shame [240]. Immigration and transmission of tuberculosis were reinforcing each other’s stigma [241].

Box 3. Population of concern: Refugees Tibetan refugees in Nepal faced different layers of barriers, behavioral norms, and institutional structures that impair the diffusion of relevant information, creating a challenge to develop a comprehensive understanding of HIV/AIDS [242]. The stigma in both host and their own societies was hindering the individual’s willingness to discuss the issue with their peers and with medical professionals [242]. A case study based on a literature review and semi-structured interviews of urban refugees in Egypt at high risk for HIV/AIDS [243] revealed that intense stigma and discrimination, vulnerability, and social stability resulted in a lack of adequate health resources and a chain of causation that marginalized refugees in Egyptian society. These social processes result in unequal access to health resources, thereby increasing their potential exposure to HIV transmission. The sexual violence being used as a weapon of war during conflicts (conflict-related sexual violence) has caused significant trauma in both women and men survivors. The experience of refugees in Ethiopia shows that the stigma associated with conflict-related sexual violence makes it challenging for the survivors to mitigate the potential long-term physical, mental, reproductive health, and social consequences [244].

## Additional file

Additional file 1: Full list of search terms for each database searched. (DOCX 39 kb)

## Abbreviations

DRTB: drug-resistant tuberculosis
LGBTQ: lesbian, gay, bisexual, transgender, queer
LMICs: low- and middle-income countries
TB: tuberculosis
