# Supplementary material for: A scoping review of health-related stigma outcomes for high-burden diseases in low- and middle-income countries
Source: BMC Med. 2019 Feb 15;17:17. doi: 10.1186/s12916-019-1250-8 (PMC6376728; doi:10.1186/s12916-019-1250-8)
Supplement: Supplementary file 1 — Full list of search terms for each database searched. (DOCX 39 kb) [file 12916_2019_1250_MOESM1_ESM.docx]

**HIV/AIDS**

PubMed

((((HIV[Title/Abstract]) OR AIDS[Title/Abstract] AND

(Afghanistan[Title/Abstract] OR Albania[Title/Abstract] OR Algeria[Title/Abstract] OR Samoa[Title/Abstract] OR Angola[Title/Abstract] OR Antigua[Title/Abstract] OR Barbuda[Title/Abstract] OR Argentina[Title/Abstract] OR Armenia[Title/Abstract] OR Azerbaijan[Title/Abstract] OR Bangladesh[Title/Abstract] OR Belarus[Title/Abstract] OR Belize[Title/Abstract] OR Benin[Title/Abstract] OR Bhutan[Title/Abstract] OR Bolivia[Title/Abstract] OR Bosnia[Title/Abstract] OR Herzegovina[Title/Abstract] OR Botswana[Title/Abstract] OR Brazil[Title/Abstract] OR Bulgaria[Title/Abstract] OR Burkina Faso[Title/Abstract] OR Burundi[Title/Abstract] OR Cambodia[Title/Abstract] OR Cameroon[Title/Abstract] OR Cabo Verde[Title/Abstract] OR Central African Republic[Title/Abstract] OR Chad[Title/Abstract] OR Chile[Title/Abstract] OR China[Title/Abstract] OR Colombia[Title/Abstract] OR Comoros[Title/Abstract] OR Congo[Title/Abstract] OR Costa Rica[Title/Abstract] OR Côte d'Ivoire[Title/Abstract] OR Cote d’Ivoire[Title/Abstract] OR Ivory[Title/Abstract] OR Cuba[Title/Abstract] OR Djibouti[Title/Abstract] OR Dominica[Title/Abstract] OR Dominican[Title/Abstract] OR Ecuador[Title/Abstract] OR Egypt[Title/Abstract] OR Salvador[Title/Abstract] OR Eritrea[Title/Abstract] OR Ethiopia[Title/Abstract] OR Fiji[Title/Abstract] OR Gabon[Title/Abstract] OR Gambia[Title/Abstract] OR Georgia[Title/Abstract] OR Ghana[Title/Abstract] OR Grenada[Title/Abstract] OR Guatemala[Title/Abstract] OR Guinea[Title/Abstract] OR Guinea-Bissau[Title/Abstract] OR Guyana[Title/Abstract] OR Haiti[Title/Abstract] OR Honduras[Title/Abstract] OR India[Title/Abstract] OR Indonesia[Title/Abstract] OR Iran[Title/Abstract] OR Iraq[Title/Abstract] OR Jamaica[Title/Abstract] OR Jordan[Title/Abstract] OR Kazakhstan[Title/Abstract] OR Kenya[Title/Abstract] OR Kiribati[Title/Abstract] OR Korea [Title/Abstract] OR Kosovo[Title/Abstract] OR Kyrgyz [Title/Abstract] OR Lao[Title/Abstract] OR Laos[Title/Abstract] OR Latvia[Title/Abstract] OR Lebanon[Title/Abstract] OR Lesotho[Title/Abstract] OR Liberia[Title/Abstract] OR Libya[Title/Abstract] OR Lithuania[Title/Abstract] OR Macedonia[Title/Abstract] OR Madagascar[Title/Abstract] OR Malawi[Title/Abstract] OR Malaysia[Title/Abstract] OR Maldives[Title/Abstract] OR Mali[Title/Abstract] OR Marshall[Title/Abstract] OR Mauritania[Title/Abstract] OR Mauritius[Title/Abstract] OR Mexico[Title/Abstract] OR Micronesia[Title/Abstract] OR Moldova[Title/Abstract] OR Mongolia[Title/Abstract] OR Montenegro[Title/Abstract] OR Morocco[Title/Abstract] OR Mozambique[Title/Abstract] OR Myanmar[Title/Abstract] OR Namibia[Title/Abstract] OR Nepal[Title/Abstract] OR Nicaragua[Title/Abstract] OR Niger[Title/Abstract] OR Nigeria[Title/Abstract] OR Pakistan[Title/Abstract] OR Palau[Title/Abstract] OR Panama[Title/Abstract] OR Papua New Guinea[Title/Abstract] OR Paraguay[Title/Abstract] OR Peru[Title/Abstract] OR Philippines[Title/Abstract] OR Romania[Title/Abstract] OR Russia[Title/Abstract] OR Russian[Title/Abstract] OR Rwanda[Title/Abstract] OR Samoa[Title/Abstract] OR Sao Tome[Title/Abstract] OR Senegal[Title/Abstract] OR Serbia[Title/Abstract] OR Seychelles[Title/Abstract] OR Sierra Leone[Title/Abstract] OR Solomon Islands[Title/Abstract] OR Somalia[Title/Abstract] OR South Africa[Title/Abstract] OR Sri Lanka[Title/Abstract] OR St. Lucia[Title/Abstract] OR St. Vincent[Title/Abstract] OR Grenadines[Title/Abstract] OR Sudan[Title/Abstract] OR Suriname[Title/Abstract] OR Swaziland[Title/Abstract] OR Syrian[Title/Abstract] OR Syria[Title/Abstract] OR Tajikistan[Title/Abstract] OR Tanzania[Title/Abstract] OR Thailand[Title/Abstract] OR Timor-Leste[Title/Abstract] OR Togo[Title/Abstract] OR Tonga[Title/Abstract] OR Tunisia[Title/Abstract] OR Turkey[Title/Abstract] OR Turkmenistan[Title/Abstract] OR Tuvalu[Title/Abstract] OR Uganda[Title/Abstract] OR Ukraine[Title/Abstract] OR Uruguay[Title/Abstract] OR Uzbekistan[Title/Abstract] OR Vanuatu[Title/Abstract] OR Venezuela[Title/Abstract] OR Vietnam[Title/Abstract] OR “West Bank”[Title/Abstract] OR Gaza[Title/Abstract] OR Yemen[Title/Abstract] OR Zambia[Title/Abstract] OR Zimbabwe [Title/Abstract] OR “developing countries”[Title/Abstract] OR “resource-limited”[Title/Abstract] OR “resource-constrained”[Title/Abstract] OR “low- and middle-income”[Title/Abstract] OR LMIC[Title/Abstract] OR “third world”[Title/Abstract] OR “low income countries”[Title/Abstract])))

AND ("2008"[Date - Publication] : "3000"[Date - Publication])) AND

((stigma[Title/Abstract] OR stigmatize[Title/Abstract] OR stigmatise[Title/Abstract] OR stigmatizing[Title/Abstract] OR stigmatising[Title/Abstract] OR stereotype[Title/Abstract] OR stereotyping[Title/Abstract] OR shame[Title/Abstract] OR shaming[Title/Abstract] OR discrimination[Title/Abstract] OR discriminating[Title/Abstract] OR “social distance”[Title/Abstract] OR prejudice[Title/Abstract] OR blame[Title/Abstract]))

PsychInfo

(TX(HIV) OR TX(AIDS)) AND

(TX(Afghanistan) OR TX(Albania) OR TX(Algeria) OR TX(Samoa) OR TX(Angola) OR TX(Antigua) OR TX(Barbuda) OR TX(Argentina) OR TX(Armenia) OR TX(Azerbaijan) OR TX(Bangladesh) OR TX(Belarus) OR TX(Belize) OR TX(Benin) OR TX(Bhutan) OR TX(Bolivia) OR TX(Bosnia) OR TX(Herzegovina) OR TX(Botswana) OR TX(Brazil) OR TX(Bulgaria) OR TX(Burkina Faso) OR TX(Burundi) OR TX(Cambodia) OR TX(Cameroon) OR TX(Cabo Verde) OR TX(Central African Republic) OR TX(Chad) OR TX(Chile) OR TX(China) OR TX(Colombia) OR TX(Comoros) OR TX(Congo) OR TX(Costa Rica) OR TX(Côte d'Ivoire) OR TX(Cote d’Ivoire) OR TX(Ivory) OR TX(Cuba) OR TX(Djibouti) OR TX(Dominica) OR TX(Dominican) OR TX(Ecuador) OR OR TX(Egypt) OR TX(Salvador) OR TX(Eritrea) OR TX(Ethiopia) OR TX(Fiji) OR TX(Gabon) OR TX(Gambia) OR TX(Georgia) OR TX(Ghana) OR TX(Grenada) OR TX(Guatemala) OR TX(Guinea) OR TX(Guinea-Bissau) OR TX(Guyana) OR TX(Haiti) OR TX(Honduras) OR TX(India) OR TX(Indonesia) OR TX(Iran) OR TX(Iraq) OR TX(Jamaica) OR TX(Jordan) OR TX(Kazakhstan) OR TX(Kenya) OR TX(Kiribati) OR TX(Korea) OR TX(Kosovo) OR TX(Kyrgyz) OR TX(Lao) OR TX(Laos) OR TX(Latvia) OR TX(Lebanon) OR TX(Lesotho) OR TX(Liberia) OR TX(Libya) OR TX(Lithuania) OR TX(Macedonia) OR TX(Madagascar) OR TX(Malawi) OR TX(Malaysia) OR TX(Maldives) OR TX(Mali) OR TX(Marshall) OR TX(Mauritania) OR TX(Mauritius) OR TX(Mexico) OR TX(Micronesia) OR TX(Moldova) OR TX(Mongolia) OR TX(Montenegro) OR TX(Morocco) OR TX(Mozambique) OR TX(Myanmar) OR TX(Namibia) OR TX(Nepal) OR TX(Nicaragua) OR TX(Niger) OR TX(Nigeria) OR TX(Pakistan) OR TX(Palau) OR TX(Panama) OR TX(Papua New Guinea) OR TX(Paraguay) OR TX(Peru) OR TX(Philippines) OR TX(Romania) OR TX(Russia) OR TX(Russian) OR TX(Rwanda) OR TX(Samoa) OR TX(Sao Tome) OR TX(Senegal) OR TX(Serbia) OR TX(Seychelles) OR TX(Sierra Leone) OR TX(Solomon Islands) OR TX(Somalia) OR TX(South Africa) OR TX(Sri Lanka) OR TX(St. Lucia) OR TX(St. Vincent) OR TX(Grenadines) OR TX(Sudan) OR TX(Suriname) OR TX(Swaziland) OR TX(Syrian) OR TX(Syria) OR TX(Tajikistan) OR TX(Tanzania) OR TX(Thailand) OR TX(Timor-Leste) OR TX(Togo) OR TX(Tonga) OR TX(Tunisia) OR TX(Turkey) OR TX(Turkmenistan) OR TX(Tuvalu) OR TX(Uganda) OR TX(Ukraine) OR TX(Uruguay) OR TX(Uzbekistan) OR TX(Vanuatu) OR TX(Venezuela) OR TX(Vietnam) OR TX(West Bank) OR TX(Gaza) OR TX(Yemen) OR TX(Zambia) OR TX(Zimbabwe) OR TX(developing countries) OR TX(resource-limited) OR TX(resource-constrained) OR TX(low- and middle-income) OR TX(LMIC) OR TX(third world) OR TX(low income countries))

AND

(TX(stigma) OR TX(stigmatize) OR TX(stigmatise) OR (TX)stigmatizing OR TX(stigmatizing) OR TX(stereotype) OR TX(stereotyping) OR TX(shame) OR TX(shaming) OR TX(discrimination) OR TX(discriminating) OR TX(social distance) OR TX(prejudice) OR TX(blame))

EMBASE

(HIV:ti,ab OR AIDS:ti,ab) AND

('developing country':ti,ab OR 'developing countries':ti,ab OR 'developing nation':ti,ab OR 'developing nations':ti,ab OR 'developing population':ti,ab OR 'developing populations':ti,ab OR 'developing world':ti,ab OR 'less developed country':ti,ab OR 'less developed countries':ti,ab OR 'less developed nation':ti,ab OR 'less developed nations':ti,ab OR 'less developed population':ti,ab OR 'less developed populations':ti,ab OR 'less developed world':ti,ab OR 'lesser developed country':ti,ab OR 'lesser developed countries':ti,ab OR 'lesser developed nation':ti,ab OR 'lesser developed nations':ti,ab OR 'lesser developed population':ti,ab OR 'lesser developed populations':ti,ab OR 'lesser developed world':ti,ab OR 'under developed country':ti,ab OR 'under developed countries':ti,ab OR 'under developed nation':ti,ab OR 'under developed nations':ti,ab OR 'under developed population':ti,ab OR 'under developed populations':ti,ab OR 'under developed world':ti,ab OR 'underdeveloped country':ti,ab OR 'underdeveloped countries':ti,ab OR 'underdeveloped nation':ti,ab OR 'underdeveloped nations':ti,ab OR 'underdeveloped population':ti,ab OR 'underdeveloped populations':ti,ab OR 'underdeveloped world':ti,ab OR 'middle income country':ti,ab OR 'middle income countries':ti,ab OR 'middle income nation':ti,ab OR 'middle income nations':ti,ab OR 'middle income population':ti,ab OR 'middle income populations':ti,ab OR 'low income country':ti,ab OR 'low income countries':ti,ab OR 'low income nation':ti,ab OR 'low income nations':ti,ab OR 'low income population':ti,ab OR 'low income populations':ti,ab OR 'lower income country':ti,ab OR 'lower income countries':ti,ab OR 'lower income nation':ti,ab OR 'lower income nations':ti,ab OR 'lower income population':ti,ab OR 'lower income populations':ti,ab OR 'underserved country':ti,ab OR 'underserved countries':ti,ab OR 'underserved nation':ti,ab OR 'underserved nations':ti,ab OR 'underserved population':ti,ab OR 'underserved populations':ti,ab OR 'underserved world':ti,ab OR 'under served country':ti,ab OR 'under served countries':ti,ab OR 'under served nation':ti,ab OR 'under served nations':ti,ab OR 'under served population':ti,ab OR 'under served populations':ti,ab OR 'under served world':ti,ab OR 'deprived country':ti,ab OR 'deprived countries':ti,ab OR 'deprived nation':ti,ab OR 'deprived nations':ti,ab OR 'deprived population':ti,ab OR 'deprived populations':ti,ab OR 'deprived world':ti,ab OR 'poor country':ti,ab OR 'poor countries':ti,ab OR 'poor nation':ti,ab OR 'poor nations':ti,ab OR 'poor population':ti,ab OR 'poor populations':ti,ab OR 'poor world':ti,ab OR 'poorer country':ti,ab OR 'poorer countries':ti,ab OR 'poorer nation':ti,ab OR 'poorer nations':ti,ab OR 'poorer population':ti,ab OR 'poorer populations':ti,ab OR 'poorer world':ti,ab OR 'developing economy':ti,ab OR 'developing economies':ti,ab OR 'less developed economy':ti,ab OR 'less developed economies':ti,ab OR 'lesser developed economy':ti,ab OR 'lesser developed economies':ti,ab OR 'under developed economy':ti,ab OR 'under developed economies':ti,ab OR 'underdeveloped economy':ti,ab OR 'underdeveloped economies':ti,ab OR 'middle income economy':ti,ab OR 'middle income economies':ti,ab OR 'low income economy':ti,ab OR 'low income economies':ti,ab OR 'lower income economy':ti,ab OR 'lower income economies':ti,ab OR 'low gdp':ti,ab OR 'low gnp':ti,ab OR 'low gross domestic':ti,ab OR 'low gross national':ti,ab OR 'lower gdp':ti,ab OR 'lower gnp':ti,ab OR 'lower gross domestic':ti,ab OR 'lower gross national':ti,ab OR lmic:ti,ab OR lmics:ti,ab OR 'third world':ti,ab OR 'lami country':ti,ab OR 'lami countries':ti,ab OR 'transitional country':ti,ab OR 'transitional countries':ti,ab OR africa:ti,ab OR asia:ti,ab OR caribbean:ti,ab OR 'west indies':ti,ab OR 'south america':ti,ab OR 'latin america':ti,ab OR 'central america':ti,ab OR 'atlantic islands':ti,ab OR 'commonwealth of independent states':ti,ab OR 'pacific islands':ti,ab OR 'indian ocean islands':ti,ab OR 'eastern europe':ti,ab OR afghanistan:ti,ab OR albania:ti,ab OR algeria:ti,ab OR angola:ti,ab OR antigua:ti,ab OR barbuda:ti,ab OR argentina:ti,ab OR armenia:ti,ab OR armenian:ti,ab OR aruba:ti,ab OR azerbaijan:ti,ab OR bahrain:ti,ab OR bangladesh:ti,ab OR barbados:ti,ab OR benin:ti,ab OR byelarus:ti,ab OR byelorussian:ti,ab OR belarus:ti,ab OR belorussian:ti,ab OR belorussia:ti,ab OR belize:ti,ab OR bhutan:ti,ab OR bolivia:ti,ab OR bosnia:ti,ab OR herzegovina:ti,ab OR hercegovina:ti,ab OR botswana:ti,ab OR brasil:ti,ab OR brazil:ti,ab OR bulgaria:ti,ab OR 'burkina faso':ti,ab OR 'burkina fasso':ti,ab OR 'upper volta':ti,ab OR burundi:ti,ab OR urundi:ti,ab OR cambodia:ti,ab OR 'khmer republic':ti,ab OR kampuchea:ti,ab OR cameroon:ti,ab OR cameroons:ti,ab OR cameron:ti,ab OR camerons:ti,ab OR cameroun:ti,ab OR 'cape verde':ti,ab OR 'central african republic':ti,ab OR chad:ti,ab OR chile:ti,ab OR china:ti,ab OR colombia:ti,ab OR comoros:ti,ab OR 'comoro islands':ti,ab OR comores:ti,ab OR mayotte:ti,ab OR congo:ti,ab OR zaire:ti,ab OR 'costa rica':ti,ab OR 'cote divoire':ti,ab OR 'ivory coast':ti,ab OR croatia:ti,ab OR cuba:ti,ab OR cyprus:ti,ab OR czechoslovakia:ti,ab OR 'czech republic':ti,ab OR slovakia:ti,ab OR 'slovak republic':ti,ab OR djibouti:ti,ab OR 'french somaliland':ti,ab OR dominica:ti,ab OR 'dominican republic':ti,ab OR 'east timor':ti,ab OR 'east timur':ti,ab OR 'timor leste':ti,ab OR ecuador:ti,ab OR egypt:ti,ab OR 'united arab republic':ti,ab OR 'el salvador':ti,ab OR eritrea:ti,ab OR estonia:ti,ab OR ethiopia:ti,ab OR fiji:ti,ab OR gabon:ti,ab OR 'gabonese republic':ti,ab OR gambia:ti,ab OR gaza:ti,ab OR 'georgia republic':ti,ab OR 'georgian republic':ti,ab OR ghana:ti,ab OR 'gold coast':ti,ab OR greece:ti,ab OR grenada:ti,ab OR guatemala:ti,ab OR guinea:ti,ab OR guam:ti,ab OR guiana:ti,ab OR guyana:ti,ab OR haiti:ti,ab OR honduras:ti,ab OR hungary:ti,ab OR india:ti,ab OR maldives:ti,ab OR indonesia:ti,ab OR iran:ti,ab OR iraq:ti,ab OR 'isle of man':ti,ab OR jamaica:ti,ab OR jordan:ti,ab OR kazakhstan:ti,ab OR kazakh:ti,ab OR kenya:ti,ab OR kiribati:ti,ab OR korea:ti,ab OR kosovo:ti,ab OR kyrgyzstan:ti,ab OR kirghizia:ti,ab OR 'kyrgyz republic':ti,ab OR kirghiz:ti,ab OR kirgizstan:ti,ab OR 'lao pdr':ti,ab OR laos:ti,ab OR latvia:ti,ab OR lebanon:ti,ab OR lesotho:ti,ab OR basutoland:ti,ab OR liberia:ti,ab OR libya:ti,ab OR lithuania:ti,ab OR macedonia:ti,ab OR madagascar:ti,ab OR 'malagasy republic':ti,ab OR malaysia:ti,ab OR malaya:ti,ab OR malay:ti,ab OR sabah:ti,ab OR sarawak:ti,ab OR malawi:ti,ab OR nyasaland:ti,ab OR mali:ti,ab OR malta:ti,ab OR 'marshall islands':ti,ab OR mauritania:ti,ab OR mauritius:ti,ab OR 'agalega islands':ti,ab OR 'melanesia':ti,ab OR mexico:ti,ab OR micronesia:ti,ab OR 'middle east':ti,ab OR moldova:ti,ab OR moldovia:ti,ab OR moldovian:ti,ab OR mongolia:ti,ab OR morocco:ti,ab OR mozambique:ti,ab OR myanmar:ti,ab OR myanma:ti,ab OR burma:ti,ab OR namibia:ti,ab OR nepal:ti,ab OR 'netherlands antilles':ti,ab OR 'new caledonia':ti,ab OR nicaragua:ti,ab OR niger:ti,ab OR nigeria:ti,ab OR 'northern mariana islands':ti,ab OR oman:ti,ab OR muscat:ti,ab OR pakistan:ti,ab OR palau:ti,ab OR palestine:ti,ab OR panama:ti,ab OR paraguay:ti,ab OR peru:ti,ab OR philippines:ti,ab OR philipines:ti,ab OR phillipines:ti,ab OR phillippines:ti,ab OR poland:ti,ab OR portugal:ti,ab OR 'puerto rico':ti,ab OR romania:ti,ab OR rumania:ti,ab OR roumania:ti,ab OR russia:ti,ab OR russian:ti,ab OR rwanda:ti,ab OR ruanda:ti,ab OR 'saint kitts':ti,ab OR 'st kitts':ti,ab OR nevis:ti,ab OR 'saint lucia':ti,ab OR 'st lucia':ti,ab OR 'saint vincent':ti,ab OR 'st vincent':ti,ab OR grenadines:ti,ab OR samoa:ti,ab OR 'samoan islands':ti,ab OR 'navigator island':ti,ab OR 'navigator islands':ti,ab OR 'sao tome':ti,ab OR 'saudi arabia':ti,ab OR senegal:ti,ab OR serbia:ti,ab OR montenegro:ti,ab OR seychelles:ti,ab OR 'sierra leone':ti,ab OR slovenia:ti,ab OR 'sri lanka':ti,ab OR ceylon:ti,ab OR 'solomon islands':ti,ab OR somalia:ti,ab OR sudan:ti,ab OR suriname:ti,ab OR surinam:ti,ab OR swaziland:ti,ab OR syria:ti,ab OR syrian:ti,ab OR tajikistan:ti,ab OR tadzhikistan:ti,ab OR tadjikistan:ti,ab OR tadzhik:ti,ab OR tanzania:ti,ab OR thailand:ti,ab OR togo:ti,ab OR 'togolese republic':ti,ab OR tonga:ti,ab OR trinidad:ti,ab OR tobago:ti,ab OR tunisia:ti,ab OR turkey:ti,ab OR turkmenistan:ti,ab OR turkmen:ti,ab OR tuvalu:ti,ab OR uganda:ti,ab OR ukraine:ti,ab OR uruguay:ti,ab OR ussr:ti,ab OR 'soviet union':ti,ab OR 'union of soviet socialist republics':ti,ab OR uzbekistan:ti,ab OR uzbek OR vanuatu:ti,ab OR 'new hebrides':ti,ab OR venezuela:ti,ab OR vietnam:ti,ab OR 'viet nam':ti,ab OR 'west bank':ti,ab OR yemen:ti,ab OR yugoslavia:ti,ab OR zambia:ti,ab OR zimbabwe:ti,ab OR rhodesia:ti,ab OR 'developing country'/exp OR 'africa'/de OR 'africa south of the sahara'/de OR 'central africa'/de OR 'asia'/de OR 'southeast asia'/de OR 'caribbean'/de OR 'caribbean islands'/de OR 'south america'/de OR 'south and central america'/de OR 'atlantic islands'/de OR 'ussr'/de OR 'pacific islands'/de OR 'indian ocean'/de OR 'eastern europe'/de OR 'afghanistan'/exp OR 'albania'/exp OR 'algeria'/exp OR 'american samoa'/exp OR 'angola'/exp OR 'antigua and barbuda'/exp OR 'argentina'/exp OR 'armenia'/exp OR 'azerbaijan'/exp OR 'bahrain'/exp OR 'baltic states'/exp OR 'bangladesh'/exp OR 'barbados'/exp OR 'benin'/exp OR 'belarus'/exp OR 'belize'/exp OR 'bhutan'/exp OR 'bolivia'/exp OR 'bosnia and herzegovina'/exp OR 'botswana'/exp OR 'brazil'/exp OR 'bulgaria'/exp OR 'burkina faso'/exp OR 'burundi'/exp OR 'cambodia'/exp OR 'cameroon'/exp OR 'cape verde' OR 'central african republic'/exp OR 'chad'/exp OR 'chile'/exp OR 'china'/exp OR 'colombia'/exp OR 'comoros'/exp OR 'congo'/exp OR 'costa rica'/exp OR 'cote divoire' OR 'croatia'/exp OR 'cuba'/exp OR 'cyprus'/exp OR 'czechoslovakia'/exp OR 'czech republic'/exp OR 'slovakia'/exp OR 'djibouti'/exp OR 'democratic republic congo'/exp OR 'north korea'/exp OR 'dominica'/exp OR 'dominican republic'/exp OR 'dominican (dominican republic)'/exp OR 'timor leste'/exp OR 'ecuador'/exp OR 'egypt'/exp OR 'el salvador'/exp OR 'eritrea'/exp OR 'estonia'/exp OR 'ethiopia'/exp OR 'equatorial guinea'/exp OR 'fiji'/exp OR 'french guiana'/exp OR 'gabon'/exp OR 'gambia'/exp OR 'georgia (republic)'/exp OR 'ghana'/exp OR 'greece'/exp OR 'grenada'/exp OR 'guatemala'/exp OR 'guinea'/exp OR 'guinea bissau'/exp OR 'guam'/exp OR 'guyana'/exp OR 'haiti'/exp OR 'honduras'/exp OR 'hungary'/exp OR 'samoa'/exp OR 'india'/exp OR 'indonesia'/exp OR 'iran'/exp OR 'iraq'/exp OR 'jamaica'/exp OR 'jordan'/exp OR 'kazakhstan'/exp OR 'kenya'/exp OR 'south korea'/exp OR 'kyrgyzstan'/exp OR 'laos'/exp OR 'latvia'/exp OR 'lebanon'/exp OR 'lesotho'/exp OR 'liberia'/exp OR 'libyan arab jamahiriya'/exp OR 'lithuania'/exp OR 'macedonia (republic)'/exp OR 'madagascar'/exp OR 'malawi'/exp OR 'malaysia'/exp OR 'mali'/exp OR 'malta'/exp OR 'mauritania'/exp OR 'mauritius'/exp OR 'melanesia'/exp OR 'mexico'/exp OR 'federated states of micronesia'/exp OR 'middle east'/de OR 'moldova'/exp OR 'mongolia'/exp OR 'morocco'/exp OR 'mozambique'/exp OR 'myanmar'/exp OR 'namibia'/exp OR 'nepal'/exp OR 'netherlands antilles'/exp OR 'new caledonia'/exp OR 'nicaragua'/exp OR 'niger'/exp OR 'nigeria'/exp OR 'oman'/exp OR 'pakistan'/exp OR 'palau'/exp OR 'panama'/exp OR 'papua new guinea'/exp OR 'paraguay'/exp OR 'peru'/exp OR 'philippines'/exp OR 'poland'/exp OR 'portugal'/exp OR 'puerto rico'/exp OR 'romania'/exp OR 'russian federation'/exp OR 'rwanda'/exp OR 'saint kitts and nevis'/exp OR 'saint lucia'/exp OR 'saint vincent and the grenadines'/exp OR 'saudi arabia'/exp OR 'senegal'/exp OR 'serbia'/exp OR 'montenegro (republic)'/exp OR 'seychelles'/exp OR 'sierra leone'/exp OR 'slovenia'/exp OR 'sri lanka'/exp OR 'somalia'/exp OR 'south africa'/exp OR 'sudan'/exp OR 'suriname'/exp OR 'swaziland'/exp OR 'syrian arab republic'/exp OR 'tajikistan'/exp OR 'tanzania'/exp OR 'thailand'/exp OR 'togo'/exp OR 'tonga'/exp OR 'trinidad and tobago'/exp OR 'tunisia'/exp OR 'turkey (republic)'/exp OR 'turkmenistan'/exp OR 'uganda'/exp OR 'ukraine'/exp OR 'uruguay'/exp OR 'uzbekistan'/exp OR 'vanuatu'/exp OR 'venezuela'/exp OR 'viet nam'/exp OR 'yemen'/exp OR 'yugoslavia'/exp OR 'yugoslavia (pre-1992)' OR 'zambia'/exp OR 'zimbabwe'/exp) AND

(stigma:ti,ab OR stigmatize:ti,ab OR stigmatise:ti,ab OR stigmatizing:ti,ab OR stigmatizing:ti,ab OR stereotype:ti,ab OR stereotyping:ti,ab OR shame:ti,ab OR shaming:ti,ab OR discrimination:ti,ab OR discriminating:ti,ab OR ‘social distance’:ti,ab OR prejudice:ti,ab OR blame:ti,ab)

AND

[2008-2017]/py

**TB**

PubMed

TB OR Tubercul* OR “pulmonary consumption” OR

AND

stigma* OR self-stigma* OR discrimination OR attitud* OR discredit* OR prejudic* OR stereotyp* OR marginaliz* OR “social isolation” OR “social exclusion” OR “social inclusion” OR “social distancing” OR “social distance” OR shame* OR guilt* OR fear* OR depress* OR “social support” OR “family support” OR psychosocial OR psycholog* OR disclos* OR “social rejection” OR “human rights” OR “patient rights” OR “negative belief” OR “negative opinion” OR ”hate” OR “dignity” OR “disclosure” OR “blame”

only STUDIES IN humans

NOT CONTAINING THESES WORDS IN TITLES:

1. deer
2. cattle
3. possum
4. macaque*
5. guinea pig*
6. animal
7. vaccine
8. BCG
9. mice
10. regimen
11. fixed-dose
12. side-effect*
13. survival
14. biopsy
15. interferon-gamma
16. pathophysiology
17. mortality
18. clinical + outcome*
19. meningitis
20. Treatment+ outcome*
21. genotyp*
22. bacille Calmette
23. Missing+data
24. re-vaccination
25. candidate
26. bovi*
27. non-tubercul*
28. strain
29. Case+ report
30. dose-response
31. adverse
32. phenotyp*

FURTHER EXCLUDE ARTICLES ON Subject Areas=

1. Agriculture,
2. Allergy
3. Anatomy & Morphology
4. Anesthesiology
5. Applied Radiology,
6. Biochemistry & Molecular Biology
7. Biology
8. Biophysics
9. Cardiac & Cardiovascular Systems
10. Cell Biology
11. Chemistry,
12. Chemistry, Medicinal
13. Chemistry, Organic
14. Dentistry,
15. Dermatology
16. Ecology
17. Endocrinology & Metabolism
18. Engineering, Biomedical
19. Environmental Sciences
20. Evolutionary Biology
21. Food Science & Technology
22. Gastroenterology & Hepatology
23. Genetics & Heredity
24. Geriatrics & Gerontology
25. Gerontology
26. Hematology
27. History & Philosophy Of Science
28. Immunology
29. Legal Mathematical & Computational Biology
30. Microbiology
31. Multidisciplinary
32. NephrologyNeuroimaging
33. Nuclear Medicine
34. Nutrition & Dietetics
35. Oncology
36. Ophthalmology
37. Oral Surgery & Medicine
38. Orthopedics
39. Otorhinolaryngology
40. Parasitology
41. Pathology
42. Pharmacology & Pharmacy
43. Physical
44. Rehabilitation
45. Rheumatology
46. Surgery
47. Toxicology
48. Urology
49. Veterinary Sciences
50. Virology
51. Zoology

PsychInfo

TB OR Tubercul* OR “pulmonary consumption” =3111 HITS

AND

stigma* OR self-stigma* OR discrimination OR attitud* OR discredit* OR prejudic* OR stereotyp* OR marginaliz* OR social isolation OR social exclusion OR social inclusion OR social distancing OR social distance OR shame* OR guilt* OR fear* OR depress* OR social support OR family support OR psychosocial OR psycholog* OR disclos* OR social rejection OR human rights OR patient rights OR negative belief OR negative opinion OR hate OR dignity OR disclosure OR blame

HITS= 1,413,949

JOIN= 912

No limits applied

WEB OF SCIENCE – HITS using original criteria ==8,295

I changed the second set of criteris from SU to TI and got HITS =566

CINDHAL

Applied original search critera—except exclude MEDLINE citations

HITS- 869

EMBASE

TB OR Tubercul* OR pulmonary consumption =34,000 HITS

stigma* OR self-stigma* OR discrimination OR attitud* OR discredit* OR prejudic* OR stereotyp* OR marginaliz* OR social isolation OR social exclusion OR social inclusion OR social distancing OR social distance OR shame* OR guilt* OR fear* OR depress* OR social support OR family support OR psychosocial OR psycholog* OR disclos* OR social rejection OR human rights OR patient rights OR negative belief OR negative opinion OR hate OR dignity OR disclosure OR blame

10679 HITS -too many to retrieve.

Applied 3 limits, and this reduced to 818:

limit 3 to (human and exclude medline journals and (Dutch Or English Or French Or German Or Indonesian Or Italian Or Japanese Or Latvian Or Portuguese Or Spanish Or Turkish Or Vietnamese))

Only language inclusions: (Dutch Or English Or French Or German Or Indonesian Or Italian Or Japanese Or Latvian Or Portuguese Or Spanish Or Turkish Or Vietnamese))

SOCIOFILE

Only language inclusions: (Dutch Or English Or French Or German Or Indonesian Or Italian Or Japanese Or Latvian Or Portuguese Or Spanish Or Turkish Or Vietnamese))

**Mental Health (Depression and Schizophrenia)**

PubMed

((((depression[Title/Abstract]) OR schizophrenia[Title/Abstract] AND

(Afghanistan[Title/Abstract] OR Albania[Title/Abstract] OR Algeria[Title/Abstract] OR Samoa[Title/Abstract] OR Angola[Title/Abstract] OR Antigua[Title/Abstract] OR Barbuda[Title/Abstract] OR Argentina[Title/Abstract] OR Armenia[Title/Abstract] OR Azerbaijan[Title/Abstract] OR Bangladesh[Title/Abstract] OR Belarus[Title/Abstract] OR Belize[Title/Abstract] OR Benin[Title/Abstract] OR Bhutan[Title/Abstract] OR Bolivia[Title/Abstract] OR Bosnia[Title/Abstract] OR Herzegovina[Title/Abstract] OR Botswana[Title/Abstract] OR Brazil[Title/Abstract] OR Bulgaria[Title/Abstract] OR Burkina Faso[Title/Abstract] OR Burundi[Title/Abstract] OR Cambodia[Title/Abstract] OR Cameroon[Title/Abstract] OR Cabo Verde[Title/Abstract] OR Central African Republic[Title/Abstract] OR Chad[Title/Abstract] OR Chile[Title/Abstract] OR China[Title/Abstract] OR Colombia[Title/Abstract] OR Comoros[Title/Abstract] OR Congo[Title/Abstract] OR Costa Rica[Title/Abstract] OR Côte d'Ivoire[Title/Abstract] OR Cote d’Ivoire[Title/Abstract] OR Ivory[Title/Abstract] OR Cuba[Title/Abstract] OR Djibouti[Title/Abstract] OR Dominica[Title/Abstract] OR Dominican[Title/Abstract] OR Ecuador[Title/Abstract] OR Egypt[Title/Abstract] OR Salvador[Title/Abstract] OR Eritrea[Title/Abstract] OR Ethiopia[Title/Abstract] OR Fiji[Title/Abstract] OR Gabon[Title/Abstract] OR Gambia[Title/Abstract] OR Georgia[Title/Abstract] OR Ghana[Title/Abstract] OR Grenada[Title/Abstract] OR Guatemala[Title/Abstract] OR Guinea[Title/Abstract] OR Guinea-Bissau[Title/Abstract] OR Guyana[Title/Abstract] OR Haiti[Title/Abstract] OR Honduras[Title/Abstract] OR India[Title/Abstract] OR Indonesia[Title/Abstract] OR Iran[Title/Abstract] OR Iraq[Title/Abstract] OR Jamaica[Title/Abstract] OR Jordan[Title/Abstract] OR Kazakhstan[Title/Abstract] OR Kenya[Title/Abstract] OR Kiribati[Title/Abstract] OR Korea [Title/Abstract] OR Kosovo[Title/Abstract] OR Kyrgyz [Title/Abstract] OR Lao[Title/Abstract] OR Laos[Title/Abstract] OR Latvia[Title/Abstract] OR Lebanon[Title/Abstract] OR Lesotho[Title/Abstract] OR Liberia[Title/Abstract] OR Libya[Title/Abstract] OR Lithuania[Title/Abstract] OR Macedonia[Title/Abstract] OR Madagascar[Title/Abstract] OR Malawi[Title/Abstract] OR Malaysia[Title/Abstract] OR Maldives[Title/Abstract] OR Mali[Title/Abstract] OR Marshall[Title/Abstract] OR Mauritania[Title/Abstract] OR Mauritius[Title/Abstract] OR Mexico[Title/Abstract] OR Micronesia[Title/Abstract] OR Moldova[Title/Abstract] OR Mongolia[Title/Abstract] OR Montenegro[Title/Abstract] OR Morocco[Title/Abstract] OR Mozambique[Title/Abstract] OR Myanmar[Title/Abstract] OR Namibia[Title/Abstract] OR Nepal[Title/Abstract] OR Nicaragua[Title/Abstract] OR Niger[Title/Abstract] OR Nigeria[Title/Abstract] OR Pakistan[Title/Abstract] OR Palau[Title/Abstract] OR Panama[Title/Abstract] OR Papua New Guinea[Title/Abstract] OR Paraguay[Title/Abstract] OR Peru[Title/Abstract] OR Philippines[Title/Abstract] OR Romania[Title/Abstract] OR Russia[Title/Abstract] OR Russian[Title/Abstract] OR Rwanda[Title/Abstract] OR Samoa[Title/Abstract] OR Sao Tome[Title/Abstract] OR Senegal[Title/Abstract] OR Serbia[Title/Abstract] OR Seychelles[Title/Abstract] OR Sierra Leone[Title/Abstract] OR Solomon Islands[Title/Abstract] OR Somalia[Title/Abstract] OR South Africa[Title/Abstract] OR Sri Lanka[Title/Abstract] OR St. Lucia[Title/Abstract] OR St. Vincent[Title/Abstract] OR Grenadines[Title/Abstract] OR Sudan[Title/Abstract] OR Suriname[Title/Abstract] OR Swaziland[Title/Abstract] OR Syrian[Title/Abstract] OR Syria[Title/Abstract] OR Tajikistan[Title/Abstract] OR Tanzania[Title/Abstract] OR Thailand[Title/Abstract] OR Timor-Leste[Title/Abstract] OR Togo[Title/Abstract] OR Tonga[Title/Abstract] OR Tunisia[Title/Abstract] OR Turkey[Title/Abstract] OR Turkmenistan[Title/Abstract] OR Tuvalu[Title/Abstract] OR Uganda[Title/Abstract] OR Ukraine[Title/Abstract] OR Uruguay[Title/Abstract] OR Uzbekistan[Title/Abstract] OR Vanuatu[Title/Abstract] OR Venezuela[Title/Abstract] OR Vietnam[Title/Abstract] OR “West Bank”[Title/Abstract] OR Gaza[Title/Abstract] OR Yemen[Title/Abstract] OR Zambia[Title/Abstract] OR Zimbabwe [Title/Abstract] OR “developing countries”[Title/Abstract] OR “resource-limited”[Title/Abstract] OR “resource-constrained”[Title/Abstract] OR “low- and middle-income”[Title/Abstract] OR LMIC[Title/Abstract] OR “third world”[Title/Abstract] OR “low income countries”[Title/Abstract]))) AND

("2008"[Date - Publication] : "3000"[Date - Publication])) AND

((stigma[Title/Abstract] OR stigmatize[Title/Abstract] OR stigmatise[Title/Abstract] OR stigmatizing[Title/Abstract] OR stigmatising[Title/Abstract] OR stereotype[Title/Abstract] OR stereotyping[Title/Abstract] OR shame[Title/Abstract] OR shaming[Title/Abstract] OR discrimination[Title/Abstract] OR discriminating[Title/Abstract] OR “social distance”[Title/Abstract] OR prejudice[Title/Abstract] OR blame[Title/Abstract]))

PsychInfo

(TX(depression) OR TX(schizophrenia)) AND

(TX(Afghanistan) OR TX(Albania) OR TX(Algeria) OR TX(Samoa) OR TX(Angola) OR TX(Antigua) OR TX(Barbuda) OR TX(Argentina) OR TX(Armenia) OR TX(Azerbaijan) OR TX(Bangladesh) OR TX(Belarus) OR TX(Belize) OR TX(Benin) OR TX(Bhutan) OR TX(Bolivia) OR TX(Bosnia) OR TX(Herzegovina) OR TX(Botswana) OR TX(Brazil) OR TX(Bulgaria) OR TX(Burkina Faso) OR TX(Burundi) OR TX(Cambodia) OR TX(Cameroon) OR TX(Cabo Verde) OR TX(Central African Republic) OR TX(Chad) OR TX(Chile) OR TX(China) OR TX(Colombia) OR TX(Comoros) OR TX(Congo) OR TX(Costa Rica) OR TX(Côte d'Ivoire) OR TX(Cote d’Ivoire) OR TX(Ivory) OR TX(Cuba) OR TX(Djibouti) OR TX(Dominica) OR TX(Dominican) OR TX(Ecuador) OR OR TX(Egypt) OR TX(Salvador) OR TX(Eritrea) OR TX(Ethiopia) OR TX(Fiji) OR TX(Gabon) OR TX(Gambia) OR TX(Georgia) OR TX(Ghana) OR TX(Grenada) OR TX(Guatemala) OR TX(Guinea) OR TX(Guinea-Bissau) OR TX(Guyana) OR TX(Haiti) OR TX(Honduras) OR TX(India) OR TX(Indonesia) OR TX(Iran) OR TX(Iraq) OR TX(Jamaica) OR TX(Jordan) OR TX(Kazakhstan) OR TX(Kenya) OR TX(Kiribati) OR TX(Korea) OR TX(Kosovo) OR TX(Kyrgyz) OR TX(Lao) OR TX(Laos) OR TX(Latvia) OR TX(Lebanon) OR TX(Lesotho) OR TX(Liberia) OR TX(Libya) OR TX(Lithuania) OR TX(Macedonia) OR TX(Madagascar) OR TX(Malawi) OR TX(Malaysia) OR TX(Maldives) OR TX(Mali) OR TX(Marshall) OR TX(Mauritania) OR TX(Mauritius) OR TX(Mexico) OR TX(Micronesia) OR TX(Moldova) OR TX(Mongolia) OR TX(Montenegro) OR TX(Morocco) OR TX(Mozambique) OR TX(Myanmar) OR TX(Namibia) OR TX(Nepal) OR TX(Nicaragua) OR TX(Niger) OR TX(Nigeria) OR TX(Pakistan) OR TX(Palau) OR TX(Panama) OR TX(Papua New Guinea) OR TX(Paraguay) OR TX(Peru) OR TX(Philippines) OR TX(Romania) OR TX(Russia) OR TX(Russian) OR TX(Rwanda) OR TX(Samoa) OR TX(Sao Tome) OR TX(Senegal) OR TX(Serbia) OR TX(Seychelles) OR TX(Sierra Leone) OR TX(Solomon Islands) OR TX(Somalia) OR TX(South Africa) OR TX(Sri Lanka) OR TX(St. Lucia) OR TX(St. Vincent) OR TX(Grenadines) OR TX(Sudan) OR TX(Suriname) OR TX(Swaziland) OR TX(Syrian) OR TX(Syria) OR TX(Tajikistan) OR TX(Tanzania) OR TX(Thailand) OR TX(Timor-Leste) OR TX(Togo) OR TX(Tonga) OR TX(Tunisia) OR TX(Turkey) OR TX(Turkmenistan) OR TX(Tuvalu) OR TX(Uganda) OR TX(Ukraine) OR TX(Uruguay) OR TX(Uzbekistan) OR TX(Vanuatu) OR TX(Venezuela) OR TX(Vietnam) OR TX(West Bank) OR TX(Gaza) OR TX(Yemen) OR TX(Zambia) OR TX(Zimbabwe) OR TX(developing countries) OR TX(resource-limited) OR TX(resource-constrained) OR TX(low- and middle-income) OR TX(LMIC) OR TX(third world) OR TX(low income countries))

AND

(TX(stigma) OR TX(stigmatize) OR TX(stigmatise) OR (TX)stigmatizing OR TX(stigmatizing) OR TX(stereotype) OR TX(stereotyping) OR TX(shame) OR TX(shaming) OR TX(discrimination) OR TX(discriminating) OR TX(social distance) OR TX(prejudice) OR TX(blame))

EMBASE

(depression:ti,ab OR ‘schizophrenia:ti,ab) AND

('developing country':ti,ab OR 'developing countries':ti,ab OR 'developing nation':ti,ab OR 'developing nations':ti,ab OR 'developing population':ti,ab OR 'developing populations':ti,ab OR 'developing world':ti,ab OR 'less developed country':ti,ab OR 'less developed countries':ti,ab OR 'less developed nation':ti,ab OR 'less developed nations':ti,ab OR 'less developed population':ti,ab OR 'less developed populations':ti,ab OR 'less developed world':ti,ab OR 'lesser developed country':ti,ab OR 'lesser developed countries':ti,ab OR 'lesser developed nation':ti,ab OR 'lesser developed nations':ti,ab OR 'lesser developed population':ti,ab OR 'lesser developed populations':ti,ab OR 'lesser developed world':ti,ab OR 'under developed country':ti,ab OR 'under developed countries':ti,ab OR 'under developed nation':ti,ab OR 'under developed nations':ti,ab OR 'under developed population':ti,ab OR 'under developed populations':ti,ab OR 'under developed world':ti,ab OR 'underdeveloped country':ti,ab OR 'underdeveloped countries':ti,ab OR 'underdeveloped nation':ti,ab OR 'underdeveloped nations':ti,ab OR 'underdeveloped population':ti,ab OR 'underdeveloped populations':ti,ab OR 'underdeveloped world':ti,ab OR 'middle income country':ti,ab OR 'middle income countries':ti,ab OR 'middle income nation':ti,ab OR 'middle income nations':ti,ab OR 'middle income population':ti,ab OR 'middle income populations':ti,ab OR 'low income country':ti,ab OR 'low income countries':ti,ab OR 'low income nation':ti,ab OR 'low income nations':ti,ab OR 'low income population':ti,ab OR 'low income populations':ti,ab OR 'lower income country':ti,ab OR 'lower income countries':ti,ab OR 'lower income nation':ti,ab OR 'lower income nations':ti,ab OR 'lower income population':ti,ab OR 'lower income populations':ti,ab OR 'underserved country':ti,ab OR 'underserved countries':ti,ab OR 'underserved nation':ti,ab OR 'underserved nations':ti,ab OR 'underserved population':ti,ab OR 'underserved populations':ti,ab OR 'underserved world':ti,ab OR 'under served country':ti,ab OR 'under served countries':ti,ab OR 'under served nation':ti,ab OR 'under served nations':ti,ab OR 'under served population':ti,ab OR 'under served populations':ti,ab OR 'under served world':ti,ab OR 'deprived country':ti,ab OR 'deprived countries':ti,ab OR 'deprived nation':ti,ab OR 'deprived nations':ti,ab OR 'deprived population':ti,ab OR 'deprived populations':ti,ab OR 'deprived world':ti,ab OR 'poor country':ti,ab OR 'poor countries':ti,ab OR 'poor nation':ti,ab OR 'poor nations':ti,ab OR 'poor population':ti,ab OR 'poor populations':ti,ab OR 'poor world':ti,ab OR 'poorer country':ti,ab OR 'poorer countries':ti,ab OR 'poorer nation':ti,ab OR 'poorer nations':ti,ab OR 'poorer population':ti,ab OR 'poorer populations':ti,ab OR 'poorer world':ti,ab OR 'developing economy':ti,ab OR 'developing economies':ti,ab OR 'less developed economy':ti,ab OR 'less developed economies':ti,ab OR 'lesser developed economy':ti,ab OR 'lesser developed economies':ti,ab OR 'under developed economy':ti,ab OR 'under developed economies':ti,ab OR 'underdeveloped economy':ti,ab OR 'underdeveloped economies':ti,ab OR 'middle income economy':ti,ab OR 'middle income economies':ti,ab OR 'low income economy':ti,ab OR 'low income economies':ti,ab OR 'lower income economy':ti,ab OR 'lower income economies':ti,ab OR 'low gdp':ti,ab OR 'low gnp':ti,ab OR 'low gross domestic':ti,ab OR 'low gross national':ti,ab OR 'lower gdp':ti,ab OR 'lower gnp':ti,ab OR 'lower gross domestic':ti,ab OR 'lower gross national':ti,ab OR lmic:ti,ab OR lmics:ti,ab OR 'third world':ti,ab OR 'lami country':ti,ab OR 'lami countries':ti,ab OR 'transitional country':ti,ab OR 'transitional countries':ti,ab OR africa:ti,ab OR asia:ti,ab OR caribbean:ti,ab OR 'west indies':ti,ab OR 'south america':ti,ab OR 'latin america':ti,ab OR 'central america':ti,ab OR 'atlantic islands':ti,ab OR 'commonwealth of independent states':ti,ab OR 'pacific islands':ti,ab OR 'indian ocean islands':ti,ab OR 'eastern europe':ti,ab OR afghanistan:ti,ab OR albania:ti,ab OR algeria:ti,ab OR angola:ti,ab OR antigua:ti,ab OR barbuda:ti,ab OR argentina:ti,ab OR armenia:ti,ab OR armenian:ti,ab OR aruba:ti,ab OR azerbaijan:ti,ab OR bahrain:ti,ab OR bangladesh:ti,ab OR barbados:ti,ab OR benin:ti,ab OR byelarus:ti,ab OR byelorussian:ti,ab OR belarus:ti,ab OR belorussian:ti,ab OR belorussia:ti,ab OR belize:ti,ab OR bhutan:ti,ab OR bolivia:ti,ab OR bosnia:ti,ab OR herzegovina:ti,ab OR hercegovina:ti,ab OR botswana:ti,ab OR brasil:ti,ab OR brazil:ti,ab OR bulgaria:ti,ab OR 'burkina faso':ti,ab OR 'burkina fasso':ti,ab OR 'upper volta':ti,ab OR burundi:ti,ab OR urundi:ti,ab OR cambodia:ti,ab OR 'khmer republic':ti,ab OR kampuchea:ti,ab OR cameroon:ti,ab OR cameroons:ti,ab OR cameron:ti,ab OR camerons:ti,ab OR cameroun:ti,ab OR 'cape verde':ti,ab OR 'central african republic':ti,ab OR chad:ti,ab OR chile:ti,ab OR china:ti,ab OR colombia:ti,ab OR comoros:ti,ab OR 'comoro islands':ti,ab OR comores:ti,ab OR mayotte:ti,ab OR congo:ti,ab OR zaire:ti,ab OR 'costa rica':ti,ab OR 'cote divoire':ti,ab OR 'ivory coast':ti,ab OR croatia:ti,ab OR cuba:ti,ab OR cyprus:ti,ab OR czechoslovakia:ti,ab OR 'czech republic':ti,ab OR slovakia:ti,ab OR 'slovak republic':ti,ab OR djibouti:ti,ab OR 'french somaliland':ti,ab OR dominica:ti,ab OR 'dominican republic':ti,ab OR 'east timor':ti,ab OR 'east timur':ti,ab OR 'timor leste':ti,ab OR ecuador:ti,ab OR egypt:ti,ab OR 'united arab republic':ti,ab OR 'el salvador':ti,ab OR eritrea:ti,ab OR estonia:ti,ab OR ethiopia:ti,ab OR fiji:ti,ab OR gabon:ti,ab OR 'gabonese republic':ti,ab OR gambia:ti,ab OR gaza:ti,ab OR 'georgia republic':ti,ab OR 'georgian republic':ti,ab OR ghana:ti,ab OR 'gold coast':ti,ab OR greece:ti,ab OR grenada:ti,ab OR guatemala:ti,ab OR guinea:ti,ab OR guam:ti,ab OR guiana:ti,ab OR guyana:ti,ab OR haiti:ti,ab OR honduras:ti,ab OR hungary:ti,ab OR india:ti,ab OR maldives:ti,ab OR indonesia:ti,ab OR iran:ti,ab OR iraq:ti,ab OR 'isle of man':ti,ab OR jamaica:ti,ab OR jordan:ti,ab OR kazakhstan:ti,ab OR kazakh:ti,ab OR kenya:ti,ab OR kiribati:ti,ab OR korea:ti,ab OR kosovo:ti,ab OR kyrgyzstan:ti,ab OR kirghizia:ti,ab OR 'kyrgyz republic':ti,ab OR kirghiz:ti,ab OR kirgizstan:ti,ab OR 'lao pdr':ti,ab OR laos:ti,ab OR latvia:ti,ab OR lebanon:ti,ab OR lesotho:ti,ab OR basutoland:ti,ab OR liberia:ti,ab OR libya:ti,ab OR lithuania:ti,ab OR macedonia:ti,ab OR madagascar:ti,ab OR 'malagasy republic':ti,ab OR malaysia:ti,ab OR malaya:ti,ab OR malay:ti,ab OR sabah:ti,ab OR sarawak:ti,ab OR malawi:ti,ab OR nyasaland:ti,ab OR mali:ti,ab OR malta:ti,ab OR 'marshall islands':ti,ab OR mauritania:ti,ab OR mauritius:ti,ab OR 'agalega islands':ti,ab OR 'melanesia':ti,ab OR mexico:ti,ab OR micronesia:ti,ab OR 'middle east':ti,ab OR moldova:ti,ab OR moldovia:ti,ab OR moldovian:ti,ab OR mongolia:ti,ab OR morocco:ti,ab OR mozambique:ti,ab OR myanmar:ti,ab OR myanma:ti,ab OR burma:ti,ab OR namibia:ti,ab OR nepal:ti,ab OR 'netherlands antilles':ti,ab OR 'new caledonia':ti,ab OR nicaragua:ti,ab OR niger:ti,ab OR nigeria:ti,ab OR 'northern mariana islands':ti,ab OR oman:ti,ab OR muscat:ti,ab OR pakistan:ti,ab OR palau:ti,ab OR palestine:ti,ab OR panama:ti,ab OR paraguay:ti,ab OR peru:ti,ab OR philippines:ti,ab OR philipines:ti,ab OR phillipines:ti,ab OR phillippines:ti,ab OR poland:ti,ab OR portugal:ti,ab OR 'puerto rico':ti,ab OR romania:ti,ab OR rumania:ti,ab OR roumania:ti,ab OR russia:ti,ab OR russian:ti,ab OR rwanda:ti,ab OR ruanda:ti,ab OR 'saint kitts':ti,ab OR 'st kitts':ti,ab OR nevis:ti,ab OR 'saint lucia':ti,ab OR 'st lucia':ti,ab OR 'saint vincent':ti,ab OR 'st vincent':ti,ab OR grenadines:ti,ab OR samoa:ti,ab OR 'samoan islands':ti,ab OR 'navigator island':ti,ab OR 'navigator islands':ti,ab OR 'sao tome':ti,ab OR 'saudi arabia':ti,ab OR senegal:ti,ab OR serbia:ti,ab OR montenegro:ti,ab OR seychelles:ti,ab OR 'sierra leone':ti,ab OR slovenia:ti,ab OR 'sri lanka':ti,ab OR ceylon:ti,ab OR 'solomon islands':ti,ab OR somalia:ti,ab OR sudan:ti,ab OR suriname:ti,ab OR surinam:ti,ab OR swaziland:ti,ab OR syria:ti,ab OR syrian:ti,ab OR tajikistan:ti,ab OR tadzhikistan:ti,ab OR tadjikistan:ti,ab OR tadzhik:ti,ab OR tanzania:ti,ab OR thailand:ti,ab OR togo:ti,ab OR 'togolese republic':ti,ab OR tonga:ti,ab OR trinidad:ti,ab OR tobago:ti,ab OR tunisia:ti,ab OR turkey:ti,ab OR turkmenistan:ti,ab OR turkmen:ti,ab OR tuvalu:ti,ab OR uganda:ti,ab OR ukraine:ti,ab OR uruguay:ti,ab OR ussr:ti,ab OR 'soviet union':ti,ab OR 'union of soviet socialist republics':ti,ab OR uzbekistan:ti,ab OR uzbek OR vanuatu:ti,ab OR 'new hebrides':ti,ab OR venezuela:ti,ab OR vietnam:ti,ab OR 'viet nam':ti,ab OR 'west bank':ti,ab OR yemen:ti,ab OR yugoslavia:ti,ab OR zambia:ti,ab OR zimbabwe:ti,ab OR rhodesia:ti,ab OR 'developing country'/exp OR 'africa'/de OR 'africa south of the sahara'/de OR 'central africa'/de OR 'asia'/de OR 'southeast asia'/de OR 'caribbean'/de OR 'caribbean islands'/de OR 'south america'/de OR 'south and central america'/de OR 'atlantic islands'/de OR 'ussr'/de OR 'pacific islands'/de OR 'indian ocean'/de OR 'eastern europe'/de OR 'afghanistan'/exp OR 'albania'/exp OR 'algeria'/exp OR 'american samoa'/exp OR 'angola'/exp OR 'antigua and barbuda'/exp OR 'argentina'/exp OR 'armenia'/exp OR 'azerbaijan'/exp OR 'bahrain'/exp OR 'baltic states'/exp OR 'bangladesh'/exp OR 'barbados'/exp OR 'benin'/exp OR 'belarus'/exp OR 'belize'/exp OR 'bhutan'/exp OR 'bolivia'/exp OR 'bosnia and herzegovina'/exp OR 'botswana'/exp OR 'brazil'/exp OR 'bulgaria'/exp OR 'burkina faso'/exp OR 'burundi'/exp OR 'cambodia'/exp OR 'cameroon'/exp OR 'cape verde' OR 'central african republic'/exp OR 'chad'/exp OR 'chile'/exp OR 'china'/exp OR 'colombia'/exp OR 'comoros'/exp OR 'congo'/exp OR 'costa rica'/exp OR 'cote divoire' OR 'croatia'/exp OR 'cuba'/exp OR 'cyprus'/exp OR 'czechoslovakia'/exp OR 'czech republic'/exp OR 'slovakia'/exp OR 'djibouti'/exp OR 'democratic republic congo'/exp OR 'north korea'/exp OR 'dominica'/exp OR 'dominican republic'/exp OR 'dominican (dominican republic)'/exp OR 'timor leste'/exp OR 'ecuador'/exp OR 'egypt'/exp OR 'el salvador'/exp OR 'eritrea'/exp OR 'estonia'/exp OR 'ethiopia'/exp OR 'equatorial guinea'/exp OR 'fiji'/exp OR 'french guiana'/exp OR 'gabon'/exp OR 'gambia'/exp OR 'georgia (republic)'/exp OR 'ghana'/exp OR 'greece'/exp OR 'grenada'/exp OR 'guatemala'/exp OR 'guinea'/exp OR 'guinea bissau'/exp OR 'guam'/exp OR 'guyana'/exp OR 'haiti'/exp OR 'honduras'/exp OR 'hungary'/exp OR 'samoa'/exp OR 'india'/exp OR 'indonesia'/exp OR 'iran'/exp OR 'iraq'/exp OR 'jamaica'/exp OR 'jordan'/exp OR 'kazakhstan'/exp OR 'kenya'/exp OR 'south korea'/exp OR 'kyrgyzstan'/exp OR 'laos'/exp OR 'latvia'/exp OR 'lebanon'/exp OR 'lesotho'/exp OR 'liberia'/exp OR 'libyan arab jamahiriya'/exp OR 'lithuania'/exp OR 'macedonia (republic)'/exp OR 'madagascar'/exp OR 'malawi'/exp OR 'malaysia'/exp OR 'mali'/exp OR 'malta'/exp OR 'mauritania'/exp OR 'mauritius'/exp OR 'melanesia'/exp OR 'mexico'/exp OR 'federated states of micronesia'/exp OR 'middle east'/de OR 'moldova'/exp OR 'mongolia'/exp OR 'morocco'/exp OR 'mozambique'/exp OR 'myanmar'/exp OR 'namibia'/exp OR 'nepal'/exp OR 'netherlands antilles'/exp OR 'new caledonia'/exp OR 'nicaragua'/exp OR 'niger'/exp OR 'nigeria'/exp OR 'oman'/exp OR 'pakistan'/exp OR 'palau'/exp OR 'panama'/exp OR 'papua new guinea'/exp OR 'paraguay'/exp OR 'peru'/exp OR 'philippines'/exp OR 'poland'/exp OR 'portugal'/exp OR 'puerto rico'/exp OR 'romania'/exp OR 'russian federation'/exp OR 'rwanda'/exp OR 'saint kitts and nevis'/exp OR 'saint lucia'/exp OR 'saint vincent and the grenadines'/exp OR 'saudi arabia'/exp OR 'senegal'/exp OR 'serbia'/exp OR 'montenegro (republic)'/exp OR 'seychelles'/exp OR 'sierra leone'/exp OR 'slovenia'/exp OR 'sri lanka'/exp OR 'somalia'/exp OR 'south africa'/exp OR 'sudan'/exp OR 'suriname'/exp OR 'swaziland'/exp OR 'syrian arab republic'/exp OR 'tajikistan'/exp OR 'tanzania'/exp OR 'thailand'/exp OR 'togo'/exp OR 'tonga'/exp OR 'trinidad and tobago'/exp OR 'tunisia'/exp OR 'turkey (republic)'/exp OR 'turkmenistan'/exp OR 'uganda'/exp OR 'ukraine'/exp OR 'uruguay'/exp OR 'uzbekistan'/exp OR 'vanuatu'/exp OR 'venezuela'/exp OR 'viet nam'/exp OR 'yemen'/exp OR 'yugoslavia'/exp OR 'yugoslavia (pre-1992)' OR 'zambia'/exp OR 'zimbabwe'/exp) AND

(stigma:ti,ab OR stigmatize:ti,ab OR stigmatise:ti,ab OR stigmatizing:ti,ab OR stigmatizing:ti,ab OR stereotype:ti,ab OR stereotyping:ti,ab OR shame:ti,ab OR shaming:ti,ab OR discrimination:ti,ab OR discriminating:ti,ab OR ‘social distance’:ti,ab OR prejudice:ti,ab OR blame:ti,ab)

AND

[2008-2017]/py

**Epilepsy**

PubMed

((((epilepsy[Title/Abstract]) OR neurological disorder[Title/Abstract] AND

(Afghanistan[Title/Abstract] OR Albania[Title/Abstract] OR Algeria[Title/Abstract] OR Samoa[Title/Abstract] OR Angola[Title/Abstract] OR Antigua[Title/Abstract] OR Barbuda[Title/Abstract] OR Argentina[Title/Abstract] OR Armenia[Title/Abstract] OR Azerbaijan[Title/Abstract] OR Bangladesh[Title/Abstract] OR Belarus[Title/Abstract] OR Belize[Title/Abstract] OR Benin[Title/Abstract] OR Bhutan[Title/Abstract] OR Bolivia[Title/Abstract] OR Bosnia[Title/Abstract] OR Herzegovina[Title/Abstract] OR Botswana[Title/Abstract] OR Brazil[Title/Abstract] OR Bulgaria[Title/Abstract] OR Burkina Faso[Title/Abstract] OR Burundi[Title/Abstract] OR Cambodia[Title/Abstract] OR Cameroon[Title/Abstract] OR Cabo Verde[Title/Abstract] OR Central African Republic[Title/Abstract] OR Chad[Title/Abstract] OR Chile[Title/Abstract] OR China[Title/Abstract] OR Colombia[Title/Abstract] OR Comoros[Title/Abstract] OR Congo[Title/Abstract] OR Costa Rica[Title/Abstract] OR Côte d'Ivoire[Title/Abstract] OR Cote d’Ivoire[Title/Abstract] OR Ivory[Title/Abstract] OR Cuba[Title/Abstract] OR Djibouti[Title/Abstract] OR Dominica[Title/Abstract] OR Dominican[Title/Abstract] OR Ecuador[Title/Abstract] OR Egypt[Title/Abstract] OR Salvador[Title/Abstract] OR Eritrea[Title/Abstract] OR Ethiopia[Title/Abstract] OR Fiji[Title/Abstract] OR Gabon[Title/Abstract] OR Gambia[Title/Abstract] OR Georgia[Title/Abstract] OR Ghana[Title/Abstract] OR Grenada[Title/Abstract] OR Guatemala[Title/Abstract] OR Guinea[Title/Abstract] OR Guinea-Bissau[Title/Abstract] OR Guyana[Title/Abstract] OR Haiti[Title/Abstract] OR Honduras[Title/Abstract] OR India[Title/Abstract] OR Indonesia[Title/Abstract] OR Iran[Title/Abstract] OR Iraq[Title/Abstract] OR Jamaica[Title/Abstract] OR Jordan[Title/Abstract] OR Kazakhstan[Title/Abstract] OR Kenya[Title/Abstract] OR Kiribati[Title/Abstract] OR Korea [Title/Abstract] OR Kosovo[Title/Abstract] OR Kyrgyz [Title/Abstract] OR Lao[Title/Abstract] OR Laos[Title/Abstract] OR Latvia[Title/Abstract] OR Lebanon[Title/Abstract] OR Lesotho[Title/Abstract] OR Liberia[Title/Abstract] OR Libya[Title/Abstract] OR Lithuania[Title/Abstract] OR Macedonia[Title/Abstract] OR Madagascar[Title/Abstract] OR Malawi[Title/Abstract] OR Malaysia[Title/Abstract] OR Maldives[Title/Abstract] OR Mali[Title/Abstract] OR Marshall[Title/Abstract] OR Mauritania[Title/Abstract] OR Mauritius[Title/Abstract] OR Mexico[Title/Abstract] OR Micronesia[Title/Abstract] OR Moldova[Title/Abstract] OR Mongolia[Title/Abstract] OR Montenegro[Title/Abstract] OR Morocco[Title/Abstract] OR Mozambique[Title/Abstract] OR Myanmar[Title/Abstract] OR Namibia[Title/Abstract] OR Nepal[Title/Abstract] OR Nicaragua[Title/Abstract] OR Niger[Title/Abstract] OR Nigeria[Title/Abstract] OR Pakistan[Title/Abstract] OR Palau[Title/Abstract] OR Panama[Title/Abstract] OR Papua New Guinea[Title/Abstract] OR Paraguay[Title/Abstract] OR Peru[Title/Abstract] OR Philippines[Title/Abstract] OR Romania[Title/Abstract] OR Russia[Title/Abstract] OR Russian[Title/Abstract] OR Rwanda[Title/Abstract] OR Samoa[Title/Abstract] OR Sao Tome[Title/Abstract] OR Senegal[Title/Abstract] OR Serbia[Title/Abstract] OR Seychelles[Title/Abstract] OR Sierra Leone[Title/Abstract] OR Solomon Islands[Title/Abstract] OR Somalia[Title/Abstract] OR South Africa[Title/Abstract] OR Sri Lanka[Title/Abstract] OR St. Lucia[Title/Abstract] OR St. Vincent[Title/Abstract] OR Grenadines[Title/Abstract] OR Sudan[Title/Abstract] OR Suriname[Title/Abstract] OR Swaziland[Title/Abstract] OR Syrian[Title/Abstract] OR Syria[Title/Abstract] OR Tajikistan[Title/Abstract] OR Tanzania[Title/Abstract] OR Thailand[Title/Abstract] OR Timor-Leste[Title/Abstract] OR Togo[Title/Abstract] OR Tonga[Title/Abstract] OR Tunisia[Title/Abstract] OR Turkey[Title/Abstract] OR Turkmenistan[Title/Abstract] OR Tuvalu[Title/Abstract] OR Uganda[Title/Abstract] OR Ukraine[Title/Abstract] OR Uruguay[Title/Abstract] OR Uzbekistan[Title/Abstract] OR Vanuatu[Title/Abstract] OR Venezuela[Title/Abstract] OR Vietnam[Title/Abstract] OR “West Bank”[Title/Abstract] OR Gaza[Title/Abstract] OR Yemen[Title/Abstract] OR Zambia[Title/Abstract] OR Zimbabwe [Title/Abstract] OR “developing countries”[Title/Abstract] OR “resource-limited”[Title/Abstract] OR “resource-constrained”[Title/Abstract] OR “low- and middle-income”[Title/Abstract] OR LMIC[Title/Abstract] OR “third world”[Title/Abstract] OR “low income countries”[Title/Abstract]))) AND

("2008"[Date - Publication] : "3000"[Date - Publication])) AND

((stigma[Title/Abstract] OR stigmatize[Title/Abstract] OR stigmatise[Title/Abstract] OR stigmatizing[Title/Abstract] OR stigmatising[Title/Abstract] OR stereotype[Title/Abstract] OR stereotyping[Title/Abstract] OR shame[Title/Abstract] OR shaming[Title/Abstract] OR discrimination[Title/Abstract] OR discriminating[Title/Abstract] OR “social distance”[Title/Abstract] OR prejudice[Title/Abstract] OR blame[Title/Abstract]))

PsychInfo

(TX(epilepsy) OR TX(neurological disorder)) AND

(TX(Afghanistan) OR TX(Albania) OR TX(Algeria) OR TX(Samoa) OR TX(Angola) OR TX(Antigua) OR TX(Barbuda) OR TX(Argentina) OR TX(Armenia) OR TX(Azerbaijan) OR TX(Bangladesh) OR TX(Belarus) OR TX(Belize) OR TX(Benin) OR TX(Bhutan) OR TX(Bolivia) OR TX(Bosnia) OR TX(Herzegovina) OR TX(Botswana) OR TX(Brazil) OR TX(Bulgaria) OR TX(Burkina Faso) OR TX(Burundi) OR TX(Cambodia) OR TX(Cameroon) OR TX(Cabo Verde) OR TX(Central African Republic) OR TX(Chad) OR TX(Chile) OR TX(China) OR TX(Colombia) OR TX(Comoros) OR TX(Congo) OR TX(Costa Rica) OR TX(Côte d'Ivoire) OR TX(Cote d’Ivoire) OR TX(Ivory) OR TX(Cuba) OR TX(Djibouti) OR TX(Dominica) OR TX(Dominican) OR TX(Ecuador) OR OR TX(Egypt) OR TX(Salvador) OR TX(Eritrea) OR TX(Ethiopia) OR TX(Fiji) OR TX(Gabon) OR TX(Gambia) OR TX(Georgia) OR TX(Ghana) OR TX(Grenada) OR TX(Guatemala) OR TX(Guinea) OR TX(Guinea-Bissau) OR TX(Guyana) OR TX(Haiti) OR TX(Honduras) OR TX(India) OR TX(Indonesia) OR TX(Iran) OR TX(Iraq) OR TX(Jamaica) OR TX(Jordan) OR TX(Kazakhstan) OR TX(Kenya) OR TX(Kiribati) OR TX(Korea) OR TX(Kosovo) OR TX(Kyrgyz) OR TX(Lao) OR TX(Laos) OR TX(Latvia) OR TX(Lebanon) OR TX(Lesotho) OR TX(Liberia) OR TX(Libya) OR TX(Lithuania) OR TX(Macedonia) OR TX(Madagascar) OR TX(Malawi) OR TX(Malaysia) OR TX(Maldives) OR TX(Mali) OR TX(Marshall) OR TX(Mauritania) OR TX(Mauritius) OR TX(Mexico) OR TX(Micronesia) OR TX(Moldova) OR TX(Mongolia) OR TX(Montenegro) OR TX(Morocco) OR TX(Mozambique) OR TX(Myanmar) OR TX(Namibia) OR TX(Nepal) OR TX(Nicaragua) OR TX(Niger) OR TX(Nigeria) OR TX(Pakistan) OR TX(Palau) OR TX(Panama) OR TX(Papua New Guinea) OR TX(Paraguay) OR TX(Peru) OR TX(Philippines) OR TX(Romania) OR TX(Russia) OR TX(Russian) OR TX(Rwanda) OR TX(Samoa) OR TX(Sao Tome) OR TX(Senegal) OR TX(Serbia) OR TX(Seychelles) OR TX(Sierra Leone) OR TX(Solomon Islands) OR TX(Somalia) OR TX(South Africa) OR TX(Sri Lanka) OR TX(St. Lucia) OR TX(St. Vincent) OR TX(Grenadines) OR TX(Sudan) OR TX(Suriname) OR TX(Swaziland) OR TX(Syrian) OR TX(Syria) OR TX(Tajikistan) OR TX(Tanzania) OR TX(Thailand) OR TX(Timor-Leste) OR TX(Togo) OR TX(Tonga) OR TX(Tunisia) OR TX(Turkey) OR TX(Turkmenistan) OR TX(Tuvalu) OR TX(Uganda) OR TX(Ukraine) OR TX(Uruguay) OR TX(Uzbekistan) OR TX(Vanuatu) OR TX(Venezuela) OR TX(Vietnam) OR TX(West Bank) OR TX(Gaza) OR TX(Yemen) OR TX(Zambia) OR TX(Zimbabwe) OR TX(developing countries) OR TX(resource-limited) OR TX(resource-constrained) OR TX(low- and middle-income) OR TX(LMIC) OR TX(third world) OR TX(low income countries))

AND

(TX(stigma) OR TX(stigmatize) OR TX(stigmatise) OR (TX)stigmatizing OR TX(stigmatizing) OR TX(stereotype) OR TX(stereotyping) OR TX(shame) OR TX(shaming) OR TX(discrimination) OR TX(discriminating) OR TX(social distance) OR TX(prejudice) OR TX(blame))

EMBASE

(epilepsy:ti,ab OR ‘neurological disorder’:ti,ab) AND

('developing country':ti,ab OR 'developing countries':ti,ab OR 'developing nation':ti,ab OR 'developing nations':ti,ab OR 'developing population':ti,ab OR 'developing populations':ti,ab OR 'developing world':ti,ab OR 'less developed country':ti,ab OR 'less developed countries':ti,ab OR 'less developed nation':ti,ab OR 'less developed nations':ti,ab OR 'less developed population':ti,ab OR 'less developed populations':ti,ab OR 'less developed world':ti,ab OR 'lesser developed country':ti,ab OR 'lesser developed countries':ti,ab OR 'lesser developed nation':ti,ab OR 'lesser developed nations':ti,ab OR 'lesser developed population':ti,ab OR 'lesser developed populations':ti,ab OR 'lesser developed world':ti,ab OR 'under developed country':ti,ab OR 'under developed countries':ti,ab OR 'under developed nation':ti,ab OR 'under developed nations':ti,ab OR 'under developed population':ti,ab OR 'under developed populations':ti,ab OR 'under developed world':ti,ab OR 'underdeveloped country':ti,ab OR 'underdeveloped countries':ti,ab OR 'underdeveloped nation':ti,ab OR 'underdeveloped nations':ti,ab OR 'underdeveloped population':ti,ab OR 'underdeveloped populations':ti,ab OR 'underdeveloped world':ti,ab OR 'middle income country':ti,ab OR 'middle income countries':ti,ab OR 'middle income nation':ti,ab OR 'middle income nations':ti,ab OR 'middle income population':ti,ab OR 'middle income populations':ti,ab OR 'low income country':ti,ab OR 'low income countries':ti,ab OR 'low income nation':ti,ab OR 'low income nations':ti,ab OR 'low income population':ti,ab OR 'low income populations':ti,ab OR 'lower income country':ti,ab OR 'lower income countries':ti,ab OR 'lower income nation':ti,ab OR 'lower income nations':ti,ab OR 'lower income population':ti,ab OR 'lower income populations':ti,ab OR 'underserved country':ti,ab OR 'underserved countries':ti,ab OR 'underserved nation':ti,ab OR 'underserved nations':ti,ab OR 'underserved population':ti,ab OR 'underserved populations':ti,ab OR 'underserved world':ti,ab OR 'under served country':ti,ab OR 'under served countries':ti,ab OR 'under served nation':ti,ab OR 'under served nations':ti,ab OR 'under served population':ti,ab OR 'under served populations':ti,ab OR 'under served world':ti,ab OR 'deprived country':ti,ab OR 'deprived countries':ti,ab OR 'deprived nation':ti,ab OR 'deprived nations':ti,ab OR 'deprived population':ti,ab OR 'deprived populations':ti,ab OR 'deprived world':ti,ab OR 'poor country':ti,ab OR 'poor countries':ti,ab OR 'poor nation':ti,ab OR 'poor nations':ti,ab OR 'poor population':ti,ab OR 'poor populations':ti,ab OR 'poor world':ti,ab OR 'poorer country':ti,ab OR 'poorer countries':ti,ab OR 'poorer nation':ti,ab OR 'poorer nations':ti,ab OR 'poorer population':ti,ab OR 'poorer populations':ti,ab OR 'poorer world':ti,ab OR 'developing economy':ti,ab OR 'developing economies':ti,ab OR 'less developed economy':ti,ab OR 'less developed economies':ti,ab OR 'lesser developed economy':ti,ab OR 'lesser developed economies':ti,ab OR 'under developed economy':ti,ab OR 'under developed economies':ti,ab OR 'underdeveloped economy':ti,ab OR 'underdeveloped economies':ti,ab OR 'middle income economy':ti,ab OR 'middle income economies':ti,ab OR 'low income economy':ti,ab OR 'low income economies':ti,ab OR 'lower income economy':ti,ab OR 'lower income economies':ti,ab OR 'low gdp':ti,ab OR 'low gnp':ti,ab OR 'low gross domestic':ti,ab OR 'low gross national':ti,ab OR 'lower gdp':ti,ab OR 'lower gnp':ti,ab OR 'lower gross domestic':ti,ab OR 'lower gross national':ti,ab OR lmic:ti,ab OR lmics:ti,ab OR 'third world':ti,ab OR 'lami country':ti,ab OR 'lami countries':ti,ab OR 'transitional country':ti,ab OR 'transitional countries':ti,ab OR africa:ti,ab OR asia:ti,ab OR caribbean:ti,ab OR 'west indies':ti,ab OR 'south america':ti,ab OR 'latin america':ti,ab OR 'central america':ti,ab OR 'atlantic islands':ti,ab OR 'commonwealth of independent states':ti,ab OR 'pacific islands':ti,ab OR 'indian ocean islands':ti,ab OR 'eastern europe':ti,ab OR afghanistan:ti,ab OR albania:ti,ab OR algeria:ti,ab OR angola:ti,ab OR antigua:ti,ab OR barbuda:ti,ab OR argentina:ti,ab OR armenia:ti,ab OR armenian:ti,ab OR aruba:ti,ab OR azerbaijan:ti,ab OR bahrain:ti,ab OR bangladesh:ti,ab OR barbados:ti,ab OR benin:ti,ab OR byelarus:ti,ab OR byelorussian:ti,ab OR belarus:ti,ab OR belorussian:ti,ab OR belorussia:ti,ab OR belize:ti,ab OR bhutan:ti,ab OR bolivia:ti,ab OR bosnia:ti,ab OR herzegovina:ti,ab OR hercegovina:ti,ab OR botswana:ti,ab OR brasil:ti,ab OR brazil:ti,ab OR bulgaria:ti,ab OR 'burkina faso':ti,ab OR 'burkina fasso':ti,ab OR 'upper volta':ti,ab OR burundi:ti,ab OR urundi:ti,ab OR cambodia:ti,ab OR 'khmer republic':ti,ab OR kampuchea:ti,ab OR cameroon:ti,ab OR cameroons:ti,ab OR cameron:ti,ab OR camerons:ti,ab OR cameroun:ti,ab OR 'cape verde':ti,ab OR 'central african republic':ti,ab OR chad:ti,ab OR chile:ti,ab OR china:ti,ab OR colombia:ti,ab OR comoros:ti,ab OR 'comoro islands':ti,ab OR comores:ti,ab OR mayotte:ti,ab OR congo:ti,ab OR zaire:ti,ab OR 'costa rica':ti,ab OR 'cote divoire':ti,ab OR 'ivory coast':ti,ab OR croatia:ti,ab OR cuba:ti,ab OR cyprus:ti,ab OR czechoslovakia:ti,ab OR 'czech republic':ti,ab OR slovakia:ti,ab OR 'slovak republic':ti,ab OR djibouti:ti,ab OR 'french somaliland':ti,ab OR dominica:ti,ab OR 'dominican republic':ti,ab OR 'east timor':ti,ab OR 'east timur':ti,ab OR 'timor leste':ti,ab OR ecuador:ti,ab OR egypt:ti,ab OR 'united arab republic':ti,ab OR 'el salvador':ti,ab OR eritrea:ti,ab OR estonia:ti,ab OR ethiopia:ti,ab OR fiji:ti,ab OR gabon:ti,ab OR 'gabonese republic':ti,ab OR gambia:ti,ab OR gaza:ti,ab OR 'georgia republic':ti,ab OR 'georgian republic':ti,ab OR ghana:ti,ab OR 'gold coast':ti,ab OR greece:ti,ab OR grenada:ti,ab OR guatemala:ti,ab OR guinea:ti,ab OR guam:ti,ab OR guiana:ti,ab OR guyana:ti,ab OR haiti:ti,ab OR honduras:ti,ab OR hungary:ti,ab OR india:ti,ab OR maldives:ti,ab OR indonesia:ti,ab OR iran:ti,ab OR iraq:ti,ab OR 'isle of man':ti,ab OR jamaica:ti,ab OR jordan:ti,ab OR kazakhstan:ti,ab OR kazakh:ti,ab OR kenya:ti,ab OR kiribati:ti,ab OR korea:ti,ab OR kosovo:ti,ab OR kyrgyzstan:ti,ab OR kirghizia:ti,ab OR 'kyrgyz republic':ti,ab OR kirghiz:ti,ab OR kirgizstan:ti,ab OR 'lao pdr':ti,ab OR laos:ti,ab OR latvia:ti,ab OR lebanon:ti,ab OR lesotho:ti,ab OR basutoland:ti,ab OR liberia:ti,ab OR libya:ti,ab OR lithuania:ti,ab OR macedonia:ti,ab OR madagascar:ti,ab OR 'malagasy republic':ti,ab OR malaysia:ti,ab OR malaya:ti,ab OR malay:ti,ab OR sabah:ti,ab OR sarawak:ti,ab OR malawi:ti,ab OR nyasaland:ti,ab OR mali:ti,ab OR malta:ti,ab OR 'marshall islands':ti,ab OR mauritania:ti,ab OR mauritius:ti,ab OR 'agalega islands':ti,ab OR 'melanesia':ti,ab OR mexico:ti,ab OR micronesia:ti,ab OR 'middle east':ti,ab OR moldova:ti,ab OR moldovia:ti,ab OR moldovian:ti,ab OR mongolia:ti,ab OR morocco:ti,ab OR mozambique:ti,ab OR myanmar:ti,ab OR myanma:ti,ab OR burma:ti,ab OR namibia:ti,ab OR nepal:ti,ab OR 'netherlands antilles':ti,ab OR 'new caledonia':ti,ab OR nicaragua:ti,ab OR niger:ti,ab OR nigeria:ti,ab OR 'northern mariana islands':ti,ab OR oman:ti,ab OR muscat:ti,ab OR pakistan:ti,ab OR palau:ti,ab OR palestine:ti,ab OR panama:ti,ab OR paraguay:ti,ab OR peru:ti,ab OR philippines:ti,ab OR philipines:ti,ab OR phillipines:ti,ab OR phillippines:ti,ab OR poland:ti,ab OR portugal:ti,ab OR 'puerto rico':ti,ab OR romania:ti,ab OR rumania:ti,ab OR roumania:ti,ab OR russia:ti,ab OR russian:ti,ab OR rwanda:ti,ab OR ruanda:ti,ab OR 'saint kitts':ti,ab OR 'st kitts':ti,ab OR nevis:ti,ab OR 'saint lucia':ti,ab OR 'st lucia':ti,ab OR 'saint vincent':ti,ab OR 'st vincent':ti,ab OR grenadines:ti,ab OR samoa:ti,ab OR 'samoan islands':ti,ab OR 'navigator island':ti,ab OR 'navigator islands':ti,ab OR 'sao tome':ti,ab OR 'saudi arabia':ti,ab OR senegal:ti,ab OR serbia:ti,ab OR montenegro:ti,ab OR seychelles:ti,ab OR 'sierra leone':ti,ab OR slovenia:ti,ab OR 'sri lanka':ti,ab OR ceylon:ti,ab OR 'solomon islands':ti,ab OR somalia:ti,ab OR sudan:ti,ab OR suriname:ti,ab OR surinam:ti,ab OR swaziland:ti,ab OR syria:ti,ab OR syrian:ti,ab OR tajikistan:ti,ab OR tadzhikistan:ti,ab OR tadjikistan:ti,ab OR tadzhik:ti,ab OR tanzania:ti,ab OR thailand:ti,ab OR togo:ti,ab OR 'togolese republic':ti,ab OR tonga:ti,ab OR trinidad:ti,ab OR tobago:ti,ab OR tunisia:ti,ab OR turkey:ti,ab OR turkmenistan:ti,ab OR turkmen:ti,ab OR tuvalu:ti,ab OR uganda:ti,ab OR ukraine:ti,ab OR uruguay:ti,ab OR ussr:ti,ab OR 'soviet union':ti,ab OR 'union of soviet socialist republics':ti,ab OR uzbekistan:ti,ab OR uzbek OR vanuatu:ti,ab OR 'new hebrides':ti,ab OR venezuela:ti,ab OR vietnam:ti,ab OR 'viet nam':ti,ab OR 'west bank':ti,ab OR yemen:ti,ab OR yugoslavia:ti,ab OR zambia:ti,ab OR zimbabwe:ti,ab OR rhodesia:ti,ab OR 'developing country'/exp OR 'africa'/de OR 'africa south of the sahara'/de OR 'central africa'/de OR 'asia'/de OR 'southeast asia'/de OR 'caribbean'/de OR 'caribbean islands'/de OR 'south america'/de OR 'south and central america'/de OR 'atlantic islands'/de OR 'ussr'/de OR 'pacific islands'/de OR 'indian ocean'/de OR 'eastern europe'/de OR 'afghanistan'/exp OR 'albania'/exp OR 'algeria'/exp OR 'american samoa'/exp OR 'angola'/exp OR 'antigua and barbuda'/exp OR 'argentina'/exp OR 'armenia'/exp OR 'azerbaijan'/exp OR 'bahrain'/exp OR 'baltic states'/exp OR 'bangladesh'/exp OR 'barbados'/exp OR 'benin'/exp OR 'belarus'/exp OR 'belize'/exp OR 'bhutan'/exp OR 'bolivia'/exp OR 'bosnia and herzegovina'/exp OR 'botswana'/exp OR 'brazil'/exp OR 'bulgaria'/exp OR 'burkina faso'/exp OR 'burundi'/exp OR 'cambodia'/exp OR 'cameroon'/exp OR 'cape verde' OR 'central african republic'/exp OR 'chad'/exp OR 'chile'/exp OR 'china'/exp OR 'colombia'/exp OR 'comoros'/exp OR 'congo'/exp OR 'costa rica'/exp OR 'cote divoire' OR 'croatia'/exp OR 'cuba'/exp OR 'cyprus'/exp OR 'czechoslovakia'/exp OR 'czech republic'/exp OR 'slovakia'/exp OR 'djibouti'/exp OR 'democratic republic congo'/exp OR 'north korea'/exp OR 'dominica'/exp OR 'dominican republic'/exp OR 'dominican (dominican republic)'/exp OR 'timor leste'/exp OR 'ecuador'/exp OR 'egypt'/exp OR 'el salvador'/exp OR 'eritrea'/exp OR 'estonia'/exp OR 'ethiopia'/exp OR 'equatorial guinea'/exp OR 'fiji'/exp OR 'french guiana'/exp OR 'gabon'/exp OR 'gambia'/exp OR 'georgia (republic)'/exp OR 'ghana'/exp OR 'greece'/exp OR 'grenada'/exp OR 'guatemala'/exp OR 'guinea'/exp OR 'guinea bissau'/exp OR 'guam'/exp OR 'guyana'/exp OR 'haiti'/exp OR 'honduras'/exp OR 'hungary'/exp OR 'samoa'/exp OR 'india'/exp OR 'indonesia'/exp OR 'iran'/exp OR 'iraq'/exp OR 'jamaica'/exp OR 'jordan'/exp OR 'kazakhstan'/exp OR 'kenya'/exp OR 'south korea'/exp OR 'kyrgyzstan'/exp OR 'laos'/exp OR 'latvia'/exp OR 'lebanon'/exp OR 'lesotho'/exp OR 'liberia'/exp OR 'libyan arab jamahiriya'/exp OR 'lithuania'/exp OR 'macedonia (republic)'/exp OR 'madagascar'/exp OR 'malawi'/exp OR 'malaysia'/exp OR 'mali'/exp OR 'malta'/exp OR 'mauritania'/exp OR 'mauritius'/exp OR 'melanesia'/exp OR 'mexico'/exp OR 'federated states of micronesia'/exp OR 'middle east'/de OR 'moldova'/exp OR 'mongolia'/exp OR 'morocco'/exp OR 'mozambique'/exp OR 'myanmar'/exp OR 'namibia'/exp OR 'nepal'/exp OR 'netherlands antilles'/exp OR 'new caledonia'/exp OR 'nicaragua'/exp OR 'niger'/exp OR 'nigeria'/exp OR 'oman'/exp OR 'pakistan'/exp OR 'palau'/exp OR 'panama'/exp OR 'papua new guinea'/exp OR 'paraguay'/exp OR 'peru'/exp OR 'philippines'/exp OR 'poland'/exp OR 'portugal'/exp OR 'puerto rico'/exp OR 'romania'/exp OR 'russian federation'/exp OR 'rwanda'/exp OR 'saint kitts and nevis'/exp OR 'saint lucia'/exp OR 'saint vincent and the grenadines'/exp OR 'saudi arabia'/exp OR 'senegal'/exp OR 'serbia'/exp OR 'montenegro (republic)'/exp OR 'seychelles'/exp OR 'sierra leone'/exp OR 'slovenia'/exp OR 'sri lanka'/exp OR 'somalia'/exp OR 'south africa'/exp OR 'sudan'/exp OR 'suriname'/exp OR 'swaziland'/exp OR 'syrian arab republic'/exp OR 'tajikistan'/exp OR 'tanzania'/exp OR 'thailand'/exp OR 'togo'/exp OR 'tonga'/exp OR 'trinidad and tobago'/exp OR 'tunisia'/exp OR 'turkey (republic)'/exp OR 'turkmenistan'/exp OR 'uganda'/exp OR 'ukraine'/exp OR 'uruguay'/exp OR 'uzbekistan'/exp OR 'vanuatu'/exp OR 'venezuela'/exp OR 'viet nam'/exp OR 'yemen'/exp OR 'yugoslavia'/exp OR 'yugoslavia (pre-1992)' OR 'zambia'/exp OR 'zimbabwe'/exp) AND

(stigma:ti,ab OR stigmatize:ti,ab OR stigmatise:ti,ab OR stigmatizing:ti,ab OR stigmatizing:ti,ab OR stereotype:ti,ab OR stereotyping:ti,ab OR shame:ti,ab OR shaming:ti,ab OR discrimination:ti,ab OR discriminating:ti,ab OR ‘social distance’:ti,ab OR prejudice:ti,ab OR blame:ti,ab)

AND

[2008-2017]/py

**Substance use**

PubMed

(“hazardous alcohol use”[tiab] OR “alcohol misuse”[tiab] OR “alcohol use”[tiab] OR alcohol[tiab] OR “alcohol consumption”[tiab] OR “heavy drinking”[tiab] OR “binge drinking”[tiab] OR “alcohol use disorder”[tiab] OR “alcohol abuse”[tiab] OR “alcohol dependence”[tiab] OR alcoholism[tiab] OR alcoholic[tiab] OR “Drinking behavior”[MeSH] OR “Alcohol Abstinence”[MeSH] OR “Alcohol Drinking”[MeSH] OR “Binge Drinking”[MeSH] OR “Underage Drinking”[MeSH] OR “Alcohol-Related Disorders”[MeSH] OR “Alcohol Intoxication”[MeSH] OR Alcoholism[MeSH] OR “drug use” [tiab] OR “drug misuse” [tiab] OR “substance use” [tiab] OR “substance misuse” [tiab] OR “substance use disorder”[MeSH]) OR “substance abuse”[MeSH]) OR “substance abuse treatment centers”[MeSH] OR “drug use disorder”[MeSH] OR “drug abuse”[MeSH]) AND

(Afghanistan[Title/Abstract] OR Albania[Title/Abstract] OR Algeria[Title/Abstract] OR Samoa[Title/Abstract] OR Angola[Title/Abstract] OR Antigua[Title/Abstract] OR Barbuda[Title/Abstract] OR Argentina[Title/Abstract] OR Armenia[Title/Abstract] OR Azerbaijan[Title/Abstract] OR Bangladesh[Title/Abstract] OR Belarus[Title/Abstract] OR Belize[Title/Abstract] OR Benin[Title/Abstract] OR Bhutan[Title/Abstract] OR Bolivia[Title/Abstract] OR Bosnia[Title/Abstract] OR Herzegovina[Title/Abstract] OR Botswana[Title/Abstract] OR Brazil[Title/Abstract] OR Bulgaria[Title/Abstract] OR Burkina Faso[Title/Abstract] OR Burundi[Title/Abstract] OR Cambodia[Title/Abstract] OR Cameroon[Title/Abstract] OR Cabo Verde[Title/Abstract] OR Central African Republic[Title/Abstract] OR Chad[Title/Abstract] OR Chile[Title/Abstract] OR China[Title/Abstract] OR Colombia[Title/Abstract] OR Comoros[Title/Abstract] OR Congo[Title/Abstract] OR Costa Rica[Title/Abstract] OR Côte d'Ivoire[Title/Abstract] OR Cote d’Ivoire[Title/Abstract] OR Ivory[Title/Abstract] OR Cuba[Title/Abstract] OR Djibouti[Title/Abstract] OR Dominica[Title/Abstract] OR Dominican[Title/Abstract] OR Ecuador[Title/Abstract] OR Egypt[Title/Abstract] OR Salvador[Title/Abstract] OR Eritrea[Title/Abstract] OR Ethiopia[Title/Abstract] OR Fiji[Title/Abstract] OR Gabon[Title/Abstract] OR Gambia[Title/Abstract] OR Georgia[Title/Abstract] OR Ghana[Title/Abstract] OR Grenada[Title/Abstract] OR Guatemala[Title/Abstract] OR Guinea[Title/Abstract] OR Guinea-Bissau[Title/Abstract] OR Guyana[Title/Abstract] OR Haiti[Title/Abstract] OR Honduras[Title/Abstract] OR India[Title/Abstract] OR Indonesia[Title/Abstract] OR Iran[Title/Abstract] OR Iraq[Title/Abstract] OR Jamaica[Title/Abstract] OR Jordan[Title/Abstract] OR Kazakhstan[Title/Abstract] OR Kenya[Title/Abstract] OR Kiribati[Title/Abstract] OR Korea [Title/Abstract] OR Kosovo[Title/Abstract] OR Kyrgyz [Title/Abstract] OR Lao[Title/Abstract] OR Laos[Title/Abstract] OR Latvia[Title/Abstract] OR Lebanon[Title/Abstract] OR Lesotho[Title/Abstract] OR Liberia[Title/Abstract] OR Libya[Title/Abstract] OR Lithuania[Title/Abstract] OR Macedonia[Title/Abstract] OR Madagascar[Title/Abstract] OR Malawi[Title/Abstract] OR Malaysia[Title/Abstract] OR Maldives[Title/Abstract] OR Mali[Title/Abstract] OR Marshall[Title/Abstract] OR Mauritania[Title/Abstract] OR Mauritius[Title/Abstract] OR Mexico[Title/Abstract] OR Micronesia[Title/Abstract] OR Moldova[Title/Abstract] OR Mongolia[Title/Abstract] OR Montenegro[Title/Abstract] OR Morocco[Title/Abstract] OR Mozambique[Title/Abstract] OR Myanmar[Title/Abstract] OR Namibia[Title/Abstract] OR Nepal[Title/Abstract] OR Nicaragua[Title/Abstract] OR Niger[Title/Abstract] OR Nigeria[Title/Abstract] OR Pakistan[Title/Abstract] OR Palau[Title/Abstract] OR Panama[Title/Abstract] OR Papua New Guinea[Title/Abstract] OR Paraguay[Title/Abstract] OR Peru[Title/Abstract] OR Philippines[Title/Abstract] OR Romania[Title/Abstract] OR Russia[Title/Abstract] OR Russian[Title/Abstract] OR Rwanda[Title/Abstract] OR Samoa[Title/Abstract] OR Sao Tome[Title/Abstract] OR Senegal[Title/Abstract] OR Serbia[Title/Abstract] OR Seychelles[Title/Abstract] OR Sierra Leone[Title/Abstract] OR Solomon Islands[Title/Abstract] OR Somalia[Title/Abstract] OR South Africa[Title/Abstract] OR Sri Lanka[Title/Abstract] OR St. Lucia[Title/Abstract] OR St. Vincent[Title/Abstract] OR Grenadines[Title/Abstract] OR Sudan[Title/Abstract] OR Suriname[Title/Abstract] OR Swaziland[Title/Abstract] OR Syrian[Title/Abstract] OR Syria[Title/Abstract] OR Tajikistan[Title/Abstract] OR Tanzania[Title/Abstract] OR Thailand[Title/Abstract] OR Timor-Leste[Title/Abstract] OR Togo[Title/Abstract] OR Tonga[Title/Abstract] OR Tunisia[Title/Abstract] OR Turkey[Title/Abstract] OR Turkmenistan[Title/Abstract] OR Tuvalu[Title/Abstract] OR Uganda[Title/Abstract] OR Ukraine[Title/Abstract] OR Uruguay[Title/Abstract] OR Uzbekistan[Title/Abstract] OR Vanuatu[Title/Abstract] OR Venezuela[Title/Abstract] OR Vietnam[Title/Abstract] OR “West Bank”[Title/Abstract] OR Gaza[Title/Abstract] OR Yemen[Title/Abstract] OR Zambia[Title/Abstract] OR Zimbabwe [Title/Abstract] OR “developing countries”[Title/Abstract] OR “resource-limited”[Title/Abstract] OR “resource-constrained”[Title/Abstract] OR “low- and middle-income”[Title/Abstract] OR LMIC[Title/Abstract] OR “third world”[Title/Abstract] OR “low income countries”[Title/Abstract]))) AND

("2008"[Date - Publication] : "3000"[Date - Publication])) AND

((stigma[Title/Abstract] OR stigmatize[Title/Abstract] OR stigmatise[Title/Abstract] OR stigmatizing[Title/Abstract] OR stigmatising[Title/Abstract] OR stereotype[Title/Abstract] OR stereotyping[Title/Abstract] OR shame[Title/Abstract] OR shaming[Title/Abstract] OR discrimination[Title/Abstract] OR discriminating[Title/Abstract] OR “social distance”[Title/Abstract] OR prejudice[Title/Abstract] OR blame[Title/Abstract]))

PsychInfo

(TX(hazardous alcohol use) OR TX(alcohol misuse) OR TX(alcohol use) OR TX(alcohol) OR TX(alcohol consumption) OR TX(heavy drinking) OR TX(binge drinking) OR TX(alcohol use disorder) OR TX(alcohol abuse) OR TX(alcohol dependence) OR TX(alcoholism) OR TX(alcoholic) OR TX(Drinking behavior) OR TX(Alcohol Abstinence) OR TX(Alcohol Drinking) OR TX(Binge Drinking) OR TX(Underage Drinking) OR TX(Alcohol-Related Disorders) OR TX(Alcohol Intoxication) OR TX(Alcoholism) OR TX(drug use) OR TX(drug misuse) OR TX(substance use) OR TX(substance misuse) OR TX(substance use disorder) OR TX(substance abuse) OR TX(substance abuse treatment centers) OR TX(drug use disorder) OR TX(drug abuse)) AND

(TX(Afghanistan) OR TX(Albania) OR TX(Algeria) OR TX(Samoa) OR TX(Angola) OR TX(Antigua) OR TX(Barbuda) OR TX(Argentina) OR TX(Armenia) OR TX(Azerbaijan) OR TX(Bangladesh) OR TX(Belarus) OR TX(Belize) OR TX(Benin) OR TX(Bhutan) OR TX(Bolivia) OR TX(Bosnia) OR TX(Herzegovina) OR TX(Botswana) OR TX(Brazil) OR TX(Bulgaria) OR TX(Burkina Faso) OR TX(Burundi) OR TX(Cambodia) OR TX(Cameroon) OR TX(Cabo Verde) OR TX(Central African Republic) OR TX(Chad) OR TX(Chile) OR TX(China) OR TX(Colombia) OR TX(Comoros) OR TX(Congo) OR TX(Costa Rica) OR TX(Côte d'Ivoire) OR TX(Cote d’Ivoire) OR TX(Ivory) OR TX(Cuba) OR TX(Djibouti) OR TX(Dominica) OR TX(Dominican) OR TX(Ecuador) OR OR TX(Egypt) OR TX(Salvador) OR TX(Eritrea) OR TX(Ethiopia) OR TX(Fiji) OR TX(Gabon) OR TX(Gambia) OR TX(Georgia) OR TX(Ghana) OR TX(Grenada) OR TX(Guatemala) OR TX(Guinea) OR TX(Guinea-Bissau) OR TX(Guyana) OR TX(Haiti) OR TX(Honduras) OR TX(India) OR TX(Indonesia) OR TX(Iran) OR TX(Iraq) OR TX(Jamaica) OR TX(Jordan) OR TX(Kazakhstan) OR TX(Kenya) OR TX(Kiribati) OR TX(Korea) OR TX(Kosovo) OR TX(Kyrgyz) OR TX(Lao) OR TX(Laos) OR TX(Latvia) OR TX(Lebanon) OR TX(Lesotho) OR TX(Liberia) OR TX(Libya) OR TX(Lithuania) OR TX(Macedonia) OR TX(Madagascar) OR TX(Malawi) OR TX(Malaysia) OR TX(Maldives) OR TX(Mali) OR TX(Marshall) OR TX(Mauritania) OR TX(Mauritius) OR TX(Mexico) OR TX(Micronesia) OR TX(Moldova) OR TX(Mongolia) OR TX(Montenegro) OR TX(Morocco) OR TX(Mozambique) OR TX(Myanmar) OR TX(Namibia) OR TX(Nepal) OR TX(Nicaragua) OR TX(Niger) OR TX(Nigeria) OR TX(Pakistan) OR TX(Palau) OR TX(Panama) OR TX(Papua New Guinea) OR TX(Paraguay) OR TX(Peru) OR TX(Philippines) OR TX(Romania) OR TX(Russia) OR TX(Russian) OR TX(Rwanda) OR TX(Samoa) OR TX(Sao Tome) OR TX(Senegal) OR TX(Serbia) OR TX(Seychelles) OR TX(Sierra Leone) OR TX(Solomon Islands) OR TX(Somalia) OR TX(South Africa) OR TX(Sri Lanka) OR TX(St. Lucia) OR TX(St. Vincent) OR TX(Grenadines) OR TX(Sudan) OR TX(Suriname) OR TX(Swaziland) OR TX(Syrian) OR TX(Syria) OR TX(Tajikistan) OR TX(Tanzania) OR TX(Thailand) OR TX(Timor-Leste) OR TX(Togo) OR TX(Tonga) OR TX(Tunisia) OR TX(Turkey) OR TX(Turkmenistan) OR TX(Tuvalu) OR TX(Uganda) OR TX(Ukraine) OR TX(Uruguay) OR TX(Uzbekistan) OR TX(Vanuatu) OR TX(Venezuela) OR TX(Vietnam) OR TX(West Bank) OR TX(Gaza) OR TX(Yemen) OR TX(Zambia) OR TX(Zimbabwe) OR TX(developing countries) OR TX(resource-limited) OR TX(resource-constrained) OR TX(low- and middle-income) OR TX(LMIC) OR TX(third world) OR TX(low income countries))

AND

(TX(stigma) OR TX(stigmatize) OR TX(stigmatise) OR (TX)stigmatizing OR TX(stigmatizing) OR TX(stereotype) OR TX(stereotyping) OR TX(shame) OR TX(shaming) OR TX(discrimination) OR TX(discriminating) OR TX(social distance) OR TX(prejudice) OR TX(blame))

Embase

(‘hazardous alcohol use’:ti,ab OR ‘alcohol misuse’:ti,ab OR ‘alcohol use’:ti,ab OR alcohol:ti,ab OR ‘alcohol consumption’:ti,ab OR ‘heavy drinking’:ti,ab OR ‘binge drinking’:ti,ab OR ‘alcohol use disorder’:ti,ab OR ‘alcohol abuse’:ti,ab OR ‘alcohol dependence’:ti,ab OR alcoholism:ti,ab OR alcoholic:ti,ab OR ‘Drinking behavior’:ti,ab OR ‘Alcohol Abstinence’:ti,ab OR ‘Alcohol Drinking’:ti,ab OR ‘Binge Drinking’:ti,ab OR ‘Underage Drinking’:ti,ab OR ‘Alcohol-Related Disorders’:ti,ab OR ‘Alcohol Intoxication’:ti,ab OR Alcoholism:ti,ab OR ‘drug use’:ti,ab OR ‘drug misuse’:ti,ab OR ‘substance use’:ti,ab OR ‘substance misuse’:ti,ab OR ‘substance use disorder’:ti,ab OR ‘substance abuse’:ti,ab OR ‘substance abuse treatment centers’:ti,ab OR ‘drug use disorder’:ti,ab OR ‘drug abuse’:ti,ab) AND

('developing country':ti,ab OR 'developing countries':ti,ab OR 'developing nation':ti,ab OR 'developing nations':ti,ab OR 'developing population':ti,ab OR 'developing populations':ti,ab OR 'developing world':ti,ab OR 'less developed country':ti,ab OR 'less developed countries':ti,ab OR 'less developed nation':ti,ab OR 'less developed nations':ti,ab OR 'less developed population':ti,ab OR 'less developed populations':ti,ab OR 'less developed world':ti,ab OR 'lesser developed country':ti,ab OR 'lesser developed countries':ti,ab OR 'lesser developed nation':ti,ab OR 'lesser developed nations':ti,ab OR 'lesser developed population':ti,ab OR 'lesser developed populations':ti,ab OR 'lesser developed world':ti,ab OR 'under developed country':ti,ab OR 'under developed countries':ti,ab OR 'under developed nation':ti,ab OR 'under developed nations':ti,ab OR 'under developed population':ti,ab OR 'under developed populations':ti,ab OR 'under developed world':ti,ab OR 'underdeveloped country':ti,ab OR 'underdeveloped countries':ti,ab OR 'underdeveloped nation':ti,ab OR 'underdeveloped nations':ti,ab OR 'underdeveloped population':ti,ab OR 'underdeveloped populations':ti,ab OR 'underdeveloped world':ti,ab OR 'middle income country':ti,ab OR 'middle income countries':ti,ab OR 'middle income nation':ti,ab OR 'middle income nations':ti,ab OR 'middle income population':ti,ab OR 'middle income populations':ti,ab OR 'low income country':ti,ab OR 'low income countries':ti,ab OR 'low income nation':ti,ab OR 'low income nations':ti,ab OR 'low income population':ti,ab OR 'low income populations':ti,ab OR 'lower income country':ti,ab OR 'lower income countries':ti,ab OR 'lower income nation':ti,ab OR 'lower income nations':ti,ab OR 'lower income population':ti,ab OR 'lower income populations':ti,ab OR 'underserved country':ti,ab OR 'underserved countries':ti,ab OR 'underserved nation':ti,ab OR 'underserved nations':ti,ab OR 'underserved population':ti,ab OR 'underserved populations':ti,ab OR 'underserved world':ti,ab OR 'under served country':ti,ab OR 'under served countries':ti,ab OR 'under served nation':ti,ab OR 'under served nations':ti,ab OR 'under served population':ti,ab OR 'under served populations':ti,ab OR 'under served world':ti,ab OR 'deprived country':ti,ab OR 'deprived countries':ti,ab OR 'deprived nation':ti,ab OR 'deprived nations':ti,ab OR 'deprived population':ti,ab OR 'deprived populations':ti,ab OR 'deprived world':ti,ab OR 'poor country':ti,ab OR 'poor countries':ti,ab OR 'poor nation':ti,ab OR 'poor nations':ti,ab OR 'poor population':ti,ab OR 'poor populations':ti,ab OR 'poor world':ti,ab OR 'poorer country':ti,ab OR 'poorer countries':ti,ab OR 'poorer nation':ti,ab OR 'poorer nations':ti,ab OR 'poorer population':ti,ab OR 'poorer populations':ti,ab OR 'poorer world':ti,ab OR 'developing economy':ti,ab OR 'developing economies':ti,ab OR 'less developed economy':ti,ab OR 'less developed economies':ti,ab OR 'lesser developed economy':ti,ab OR 'lesser developed economies':ti,ab OR 'under developed economy':ti,ab OR 'under developed economies':ti,ab OR 'underdeveloped economy':ti,ab OR 'underdeveloped economies':ti,ab OR 'middle income economy':ti,ab OR 'middle income economies':ti,ab OR 'low income economy':ti,ab OR 'low income economies':ti,ab OR 'lower income economy':ti,ab OR 'lower income economies':ti,ab OR 'low gdp':ti,ab OR 'low gnp':ti,ab OR 'low gross domestic':ti,ab OR 'low gross national':ti,ab OR 'lower gdp':ti,ab OR 'lower gnp':ti,ab OR 'lower gross domestic':ti,ab OR 'lower gross national':ti,ab OR lmic:ti,ab OR lmics:ti,ab OR 'third world':ti,ab OR 'lami country':ti,ab OR 'lami countries':ti,ab OR 'transitional country':ti,ab OR 'transitional countries':ti,ab OR africa:ti,ab OR asia:ti,ab OR caribbean:ti,ab OR 'west indies':ti,ab OR 'south america':ti,ab OR 'latin america':ti,ab OR 'central america':ti,ab OR 'atlantic islands':ti,ab OR 'commonwealth of independent states':ti,ab OR 'pacific islands':ti,ab OR 'indian ocean islands':ti,ab OR 'eastern europe':ti,ab OR afghanistan:ti,ab OR albania:ti,ab OR algeria:ti,ab OR angola:ti,ab OR antigua:ti,ab OR barbuda:ti,ab OR argentina:ti,ab OR armenia:ti,ab OR armenian:ti,ab OR aruba:ti,ab OR azerbaijan:ti,ab OR bahrain:ti,ab OR bangladesh:ti,ab OR barbados:ti,ab OR benin:ti,ab OR byelarus:ti,ab OR byelorussian:ti,ab OR belarus:ti,ab OR belorussian:ti,ab OR belorussia:ti,ab OR belize:ti,ab OR bhutan:ti,ab OR bolivia:ti,ab OR bosnia:ti,ab OR herzegovina:ti,ab OR hercegovina:ti,ab OR botswana:ti,ab OR brasil:ti,ab OR brazil:ti,ab OR bulgaria:ti,ab OR 'burkina faso':ti,ab OR 'burkina fasso':ti,ab OR 'upper volta':ti,ab OR burundi:ti,ab OR urundi:ti,ab OR cambodia:ti,ab OR 'khmer republic':ti,ab OR kampuchea:ti,ab OR cameroon:ti,ab OR cameroons:ti,ab OR cameron:ti,ab OR camerons:ti,ab OR cameroun:ti,ab OR 'cape verde':ti,ab OR 'central african republic':ti,ab OR chad:ti,ab OR chile:ti,ab OR china:ti,ab OR colombia:ti,ab OR comoros:ti,ab OR 'comoro islands':ti,ab OR comores:ti,ab OR mayotte:ti,ab OR congo:ti,ab OR zaire:ti,ab OR 'costa rica':ti,ab OR 'cote divoire':ti,ab OR 'ivory coast':ti,ab OR croatia:ti,ab OR cuba:ti,ab OR cyprus:ti,ab OR czechoslovakia:ti,ab OR 'czech republic':ti,ab OR slovakia:ti,ab OR 'slovak republic':ti,ab OR djibouti:ti,ab OR 'french somaliland':ti,ab OR dominica:ti,ab OR 'dominican republic':ti,ab OR 'east timor':ti,ab OR 'east timur':ti,ab OR 'timor leste':ti,ab OR ecuador:ti,ab OR egypt:ti,ab OR 'united arab republic':ti,ab OR 'el salvador':ti,ab OR eritrea:ti,ab OR estonia:ti,ab OR ethiopia:ti,ab OR fiji:ti,ab OR gabon:ti,ab OR 'gabonese republic':ti,ab OR gambia:ti,ab OR gaza:ti,ab OR 'georgia republic':ti,ab OR 'georgian republic':ti,ab OR ghana:ti,ab OR 'gold coast':ti,ab OR greece:ti,ab OR grenada:ti,ab OR guatemala:ti,ab OR guinea:ti,ab OR guam:ti,ab OR guiana:ti,ab OR guyana:ti,ab OR haiti:ti,ab OR honduras:ti,ab OR hungary:ti,ab OR india:ti,ab OR maldives:ti,ab OR indonesia:ti,ab OR iran:ti,ab OR iraq:ti,ab OR 'isle of man':ti,ab OR jamaica:ti,ab OR jordan:ti,ab OR kazakhstan:ti,ab OR kazakh:ti,ab OR kenya:ti,ab OR kiribati:ti,ab OR korea:ti,ab OR kosovo:ti,ab OR kyrgyzstan:ti,ab OR kirghizia:ti,ab OR 'kyrgyz republic':ti,ab OR kirghiz:ti,ab OR kirgizstan:ti,ab OR 'lao pdr':ti,ab OR laos:ti,ab OR latvia:ti,ab OR lebanon:ti,ab OR lesotho:ti,ab OR basutoland:ti,ab OR liberia:ti,ab OR libya:ti,ab OR lithuania:ti,ab OR macedonia:ti,ab OR madagascar:ti,ab OR 'malagasy republic':ti,ab OR malaysia:ti,ab OR malaya:ti,ab OR malay:ti,ab OR sabah:ti,ab OR sarawak:ti,ab OR malawi:ti,ab OR nyasaland:ti,ab OR mali:ti,ab OR malta:ti,ab OR 'marshall islands':ti,ab OR mauritania:ti,ab OR mauritius:ti,ab OR 'agalega islands':ti,ab OR 'melanesia':ti,ab OR mexico:ti,ab OR micronesia:ti,ab OR 'middle east':ti,ab OR moldova:ti,ab OR moldovia:ti,ab OR moldovian:ti,ab OR mongolia:ti,ab OR morocco:ti,ab OR mozambique:ti,ab OR myanmar:ti,ab OR myanma:ti,ab OR burma:ti,ab OR namibia:ti,ab OR nepal:ti,ab OR 'netherlands antilles':ti,ab OR 'new caledonia':ti,ab OR nicaragua:ti,ab OR niger:ti,ab OR nigeria:ti,ab OR 'northern mariana islands':ti,ab OR oman:ti,ab OR muscat:ti,ab OR pakistan:ti,ab OR palau:ti,ab OR palestine:ti,ab OR panama:ti,ab OR paraguay:ti,ab OR peru:ti,ab OR philippines:ti,ab OR philipines:ti,ab OR phillipines:ti,ab OR phillippines:ti,ab OR poland:ti,ab OR portugal:ti,ab OR 'puerto rico':ti,ab OR romania:ti,ab OR rumania:ti,ab OR roumania:ti,ab OR russia:ti,ab OR russian:ti,ab OR rwanda:ti,ab OR ruanda:ti,ab OR 'saint kitts':ti,ab OR 'st kitts':ti,ab OR nevis:ti,ab OR 'saint lucia':ti,ab OR 'st lucia':ti,ab OR 'saint vincent':ti,ab OR 'st vincent':ti,ab OR grenadines:ti,ab OR samoa:ti,ab OR 'samoan islands':ti,ab OR 'navigator island':ti,ab OR 'navigator islands':ti,ab OR 'sao tome':ti,ab OR 'saudi arabia':ti,ab OR senegal:ti,ab OR serbia:ti,ab OR montenegro:ti,ab OR seychelles:ti,ab OR 'sierra leone':ti,ab OR slovenia:ti,ab OR 'sri lanka':ti,ab OR ceylon:ti,ab OR 'solomon islands':ti,ab OR somalia:ti,ab OR sudan:ti,ab OR suriname:ti,ab OR surinam:ti,ab OR swaziland:ti,ab OR syria:ti,ab OR syrian:ti,ab OR tajikistan:ti,ab OR tadzhikistan:ti,ab OR tadjikistan:ti,ab OR tadzhik:ti,ab OR tanzania:ti,ab OR thailand:ti,ab OR togo:ti,ab OR 'togolese republic':ti,ab OR tonga:ti,ab OR trinidad:ti,ab OR tobago:ti,ab OR tunisia:ti,ab OR turkey:ti,ab OR turkmenistan:ti,ab OR turkmen:ti,ab OR tuvalu:ti,ab OR uganda:ti,ab OR ukraine:ti,ab OR uruguay:ti,ab OR ussr:ti,ab OR 'soviet union':ti,ab OR 'union of soviet socialist republics':ti,ab OR uzbekistan:ti,ab OR uzbek OR vanuatu:ti,ab OR 'new hebrides':ti,ab OR venezuela:ti,ab OR vietnam:ti,ab OR 'viet nam':ti,ab OR 'west bank':ti,ab OR yemen:ti,ab OR yugoslavia:ti,ab OR zambia:ti,ab OR zimbabwe:ti,ab OR rhodesia:ti,ab OR 'developing country'/exp OR 'africa'/de OR 'africa south of the sahara'/de OR 'central africa'/de OR 'asia'/de OR 'southeast asia'/de OR 'caribbean'/de OR 'caribbean islands'/de OR 'south america'/de OR 'south and central america'/de OR 'atlantic islands'/de OR 'ussr'/de OR 'pacific islands'/de OR 'indian ocean'/de OR 'eastern europe'/de OR 'afghanistan'/exp OR 'albania'/exp OR 'algeria'/exp OR 'american samoa'/exp OR 'angola'/exp OR 'antigua and barbuda'/exp OR 'argentina'/exp OR 'armenia'/exp OR 'azerbaijan'/exp OR 'bahrain'/exp OR 'baltic states'/exp OR 'bangladesh'/exp OR 'barbados'/exp OR 'benin'/exp OR 'belarus'/exp OR 'belize'/exp OR 'bhutan'/exp OR 'bolivia'/exp OR 'bosnia and herzegovina'/exp OR 'botswana'/exp OR 'brazil'/exp OR 'bulgaria'/exp OR 'burkina faso'/exp OR 'burundi'/exp OR 'cambodia'/exp OR 'cameroon'/exp OR 'cape verde' OR 'central african republic'/exp OR 'chad'/exp OR 'chile'/exp OR 'china'/exp OR 'colombia'/exp OR 'comoros'/exp OR 'congo'/exp OR 'costa rica'/exp OR 'cote divoire' OR 'croatia'/exp OR 'cuba'/exp OR 'cyprus'/exp OR 'czechoslovakia'/exp OR 'czech republic'/exp OR 'slovakia'/exp OR 'djibouti'/exp OR 'democratic republic congo'/exp OR 'north korea'/exp OR 'dominica'/exp OR 'dominican republic'/exp OR 'dominican (dominican republic)'/exp OR 'timor leste'/exp OR 'ecuador'/exp OR 'egypt'/exp OR 'el salvador'/exp OR 'eritrea'/exp OR 'estonia'/exp OR 'ethiopia'/exp OR 'equatorial guinea'/exp OR 'fiji'/exp OR 'french guiana'/exp OR 'gabon'/exp OR 'gambia'/exp OR 'georgia (republic)'/exp OR 'ghana'/exp OR 'greece'/exp OR 'grenada'/exp OR 'guatemala'/exp OR 'guinea'/exp OR 'guinea bissau'/exp OR 'guam'/exp OR 'guyana'/exp OR 'haiti'/exp OR 'honduras'/exp OR 'hungary'/exp OR 'samoa'/exp OR 'india'/exp OR 'indonesia'/exp OR 'iran'/exp OR 'iraq'/exp OR 'jamaica'/exp OR 'jordan'/exp OR 'kazakhstan'/exp OR 'kenya'/exp OR 'south korea'/exp OR 'kyrgyzstan'/exp OR 'laos'/exp OR 'latvia'/exp OR 'lebanon'/exp OR 'lesotho'/exp OR 'liberia'/exp OR 'libyan arab jamahiriya'/exp OR 'lithuania'/exp OR 'macedonia (republic)'/exp OR 'madagascar'/exp OR 'malawi'/exp OR 'malaysia'/exp OR 'mali'/exp OR 'malta'/exp OR 'mauritania'/exp OR 'mauritius'/exp OR 'melanesia'/exp OR 'mexico'/exp OR 'federated states of micronesia'/exp OR 'middle east'/de OR 'moldova'/exp OR 'mongolia'/exp OR 'morocco'/exp OR 'mozambique'/exp OR 'myanmar'/exp OR 'namibia'/exp OR 'nepal'/exp OR 'netherlands antilles'/exp OR 'new caledonia'/exp OR 'nicaragua'/exp OR 'niger'/exp OR 'nigeria'/exp OR 'oman'/exp OR 'pakistan'/exp OR 'palau'/exp OR 'panama'/exp OR 'papua new guinea'/exp OR 'paraguay'/exp OR 'peru'/exp OR 'philippines'/exp OR 'poland'/exp OR 'portugal'/exp OR 'puerto rico'/exp OR 'romania'/exp OR 'russian federation'/exp OR 'rwanda'/exp OR 'saint kitts and nevis'/exp OR 'saint lucia'/exp OR 'saint vincent and the grenadines'/exp OR 'saudi arabia'/exp OR 'senegal'/exp OR 'serbia'/exp OR 'montenegro (republic)'/exp OR 'seychelles'/exp OR 'sierra leone'/exp OR 'slovenia'/exp OR 'sri lanka'/exp OR 'somalia'/exp OR 'south africa'/exp OR 'sudan'/exp OR 'suriname'/exp OR 'swaziland'/exp OR 'syrian arab republic'/exp OR 'tajikistan'/exp OR 'tanzania'/exp OR 'thailand'/exp OR 'togo'/exp OR 'tonga'/exp OR 'trinidad and tobago'/exp OR 'tunisia'/exp OR 'turkey (republic)'/exp OR 'turkmenistan'/exp OR 'uganda'/exp OR 'ukraine'/exp OR 'uruguay'/exp OR 'uzbekistan'/exp OR 'vanuatu'/exp OR 'venezuela'/exp OR 'viet nam'/exp OR 'yemen'/exp OR 'yugoslavia'/exp OR 'yugoslavia (pre-1992)' OR 'zambia'/exp OR 'zimbabwe'/exp) AND

(stigma:ti,ab OR stigmatize:ti,ab OR stigmatise:ti,ab OR stigmatizing:ti,ab OR stigmatizing:ti,ab OR stereotype:ti,ab OR stereotyping:ti,ab OR shame:ti,ab OR shaming:ti,ab OR discrimination:ti,ab OR discriminating:ti,ab OR ‘social distance’:ti,ab OR prejudice:ti,ab OR blame:ti,ab)

AND

[2008-2017]/py
